# A Tandem Duplicate of Anti-Müllerian Hormone with a Missense SNP on the Y Chromosome Is Essential for Male Sex Determination in Nile Tilapia, *Oreochromis niloticus*

**DOI:** 10.1371/journal.pgen.1005678

**Published:** 2015-11-20

**Authors:** Minghui Li, Yunlv Sun, Jiue Zhao, Hongjuan Shi, Sheng Zeng, Kai Ye, Dongneng Jiang, Linyan Zhou, Lina Sun, Wenjing Tao, Yoshitaka Nagahama, Thomas D. Kocher, Deshou Wang

**Affiliations:** 1 Key Laboratory of Freshwater Fish Reproduction and Development (Ministry of Education), Key Laboratory of Aquatic Science of Chongqing, School of Life Science, Southwest University, Chongqing, China; 2 Solution-Oriented Research for Science and Technology (SORST), Laboratory of Reproductive Biology, National Institute for Basic Biology, Okazaki, Japan; South Ehime Fisheries Research Center, Ehime University, Matsuyama, Japan; 3 Department of Biology, University of Maryland, College Park, Maryland, United States of America; National Institute for Basic Biology, JAPAN

## Abstract

Variation in the TGF-β signaling pathway is emerging as an important mechanism by which gonadal sex determination is controlled in teleosts. Here we show that *amhy*, a Y-specific duplicate of the *anti-Müllerian hormone* (*amh*) gene, induces male sex determination in Nile tilapia. *amhy* is a tandem duplicate located immediately downstream of *amhΔ-y* on the Y chromosome. The coding sequence of *amhy* was identical to the X-linked *amh* (*amh*) except a missense SNP (C/T) which changes an amino acid (Ser/Leu92) in the N-terminal region. *amhy* lacks 5608 bp of promoter sequence that is found in the X-linked *amh* homolog. The *amhΔ-y* contains several insertions and deletions in the promoter region, and even a 5 bp insertion in exonVI that results in a premature stop codon and thus a truncated protein product lacking the TGF-β binding domain. Both *amhy* and *amhΔ-y* expression is restricted to XY gonads from 5 days after hatching (dah) onwards. CRISPR/Cas9 knockout of *amhy* in XY fish resulted in male to female sex reversal, while mutation of *amhΔ-y* alone could not. In contrast, overexpression of Amhy in XX fish, using a fosmid transgene that carries the *amhy/amhΔ-y* haplotype or a vector containing *amhy* ORF under the control of CMV promoter, resulted in female to male sex reversal, while overexpression of AmhΔ-y alone in XX fish could not. Knockout of the *anti-Müllerian hormone receptor type II* (*amhrII*) in XY fish also resulted in 100% complete male to female sex reversal. Taken together, these results strongly suggest that the duplicated *amhy* with a missense SNP is the candidate sex determining gene and *amhy/amhrII* signal is essential for male sex determination in Nile tilapia. These findings highlight the conserved roles of TGF-β signaling pathway in fish sex determination.

## Author Summary

Unlike mammals, the identity of the master sex-determining gene varies among fish species, and it is not yet clear if there is a common molecular pathway regulating gonadal sex determination across teleosts. Here we show that a Y-linked duplicate of the *anti-Mullerian hormone* (*amhy*) is essential for male sex determination in tilapia. Mutation of *amhy* resulted in male to female sex reversal, while overexpression of it resulted in female to male sex reversal. A missense single nucleotide polymorphisms (SNP) (C/T) in the open reading frame (ORF) of *amhy* might contribute to male sex determination in tilapia. Knockout of the *anti-Müllerian hormone receptor type II* (*amhrII*) also resulted in male to female sex reversal. Taken the *amhy* in Patagonian pejerrey, *amhrII* in *Takifugu rubripes*, *gsdf* ^Y^ in *Oryzias luzonensis* into consideration, these data highlight an important role for TGF-β signaling in teleost sex determination.

## Introduction

Master sex-determining (SD) genes are the key genetic switches controlling the gonadal sex differentiation cascade leading to the development of either ovaries or testes. To date, master SD genes have been identified in only a few vertebrate species. *SRY*/*Sry* was the first sex determiner identified in mammals [1, 2]. With the recent discovery that *sox3* ^Y^ is the sex determiner in *Oryzias dancena* [3], Sox genes continue to figure prominently in discussions of vertebrate sex determination. *Doublesex*/*mab* (DM) related genes have been associated with sex determination in a wide range of species, including *Dmrt1* in chicken and half-smooth tongue sole [4, 5], *DM-W* in African clawed frog [6], and *dmy*/*dmrt1bY* in *Oryzias latipes* [7, 8]. Other genes have been implicated as master sex determiners in particular lineages, including *FOXL2* in goat [9], and *sdY* (*irf9y*) in rainbow trout [10]. Several recent studies have suggested that components of the transforming growth factor beta (TGF-β) signaling pathway are involved in sex determination in fishes. These include a Y-linked duplicate of the *anti-Müllerian hormone* (*amhy*) in the Patagonian pejerrey [11], a mutation in the *amh* receptor (*amhrII*) in *Takifugu rubripes* [12], and a Y-linked duplicate of a related ligand, *gonadal soma derived growth factor* (*gsdf* ^Y^) in *Oryzias luzonensis* [13]. These findings suggest a critical role for TGF-β signaling in gonadal sex determination in teleosts.

Studies of mammalian sex chromosomes have provided significant insights into the evolution of sex determination, but SD genes have not yet been identified in the vast majority of vertebrates. For example, teleost fishes make up nearly half of all living vertebrate species and show a wide variety of sex determination mechanisms [14], but only a handful of these sex determiners have been identified. Closely related species of fish frequently segregate different master sex determiners, suggesting that a delicately balanced network of gene interactions controls sex determination. For example, three different genes (*dmy*, *gsdf* ^Y^ and *sox3* ^Y^) have been identified as master sex determiners among closely related species of ricefish [3, 7, 8, 13]. Master sex determiners map to three different chromosomes among closely related species of stickleback [15]. Recent work has identified at least three sex determiners among strains of zebrafish [16, 17].

Numerous studies have investigated the mechanisms of sex determination in tilapia (*Oreochromis niloticus*), motivated in part by commercial interest in the higher growth rates of all-male progenies. Tilapia are gonochoristic teleosts in which sex is largely genetically determined [18], although environmental factors also play a role [19]. XX/XY sex determination systems have been described on both LG1 and LG23 in this species [20, 21]. All-XX and all-XY progenies can be obtained by crossing normal XX females to either experimentally sex-reversed XX pseudomales or YY supermales respectively [22].

A previous report identified several sex-linked markers near the *amh* gene on LG23 [21]. More recent studies identified a Y-linked duplication of *amh* on LG23, termed a male-specific *amhy*, differing from the sequence of *amh* by a 233 bp deletion in exonVII [23]. Our own analyses have identified five additional sex-linked markers on LG23 that map very close to *amh* [22]. *amh* is located in the center of this sex-linked region and shows sexually dimorphic expression in the gonads at 3 days post fertilization [24], making it an interesting gene for sex determination in this species.

*Amh* is responsible for the regression of Müllerian ducts in tetrapods [25]. It is also found in teleost fish despite the fact that they do not have Müllerian ducts [23, 26–28]. In mammals, Amh functions primarily through the type II receptor AmhrII [25]. Mutations of *amhrII* in medaka and *Takifugu rubripes* result in male to female sex reversal [12, 29]. These studies suggested that *amh*/*amhrII* signaling might play a role in fish sex determination.

Recent efforts have generated a number of important resources for tilapia research, including a genome sequence, a microarrayed fosmid library, and several gonadal transcriptomes [30–32]. TALEN and CRISPR/Cas9 gene knockout technologies have also been established in tilapia [33, 34]. The availability of these tools prompted us to try to isolate the SD gene in the Nile tilapia. In the present study, we isolated a Y-specific duplicate of the *amh* gene, designated as *amhy*, and confirmed its male-specific (XY and YY) expression by transcriptome analysis and Western blot. We used transgenic techniques to overexpress *amhy* in XX fish, and we used CRISPR/Cas9 mutagenesis to knockout *amhy*, and its putative receptor *amhrII* in XY fish. Our results suggest a conserved role for the TGF-βsignaling pathway in sex determination of vertebrates.

## Results

### Identification of a Y-linked duplication of *amh*

**Fig 1:**
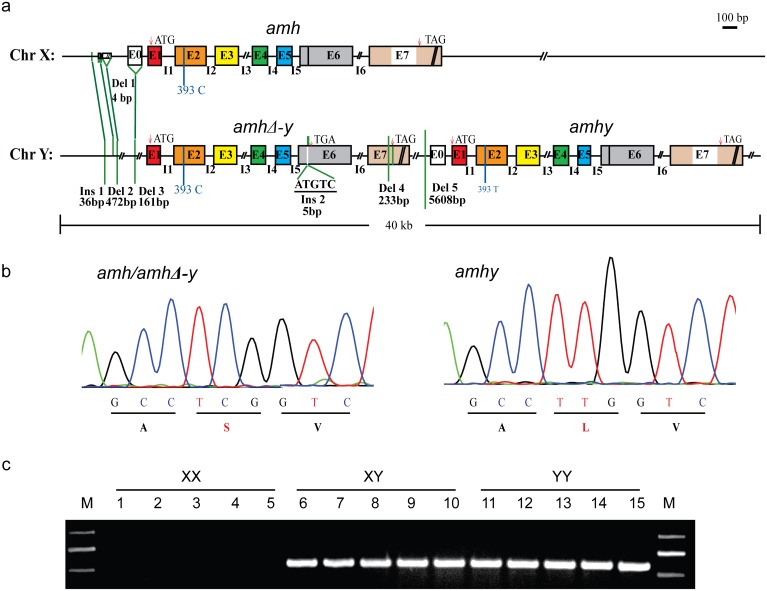
Schematic representation of *amh/amhΔ-y/amhy* gene structure on the Y and X chromosome. (**a**) Gene structure of *amh*, *amhΔ-y* and *amhy*. *amhy* was tandemly located downstream of *amhΔ-y* on the Y chromosome, with subsequent deletion of 5608 bp in the promoter. Compared with the X-linked *amh*, the transcribed region of *amhΔ-y* includes a 5 bp (ATGTC) insertion in exonVI and a deletion of 233 bp in exonVII. There are also three deletions (472, 161 and 4bp) and one insertion (36 bp) in the upstream of *amhΔ-y* start codon. (**b**) A missense SNP (C/T) was identified in the exonII of *amh* and *amhy*. This SNP converts amino acid from Serine to Leucine in the N-terminal region. (**c**) Genomic PCR amplification of the Y-specific fragments using an *amhΔ-y* specific primer F2 designed on the 5 bp insertion of *amhΔ-y* and a reverse primer R2 shared by *amh*, *amhΔ-y* and *amhy* (*amhΔ-y-*F2/R2). A 547 bp band was observed in XY and YY, but not XX genomic DNA.

We used PCR to screen a microarrayed tilapia XY genomic library for a sex-specific marker. We identified X-specific and Y-specific fosmid clones (designated X278 and Y156) producing amplimers of 1422 and 982 bp, respectively (S1 Fig). The two fosmid clones were then sequenced using Illumina HiSeq2000 technology and carefully assembled using local Basic Local Alignment Search Tool (local BLAST) by hand based on the sequence differences of several genomic PCR products amplified from XX and YY individuals and the sequence differences between Y156 and X278 fosmid. Sequence analysis revealed that an *amh* gene, termed as *amh*, was present in the X278 fosmid clone. By gene prediction with GENSCAN and BLAST search in the tilapia genome using the assembled sequence of the Y 156 clone, only two genes, designated as *amhy* and *amhΔ-y*, were found in the Y156 fosmid clone. *amhy* is a tandem duplicate located immediately downstream of *amhΔ-y* (Fig 1a). The insertion of Y156 was about 40 kb, which was further confirmed by sequencing 25 fragments, each about 3 kb with partial overlapping ends.

A comparison of the sequences of the two clones revealed numerous differences. The coding sequence of *amhy* was identical to the X-linked *amh* except a single nucleotide polymorphisms (SNP) (C/T) in exonII, which changes an amino acid (Ser/Leu92) in the N-terminal region. *amhy* has lost 5608 bp of promoter sequence that is found in the X-linked *amh* homolog (Fig 1a and 1b). *amhΔ-y* contains a 5 bp (ATGTC) insertion in exonVI, resulting in a frameshift mutation and a premature stop codon. The 5 bp insertion could be easily confirmed by Taq^α^ I restriction enzyme digestion of the genomic PCR fragments spanning it, which resulted in two digested bands in the XY and YY fish, whereas no band was produced in XX fish (S2 Fig). *amhΔ-y* also has a deletion of 233 bp in exonVII that further precludes translation of the protein motif that binds to the TGF-βreceptor. There was one additional insertion (36 bp) and three other deletions (4, 472 and 161 bp, respectively) at -1972, -1756, -1664 and -625, respectively, of *amhΔ-y* start codon (ATG, A as 0 point), compared with *amh* on the X chromosome (Fig 1a). These differences between *amhy*, *amh* and *amhΔ-y* were further demonstrated by PCR amplification in XX, XY and YY genomic DNA pools (S3 Fig). The gene sequences of *amhy*, *amhΔ-y* and *amh* are shown in the S1 Sequence.

Primers flanking differentiated sequences in the promoter and exon region were used for PCR-based sex genotyping. Y-specific primers amplified fragments in the XY and YY pooled genomic DNA respectively, but not in the pooled XX genomic DNA (Fig 1c). Genotypic sex based on these primers showed 100% correspondence with phenotypic sex based on gonadal histology in 300 individuals derived from four crosses (S1 Table).

Finally, there are five SNPs in the coding sequences of *amhΔ-y* and *amhy*. Three of them are non-synonymous and the amino acid changed (S4 Fig). *amhΔ-y* is close to neutral evolution in comparison to *amhy* (Ka/Ks<1) (S2 Table). The tilapia *amhΔ-y* cDNA isolated by rapid amplification of cDNA ends (RACE) is 2,065 bp long with a 46 bp 5' untranslated region (UTR), a 1,242 bp 3' UTR and an ORF of 777 bp encoding a putative protein of 258 aa (amino acid) without the TGF-β domain. The *amh/amhy* isolated by RACE has a 346 bp 5' UTR, a 782 bp 3' UTR and an ORF of 1,545 bp, encoding a putative protein of 514 aa with the TGF-β domain.

### *amh*, *amhy* and *amhrII* are expressed in tilapia gonads

**Fig 2:**
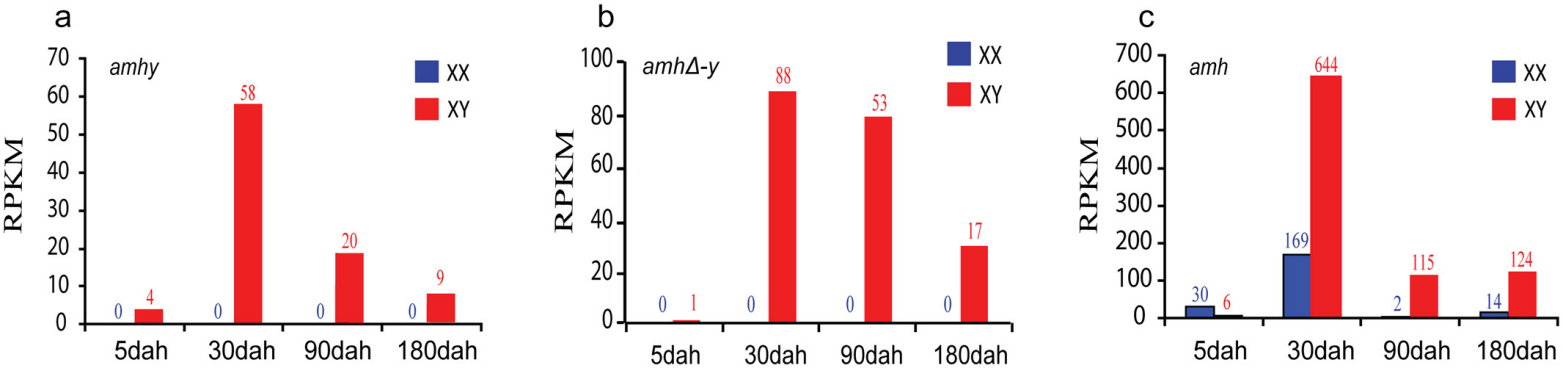
Expression profiles of *amhy*, *amhΔ-y* and *amh* by transcriptome analysis. Transcriptome analysis revealed that *amhy* and *amhΔ-y* transcripts were only detected in XY gonads, with low expression at 5 days after hatching (dah), peaked at approximately 30 dah, and decreased expression at 90 dah (**a**, **b**). *amh* expression was observed in both XX and XY gonads with significant higher expression in XX than in XY gonads from 5 dah onwards, while it showed higher expression in XY than in XX gonads at 30 dah onwards (**c**). The expression of *amh* and *amhy* were analyzed by counting reads with the SNP (C/T) in exonII. The expression of *amhΔ-y* was analyzed by blast in the transcriptome data with the *amhΔ-y* specific 5 bp (ATGTC) insertion in exonVI. dah, days after hatching. A normalized measure of RPKM (reads per kb per million reads) was used to normalize the expression profiles of *amhy*, *amhΔ-y* and *amh*. The numbers over the bars indicate RPKM.

RT-PCR showed that among the twelve tissues examined, *amh/amhy* and *amhrII* were expressed exclusively in gonads, with greater expression in testis. *amhΔ-y* was expressed only in the XY testis, not in the XX ovary (S5 Fig). Transcriptome analysis revealed that *amhy* and *amhΔ-y* transcripts were only detected in XY gonads, with expression at 5 days after hatching (dah), the critical period for molecular sex determination in Nile tilapia, peaked at approximately 30 dah, and decreased at 90 and to very low level at 180 dah (Fig 2a and 2b). Similar expression profiles of *amh* and *amhrII* were observed in both XX and XY gonads, with significantly higher expression in XX than in XY gonads at 5 dah, while they showed higher expression in XY than in XX gonads at 30 dah onwards (Fig 2c and S6 Fig).

Specificity of the Amh ployclonal antibody was characterized by Western blot analysis using recombinant protein (both purified and unpurified), and the proteins extracted from the XX ovary, XY testis, and YY testis. The Amh antibody can recognize the Amh, AmhΔ-y and Amhy protein. The blots revealed specific bands of ~54 kDa, corresponding to the calculated molecular weight of Amh/Amhy, in the protein samples extracted from the XX ovary, XY and YY testis. Another band of ~27 kDa, corresponding to AmhΔ-y, was detected exclusively in the protein samples extracted from the XY and YY testis. Amh/Amhy was detected in XY gonads of 5, 10, 30, 90, 180 and 300 dah tilapia, and also with expression of Amh in XX tilapia at these stages, while AmhΔ-y was only detected in 5, 10, 30 and 90 dah XY gonads. In addition, Amhy and AmhΔ-y were also detected in the proteins from YY gonads of 30 and 90 dah tilapia (S7 Fig). By immunohistochemistry, the Amh/AmhΔ-y/Amhy and AmhrII proteins were located in somatic cells surrounding germ cells in the XY gonads at 5 dah. At later stages, Amh/AmhΔ-y/Amhy is observed in myoid cells and Sertoli cells, while AmhrII was observed in spermatogonia and Sertoli cells of the testis at 30, 90 and 180 dah (S8 Fig).

### Amhy knockout by CRISPR/Cas9 resulted in male to female sex reversal in XY fish

**Fig 3:**
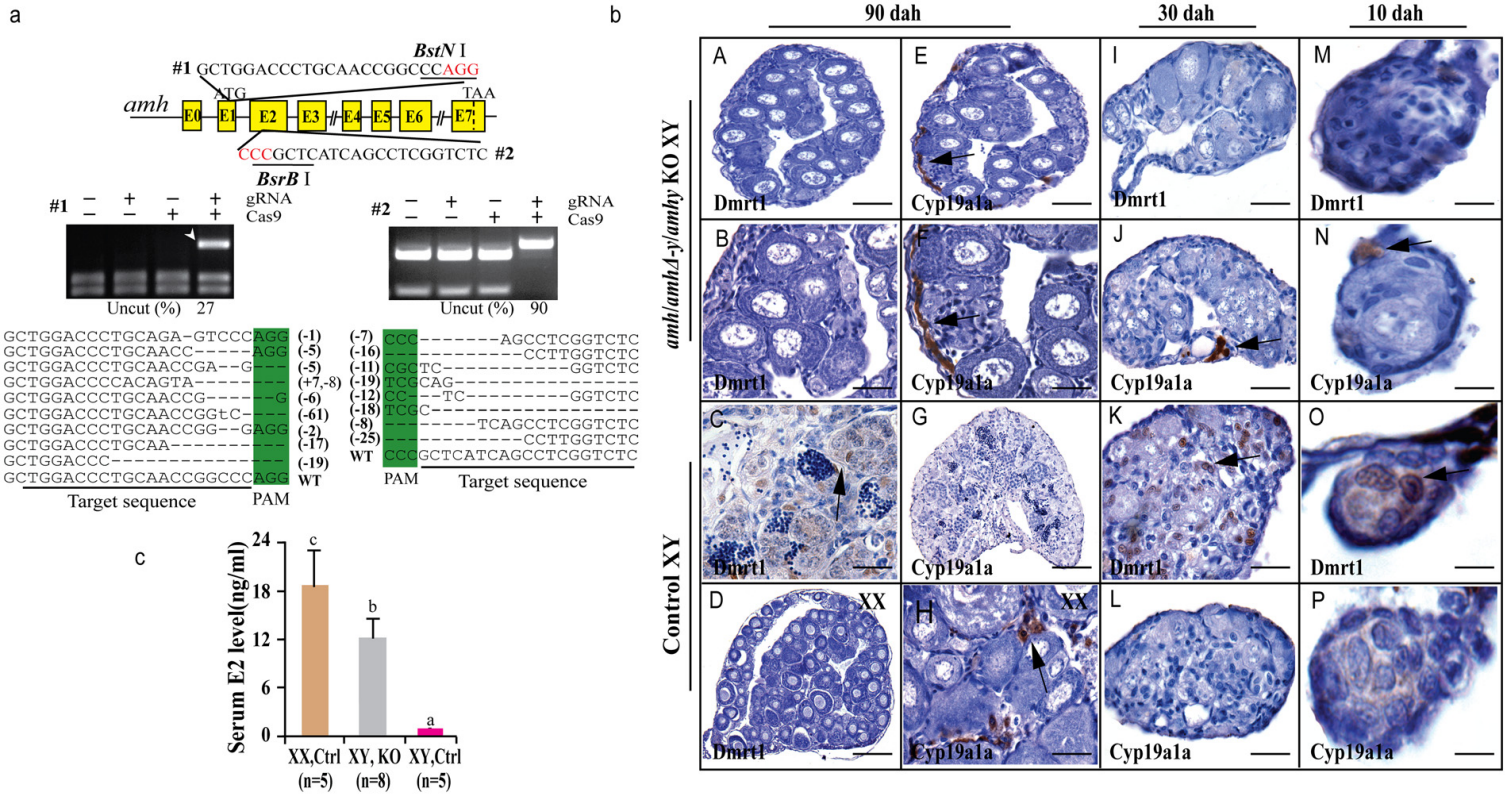
Knockout of Amhy/AmhΔ-y/Amh by CRISPR/Cas9 resulted in male to female sex reversal in F_0_ XY fish. (**a**) Knockout of *amhy*/*amhΔ-y*/*amh* was achieved by CRISPR/Cas9. Two target sites were selected in the exon II and III of *amhy*/*amhΔ-y*/*amh*. Target site 1 and 2 were shared by *amh*, *amhΔ-y* and *amhy*. The two target sites included *BstN* I and *BsrB* I adjacent to the protospacer adjacent motif (PAM) for mutation analysis, respectively. For each target, digestion with the appropriate enzyme (*BstN* I, *Bsr*B I) produced two cleavage bands in the control group, while an intact DNA fragment (indicated by white arrowheads) was observed in embryos injected with both Cas9 mRNA and target gRNA. Mutation sequences from the uncleaved bands were listed. The percentage of un-cleaved (i.e. mutant) DNA is shown for each target site. The indel frequency was calculated by dividing uncleaved band intensity to the total band intensity of the restriction enzyme digestion of pooled genomic DNA from up to 20 embryos. (**b**) The *amh*/*amhΔ-y*/*amhy* knockout F_0_ XY fish showed male to female sex reversal, as demonstrated by gonad histology and immunohistochemistry. Cyp19a1a, which was expressed in the control XX gonads but not XY gonads (**G**, **L**, **P**), was found to be expressed in the sex-reversed XY gonads at 10, 30 and 90 dah (**E, F, H, J, N**). In contrast, Dmrt1 disappeared in the sex-reversed XY gonads, which was different than the control testis at 10, 30 and 90 dah (**A-D, I, M, K, O**). (**c**) Higher serum E2 was observed in the sex-reversed XY fish compared with the XY control. Results are presented as the mean±SD. Different letters (a, b, c) indicate statistical differences at P<0.05 as determined by one-way ANOVA, followed by Tukey/Kramer post hoc test. Sample numbers are shown on the figure.

Two guide RNAs (gRNA) were designed, in exons II and III of *amhy/amh/amhΔ-y*, to increase the likelihood of successful targeting and also exclude the effects of off-target events on the phenotypes. The gRNAs contained *BstN* I or *BsrB* I sites adjacent to the protospacer adjacent motif (PAM) sequence for mutation analysis. Restriction enzyme digestion and Sanger sequencing were performed to confirm the insertions or deletions (indels), including both in-frame and frame-shift mutations, in pools of randomly selected embryos (Fig 3a). The screening results showed that 18% (8/45) of target 1 and 62% (28/45) of target 2 fish were mutated (S3 Table). Due to the high homology of *amhy*, *amhΔ-y* and *amh*, CRISPR/Cas9 disrupted three genes at both target sites. Restriction enzyme digestion showed that the CRISPR system disrupt *amhy* and *amhΔ-y* genes equally in F_0_ knockout XY fish (S9 Fig). RT-PCR with forward primers located in the targets using cDNA from 90 dah mutated gonads as templates demonstrated that the expression of *amh*/*amhΔ-y*/*amhy* mRNA in the F_0_ knockout group was much lower than that of control group (S10A Fig). Consistent with this, dramatically decreased Amh/Amhy protein levels and no AmhΔ-y were detected in the gonads of F_0_ knockout fish by Western blot (S10B Fig).

The gonads of the mutagenized fish were subjected to both histological and immunohistochemical (IHC) analyses at 10, 30 and 90 dah. Macroscopic observation of the gonads of 3-month-old gRNA/Cas9 microinjected XY fish revealed that some of the mutated F_0_ fish showed male to female sex reversal. Gonadal sex differentiation in the sex-reversed XY fish was characterized by the formation of the ovarian cavity (OC) and the appearance of phase II oocytes. These gonads were topologically indistinguishable from the control XX ovary, but obviously different from the control XY testis (Fig 3b). IHC revealed that like the control ovary, male Sertoli cell marker Dmrt1 was not expressed in the sex-reversed XY gonads (Fig 3bA–3bD). Additionally, female specific marker Cyp19a1a was expressed in these sex-reversed XY gonads, like the control ovary (Fig 3bE–3bH). Consistent with the IHC results, higher serum estrogen (E2) was observed in the sex-reversed XY fish compared with the XY control (Fig 3c). The effects of *amh*/*amhΔ-y*/*amhy* deficiency on sex determination could be detected from a very early stage by IHC analysis of sexual dimorphic proteins such as Dmrt1 and Cyp19a1a. Tracing back, Cyp19a1a, which was not expressed in XY gonads at 10 and 30 dah, was detected in the sex-reversed XY gonads as early as 10 and 30 dah (Fig 3bJ, 3bN, 3bL and 3bP). In contrast, Dmrt1, which was detected in the control XY but not in XX gonad (Fig 3bK and 3bO), was not detected in sex-reversed XY gonads (Fig 3bI and 3bM).

Both target sites resulted in sex reversal. Of the 8 F_0_ fish with mutations in target site 1, 5 (62%) displayed complete male to female sex reversal. Of the 28 F_0_ fish with mutations in target site 2, 20 (71%) showed complete sex reversal (Table 1). The genetic sex of these sex-reversed XY fish with *amh*/*amhΔ-y*/*amhy* mutation was confirmed by amplification of a sex-linked marker (SPF/SPR) and all of these sex-reversed males were XY fish (S11 Fig). In addition, a number of oogonia, as demonstrated by Gsdf staining, and only a few primary and secondary oocytes, were observed in Amh deficient XX ovary at 90 dah (S12A Fig), compared with the control (S12B Fig).

**Table 1:** Phenotype of genetically modified fish produced in this study.

Gene	F_0_	Phenotype	
		Female (%)	Male (%)
CRISPR/Cas9 knockdowns:			
*amh/amhΔ-y/amhy-*XY*-#*1	8	5 (62)	3 (38)
*amh/amhΔ-y/amhy-*XY*-#*2	28	20 (71)	8 (29)
*amhrII-*XY*-#*1	22	22 (100)	0 (0)
*amhrII-*XY*-#*2	15	15 (100)	0 (0)
Fosmid transgenic:			
Y156-XX	25	17 (68)	8 (32)
*amhΔ-y*-ORF-XX	10	10 (100)	0 (0)
*amh*-ORF-XX	11	11 (100)	0 (0)
*amhy*-ORF-XX	14	5 (36)	9 (64)

**Fig 4:**
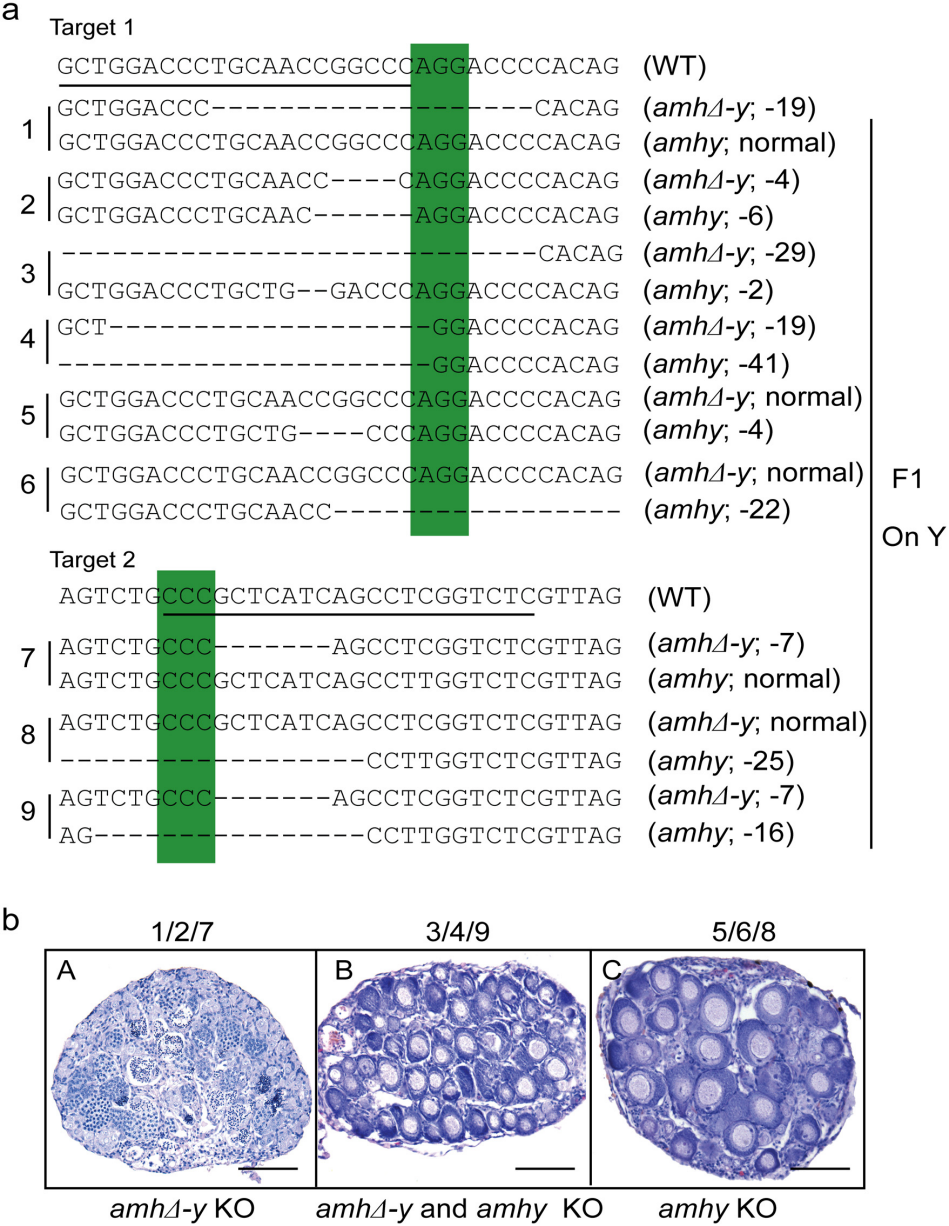
Mutation of Amhy resulted in male to female sex reversal in F_1_ XY fish. (**a**) Nine independent mutations at the target site 1 and 2 were detected in F_1_ fish: three types of *amhy* mutations, three types of *amhΔ-y* mutation and three types of *amhy* and *amhΔ-y* double mutations. (**b**) The XY fish with *amhy* mutant allele (n = 9+5) or with double mutation of *amhy* and *amhΔ-y* (n = 14+10) on the Y chromosome displayed sex reversal with typical ovary characterized by the presence of ovarian cavity (OC) and the appearance of oocytes. The XY fish with *amhΔ-y* (n = 11+7) mutant allele on the Y chromosome displayed no sex reversal with typical testis characterized by different stages of spermatogenic cells. Scale bars, 25 μm.

Sperm from the XY founders carrying different types of *amh*/*amhΔ-y*/*amhy* mutations (target 1 and 2) was mated with wild-type XX fish to produce F_1_ animals and independent mutant alleles having indels at the target of F_1_ XY were successfully obtained. These mutants caused frame shifts resulting in premature termination, predicted to yield truncated proteins (S13 Fig). The F_1_ XY fish with *amhy* mutant allele on the Y chromosome alone (n = 9+5) or double mutation of *amhy* and *amhΔ-y* (n = 14+10) displayed sex reversal with clear ovarian structure at 90 dah. In contrast, the F_1_ XY fish with mutation of *amhΔ-y* alone displayed no sex reversal with normal testis at 90 dah (n = 11+7) (Fig 4, Table 2).

**Table 2:** Phenotype of F_1_ XY fish with *amhy*/*amhΔ-y* mutations.

Founder (F_0_)	Genotype (F_1_)	Gene mutation (F_1_)	Fish number	Phenotype
#1-XY male (Target 1)	XY	*amhΔ-y*	11	Male
	XY	*amhy*	9	Female
	XY	*amhy* and *amhΔ-y*	14	Female
#2-XY male (Target 2)	XY	*amhΔ-y*	7	Male
	XY	*amhy*	5	Female
	XY	*amhy* and *amhΔ-y*	10	Female

### Overexpression of Y156 fosmid or Amhy ORF causes female to male sex reversal in F_0_ XX fish

**Fig 5:**
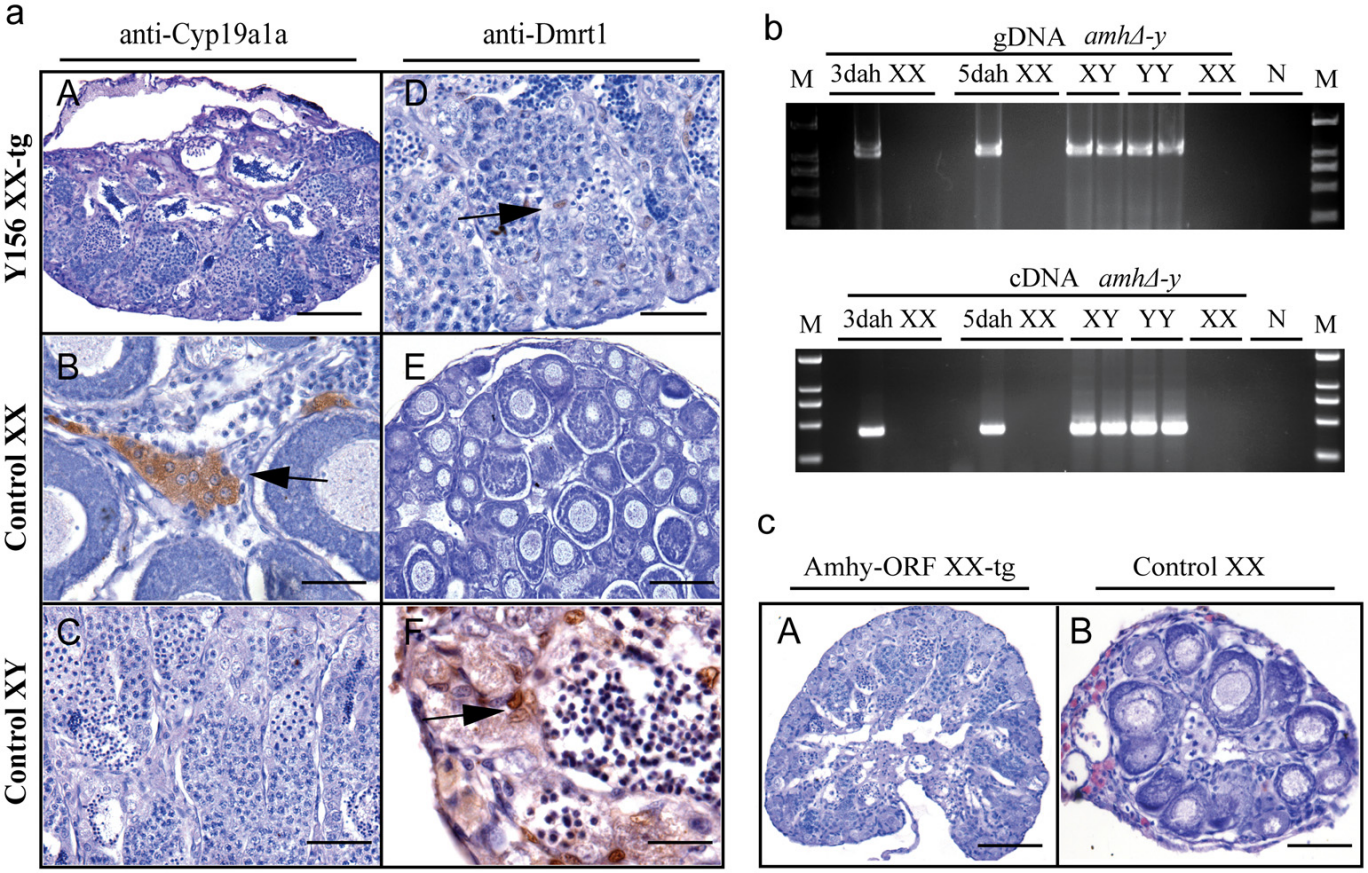
Transgenic overexpression of Y156 fosmid and Amhy ORF resulted in sex reversal in F_0_ XX fish. (**a**) The overexpression of fosmid Y156 containing *amhy* in F_0_ XX fish resulted in sex reversal (8 (31.6%) of 25), which was histologically indistinguishable from the control testis. Immunohistochemistry analysis showed that Cyp19a1a was absent from the sex-reversed XX fish gonads, like the control testis, but unlike the control ovary (**A-C**). In contrast, like the control testis but different from the control ovary, Dmrt1 was expressed in the sex-reversed XX fish gonads (**D-F**). Scale bars, 50μm (A, E); 15μm (B, D); 25μm (C); 10μm (F). (**b**) Integration of the transgene and mRNA expression in the XX fry confirmed by genomic PCR and RT-PCR using *amhΔ-y* specific primers at 3 and 5 dah. Successful transgene of *amhy* was indirectly reflected by *amhΔ-y* mRNA detection. gDNA, genomic DNA; dah, days after hatching; N, negative control. M, DNA molecular standard. (**c**) Transgenic overexpression of *amhy* ORF in F_0_ XX fish resulted in sex reversal. H.E. staining gonads from the 3-month-old XX transgenic fish displayed a clear testicular structure (n = 9), while gonads from the negative transgene fish showed typical ovarian structure (n = 21). Scale bars, 50 μm.

Gonad histological analysis revealed that 8 (32%) of 25 Y156 fosmid transgenic F_0_ XX fish displayed complete female to male sex reversal at 90 dah (Fig 5a, Table 1). The sex-reversed gonads exhibited a clear testicular structure. IHC showed that they expressed the testicular specific gene Dmrt1, and did not express the ovarian specific Cyp19a1a (Fig 5aA–5aF). The integration and mRNA expression of the transgene in the XX fry were examined and confirmed by genomic PCR and RT-PCR, using *amhΔ-y* specific primers, at 3 and 5 dah (Fig 5b).

To investigate the ability of the *amhy* ORF to induce sex reversal, we constructed overexpression vector pIRES-hrGFP-1a in which CMV controlled the *amhy* cDNA. Morphologically, the *amhy*-transgene XX F_0_ fish also displayed sex reversal with a clear testicular structure (n = 9), while no sex reversal was found in the control XX group (n = 21) at 90 dah (Fig 5c). In contrast, the *amh* or *amhΔ-y*-transgene XX F_0_ fish displayed no sex reversal with a clear ovarian structure at 90 dah (*amh*, n = 11; *amhΔ-y*, n = 10) (S14 Fig, Table 1).

### AmhrII knockout causes male to female sex reversal in XY fish

**Fig 6:**
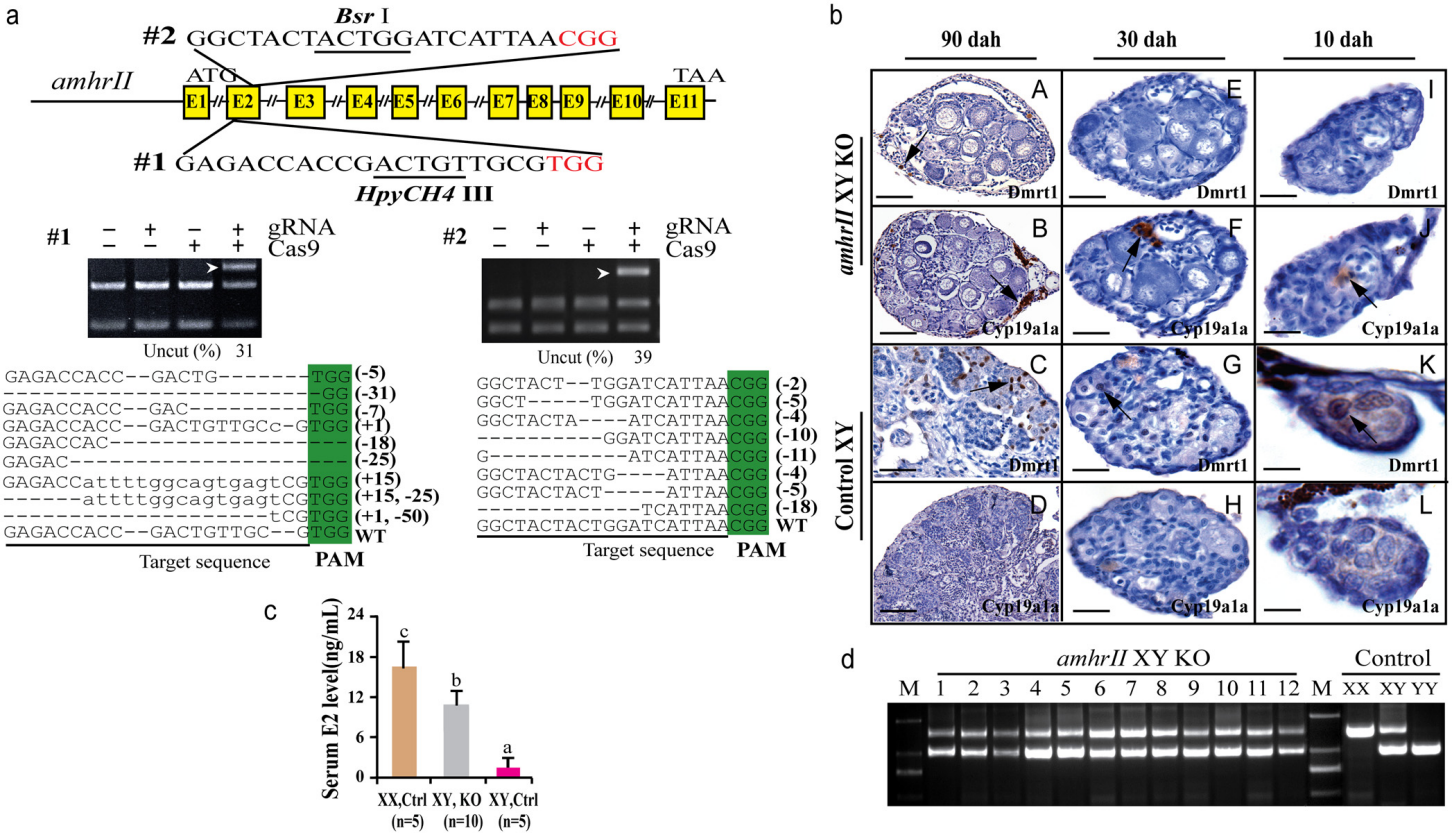
Knockout of *amhrII* by CRISPR/Cas9 resulted in male to female sex reversal in XY fish. (**a**) Two target sites were selected in the exonII and III of *amhrII*. The target sites contain *Bsr* I and *HpyCH4* III adjacent to PAM for mutation analysis. The un-cleaved bands were observed in the knockout group after *Bsr* I and *HpyCH4* III digestion, while the DNA of the control groups were completely digested. The percentage of uncleaved band is shown below the gel images. Mutation sequences from the un-cleaved bands are listed. (**b**) The *amhrII* knockout XY fish exhibited male to female sex reversal as demonstrated by gonad histology and immunohistochemistry (IHC). IHC analysis showed that, like the control XX gonads but unlike the control XY gonads, Cyp19a1a was expressed in the sex-reversed XY gonads at 10, 30 and 90 dah (**B**, **D, F**, **J, H**, **L**). In contrast, Dmrt1 was absent from the sex-reversed XY gonads, which was distinct from the control XY gonads at 10, 30 and 90 dah (**A**, **C, E**, **I, G**, **K**). (**c**) Knockout of *amhrII* in the XY fish resulted in increased serum E2 level compared with the control fish. Results are presented as the mean±SD. Different letters (a, b, c) indicate statistical differences at P<0.05 as determined by one-way ANOVA followed by Tukey/Kramer post hoc test. Sample numbers are shown. (**d**) Genotype of the sex-reversed XY fish was confirmed with a sex specific marker (SPF/SPR). 1–12, sex-reversed fish with *amhrII* knockout. M, DNA molecular standard. Scale bars, 50μm (A, B); 15μm (C, D, E-H); 10μm (I-L).

Two gRNAs were designed in the exon II and III of *amhrII* (Fig 6a), an autosomal gene located on LG20. In total, 73% (22/30) of target 1 and 50% (15/30) of target 2 were mutated by CRISPR/Cas9. The mutation frequencies within individuals ranged from 22% to 68% (S3 Table). All fish with *amhrII* mutations showed complete male to female sex reversal, as visualized by the formation of the ovarian cavity (OC) and the appearance of pre-vitellogenic oocytes when checked by gonad histology at 90 dah (Fig 6b). IHC analysis showed that Cyp19a1a was expressed like the control ovary, while Dmrt1 was not expressed in these sex-reversed XY gonads (Fig 6bA–6bD). The effect of AmhrII deficiency on gonadal differentiation could be detected from a very early stage. Cyp19a1a was expressed, while Dmrt1 was not expressed in AmhrII deficient XY gonads even at 10 and 30 dah (Fig 6bE–6bL). Consistently, knockout of *amhrII* in the XY fish resulted in increased serum E2 level compared with the control fish (Fig 6c). The genetic sex (XY) of these sex-reversed fish was further confirmed by PCR of a sex-linked marker (SPF/SPR) (Fig 6d).

## Discussion

In recent years, several different master SD genes have been identified in various fish species, giving the impression that the molecular mechanism underlying sex determination is different in each group. However, the limited data available for teleost SD genes suggest that members of the TGF-β superfamily (*gsdf* ^Y^, *amhy* and *amhrII*) could be part of a common pathway for sex determination in fish [11–13]. Tilapia sex determination has been widely studied with the goal of producing all-male progenies that have an enhanced growth rate in aquaculture. In Nile tilapia, some studies using fish from Egypt and Ghana have indicated that the main sex determining locus is on LG1 (with unresolved evidence regarding LG3) [35–37]. Recently, a sex-determining locus was mapped adjacent to the *amh* on LG23 in the Swansea strain of this species, using Simple Sequence Repeats (SSR) and sex specific markers [21, 24]. Thereafter, *amhy*, a Y-specific duplicate of the *amh* gene, was identified and suggested to be the candidate sex determiner [23]. However, this gene, named as *amhΔ-y* by us, is not responsible for sex determination in our strain. Another duplicated copy of *amh* with a missense SNP and a large fragment of promoter loss, which is located immediately downstream of *amhΔ-y* and designated as *amhy*, was found and demonstrated to be critical for male sex determination in our strain. Therefore, it is possible that different strain possesses different sex chromosome and different SD genes. Judging from the assembled sequence, it is reasonable to conclude that *amhy* arose from duplication of *amh* gene followed by 5608 bp promoter loss. *amhΔ-y* is an allele of the X-linked *amh* as most of the sequences in the upstream promoter of *amhΔ-y* were identical to the upstream of *amh* on the X chromosome.

*amhy* could be considered a candidate SD gene of the Nile tilapia because of its Y specific expression profile. Due to the high homology of three *amh*, four pairs of gonadal transcriptomes from different development stages were used to analyze their expression. It was found that *amhy* expression was restricted to XY testis, with expression at the beginning of critical sex determination period 5 dah, clearly preceding the first signs of morphological differentiation of ovaries and testes (~25 dah) [38]. According to the transcriptome data, both *amhy* and *amh* mRNA were detected in the XY gonads at 5 dah. Therefore, Western blot results indirectly suggested the expression of Amhy protein in XY gonads at 5 dah. In contrast, *amh* expression in XY gonads was much lower than that of XX gonads at 5 dah. Although the *amhΔ-y* was also found to be restricted to XY testis, we found there is a 5 bp insertion in exonVI of the *amhΔ-y* in addition to the previously reported TGF-β domain 233 bp deletion in exonVII [23]. It is this frameshift mutation that generates a truncated Amh lacking the TGF-β domain, which is important for binding to AmhrII. The truncated Amh could not directly bind to AmhrII even if the 233 bp sequence in exonVII was not deleted. Therefore, *amhΔ-y* might be a degenerated gene in tilapia.

The best way to understand the function of a gene in sex determination is to perform gain/loss of function studies and to characterize the resulting biological effects. For instance, overexpression of a 117 kb genomic DNA fragment that carries *dmy* in XX medaka, or the presence of a genomic fragment that included *gsdf* ^Y^, converts XX individuals into sex-reversed XX males [13, 39]. Knockdown of the Patagonian pejerrey *amhy* in XY fish led to an up-regulation of female factors and the development of ovaries [11]. In the present study, knockout of *amhy* or both *amhy* and *amhΔ-y* using CRISPR/Cas9 resulted in ovarian development in XY fish, while mutation of *amhΔ-y* alone could not. In contrast, overexpression from the *amhy* genomic region or its ORF under the control of CMV promoter induced testicular differentiation in XX fish, while overexpression of *amhΔ-y* alone in XX fish resulted in no sex reversal. Therefore, we demonstrated that *amhy* is necessary and sufficient to induce testicular differentiation in tilapia. A detailed analysis showed that the missense SNP and the large fragment promoter loss in *amhy* might contribute to sex determination in tilapia. Recent studies reported that a few regulatory or coding sequence mutations in other pre-existing and duplicated genes can generate new SD genes in fishes. For example, a few differences in the cis-regulatory region of *gsdf* ^Y^ and *sox3* ^Y^ contribute to male sex determination in two medaka species. A missense SNP in AmhrII is the only difference associated with phenotypic sex in *Takifugu rubripes*. In this study, overexpression of *amhy* ORF in XX fish led to sex reversal, while overexpression of *amh* in XX fish resulted in no sex reversal, implying that the missense SNP (C/T) in the coding sequence might contribute to male sex determination in tilapia. In another Nile tilapia strain, a missense SNP in exonVI of *amh* was also associated with autosomal and temperature dependent sex reversal [40]. These studies indicated that small variations in the coding sequence of *amh* might have taken over a critical role in tilapia sex determination. The important characteristic of a master SD gene is its tight linkage with the non-recombinant part of the heterochromosome. Right now, we do not know the size of the male-specific non-recombining region and how many genes are located in the region in tilapia. Therefore, *amhy* is considered as the candidate sex determining gene in this strain of the Nile tilapia.

In this report, an XY specific up-regulation was detected in the expression of *amhrII* in the gonads from 5 dah onwards, coincident with sex determination in tilapia. Higher levels of *amhrII* expression in testis have been consistently observed in four tilapia species and several other fishes [12, 28, 41]. Importantly, mutations in *amhrII* in medaka and *Takifugu rubripes* lead to male to female sex reversal [12, 29]. In the present study, knockout of *amhrII* in XY fish resulted in 100% male to female sex reversal in tilapia. However, as in medaka, but unlike in *Takifugu rubripes*, *amhrII* is an autosomal gene located on LG20 in Nile tilapia. It is well documented that AmhrII is the receptor for Amh in mammals [25]. However, the receptors for *amhy* and *amh* in fish have not been identified. Notably, the expression profiles of *amh* and *amhy* was similar to that of *amhrII* during tilapia gonadal development and these factors were found to be co-localized in cells surrounding the germ cells at 5 dah. Both Amhy and Amh have the identical TGF-β domain, which is responsible for binding to its receptor. According to these results, Amhy might be the ligand of AmhrII. Therefore, Amhy signal functions through AmhrII to determine sex determination in tilapia. Further investigations may reveal the mechanism by which Amhy/AmhrII signal pathway determines tilapia male sex. In addition, knockout of *amhrII* resulted in 100% sex reversal, while knockout of *amhy* in F_0_ fish XY only resulted in about 60% sex reversal. As the F_0_ fish were mosaic, the mutation rate varies individually. Therefore, only some of the F_0_ knockout fish displayed sex reversal. A thorough analysis of gene mutation of F_1_ knockout fish indicated that the ratio for fish bearing *amhy* frameshift mutation versus fish bearing frameshift mutation is approximately 60% (23/34), which is exactly the ratio of sex reversed fish to F_1_ positive fish. The probability for *amhy* and *amhΔ-y* mutation in different cell types of F_0_ fish should be the same as that of the germ cells. This explains why only 60% of the F_0_ positive fish displayed male to female sex reversal. In contrast, knockout of *amhrII* is equal to disrupt whole signal pathway, and therefore, resulted in 100% sex reversal.

*amhy* was first reported as the sex determiner in Patagonian pejerrey [11]. Existing evidence support the notion that the Y-specific duplication of Amh arose independently in tilapia and Patagonian pejerrey. This study provides a new example of convergent evolution for the formation of SD gene. Although there are reports showing sex determination roles for Amh and AmhrII in several species, our results are the first that demonstrate that both genes are critical for sex determination in a single species (Table 3). Even though *Amh* and *AmhrII* are not the master sex determination genes in mammals, chicken, and other fish species, they are also essential for testicular differentiation [3, 25, 28, 42, 43]. For example, loss-of-function mutants of AMH in the male mouse lead to partial hermaphroditism, with the uterus and oviduct present along with the testis, but no ovaries [25]. Our study highlights the significance of TGF-β signaling pathway in fish sex determination. Its role in sex determination and differentiation in other vertebrates deserves further investigation.

**Table 3:** Overview of the *amh*, *amhy*, *amhΔ-y* and *amhrII* in tilapia sex determination.

Genotype	Ligand			Receptor	Phenotype
	*amh*	*amhy*	*amhΔ-y*	*amhrII*	
**XY**	+	-	-	+	**Female**
**XY**	+	-	+	+	**Female**
XY	+	+	-	+	Male
**XX**	+	++	++	+	**Male**
**XX**	+	++	-	+	**Male**
XX	+	-	++	+	Female
XX	++	-	-	+	Female
XX	+/-	-	-	+	Female
**XY**	+	+	+	-	**Female**

Estrogen plays a critical role in ovarian differentiation and maintenance in a variety of vertebrates [44–48]. For instance, administration of estrogens can reverse phenotypic males to females in marsupials [49], birds [50], reptiles [51], and teleosts [52, 53]. Foxl2 is a key factor involved in female sex determination in vertebrates, including fishes and mammals [9, 34, 46, 54]. It is worth noting that Foxl2 directly activates the expression of *cyp19a1a*, encoding aromatase, a key enzyme responsible for estrogen production in tilapia [44], goat [55], mouse [54], and human cells [56]. A possible mechanism of Amh/AmhrII action in fish is through suppression of aromatase expression, decreasing estrogen levels so as to promote testis formation, as has been described in mammals and birds [57, 58]. Knockdown of Patagonian pejerrey *amhy* in XY fish resulted in up-regulation of *foxl2* and *cyp19a1a* expression [11]. Similar results were also obtained in *hotei* mutant medaka [29]. Consistent with these reports, knockout of *amhy* and *amhrII* in XY tilapia resulted in increased Cyp19a1a expression and serum E2 levels during sex reversal. On the other hand, *amhy*/*amh* transgenic overexpression of *amhy* in XX fish displayed no Cyp19a1a expression. Therefore, Amh/AmhrII signaling might play a critical role in male sex determination via regulation of the Foxl2-aromatase pathway in teleosts.

In conclusion, our results suggest that the tandem duplicated *amhy* is essential for male sex determination in the Nile tilapia. Mutation of *amhy* in XY fish resulted in male to female sex reversal and mutation of *amhΔ-y* could not, while overexpression of *amhy* in XX fish resulted in female to male sex reversal. Further, knockout of the *amh* type II receptor (*amhrII*) in XY fish also resulted in male to female sex reversal. Our findings highlight the significance of TGF-β signaling pathway in fish sex determination. Amhy/AmhrII play a critical role in the regulation of sex determination, probably via regulation of aromatase expression in teleosts. The role of this pathway in sex determination of other vertebrates deserves further investigation.

## Materials and Methods

### Ethics statement

Animal experiments were conducted in accordance with the regulations of the Guide for Care and Use of Laboratory Animals and were approved by the Committee of Laboratory Animal Experimentation at Southwest University.

### Fish

The founder strain of the Nile tilapia, which was first introduced from Egypt in Africa, was obtained from Prof. Nagahama (Laboratory of Reproductive Biology, National Institute for Basic Biology, Okazaki, Japan) and reared in large tanks with a circulating aerated freshwater system. All-XX and all-XY progenies were obtained by crossing sex-reversed XX pseudomales or YY supermales with normal females (XX).

### Screening of tilapia's fosmid library and sequence assembling

The microarrayed fosmid library of XY tilapia genomic DNA was constructed using the pCC2FOS vector (Epicentre, USA) according to the manufacturer's protocol [30]. X- and Y- specific clones were isolated by PCR screening of the library using a pair of sex specific primers (SPF: ATGGCTCCGAGACCTTGACTG; SPR: CAGAAATGTAGACGCCCAGGTAT) from marker-5 which amplified a 1422 bp fragment from the X chromosome and a 982 bp fragment from the Y chromosome [22]. DNA sequencing was carried out using Illumina HiSeq2000 technology by Invitrogen Corporation (Shanghai, China). The sequence was assembled by hand together with the local Blast software based on the following rationale: 1) the number of reads from the duplicated regions of Y156 were approximately twice of the un-duplicated region by Local Blast analysis with the sequence of X278 which is identical to the released genome sequences; 2) some reads from the Y156 fosmid displayed differential sequences (deletions and insertions) and SNPs; 3) some primers designed spanning the differential region can produce two fragments with the Y156 fosmid and YY genomic DNA while one band with X278 fosmid and XX genomic DNA, which can be used to help scaffold together the Illumina reads. To confirm the assembled sequences, twenty five PCR fragments were amplified and sequenced using 25 pair primers which were designed in the differential regions of *amhy* and *amhΔ-y*, as indicated in S15 Fig. The differences between the Y and X fosmid were further confirmed by PCR using XX, XY and YY fish genomic DNA as templates. Primers were listed in S4 Table.

### DNA, RNA extraction and cDNA synthesis

Tail fin was clipped from XX, XY and YY fish and incubated for 3 hrs at 55°C in lysis buffer containing 0.5% sodium dodecyl sulfate (SDS), 25 mM ethylenediaminetetraacetic acid (EDTA) (pH 8.0), 10 mM Tris-HCl (pH 8.0), and 200 μg/ml of proteinase K. The lysate was extracted with phenol/chloroform and precipitated with isopropanol. The extracted DNA was dissolved, quantified and then used as template for subsequent analyses.

Total RNA was extracted from various tissues (brain, pituitary, gill, heart, liver, spleen, intestine, ovary, testis, kidney, Muscle and head kidney) of pooled three XX and three XY fish at 180 dah using a column-based RNA extraction kit (Qiagen, Germany). After DNase I (RNase free) treatment, total RNA (500 ng) from each sample was reverse transcribed into first-strand cDNA using PrimeScript RT Master Mix Perfect Real Time Kit (Takara, Japan) according to the manufacturer's instructions. For each sample, in order to confirm that DNase I (RNase free) treatment of the RNA was complete, a negative control cDNA synthesis reaction without reverse transcriptase was performed.

### Tissue distribution analysis by RT-PCR

RT-PCR was performed to reveal the tissue distribution expression patterns of *amh*, *amhy*, *amhΔ-y*, and *amhrII*. Positive and negative controls were set up with plasmid DNA and negative control cDNA, respectively. *β-actin* was used as an internal control. Primer sequences used for RT-PCR were listed in S4 Table. The PCR conditions were as follows: after an initial denaturation at 94°C for 3 min, a 33-cycle reaction was carried out at 94°C for 30 s, 58°C for 30 s and 72°C for 30 s. For *β-actin* amplification, a 25-cycle reaction was used with an extension time of 45 s, other conditions being identical.

### Transcriptome analysis

Four pairs of XX and XY gonads from tilapia at 5, 30, 90 and 180 dah were sequenced using Illumina2000 HiSeq technology in our previous study [32]. The sequence with 5 bp (ATGTC) insertion in exonVI or the missense SNP (C/T) in exonII was used as query sequence (60 bp in length) to blast against the transcriptome clean reads using local BLAST software. *amh* and *amhy* were analyzed by counting reads with the SNP (C/T) in exonII. The expression of *amhΔ-y* was analyzed by blast in the transcriptome data with the *amhΔ-y* specific 5 bp (ATGTC) insertion in exonVI. A normalized measure of RPKM (reads per kb per million reads) was used to normalize the expression profiles of *amhy*, *amhΔ-y*, *amh* and *amhrII*. In addition, to get more precise expression data for *amhy* and *amh* in the 5 dah XY and XX gonads, two more pairs of gonadal transcriptomes from 5 dah fish (totally six gonadal transcriptomes, 3 from XX fish and 3 from XY fish), were sequenced to analyze *amhy* and *amh* expression.

### Production, characterization of antibody and western blot analysis

The production of Amh polyclonal antibody was performed as follows: The recombinant constructs of *amh* and *amhΔ-y* were prepared by cloning the ORFs of these genes into the pCold I vector (Takara, Japan). Recombinant Amh and AmhΔ-y were expressed and purified. The Amh was used as the antigen used to immunize rabbits for the production of polyclonal antibody. Ten days after the last immunization, rabbit serum was collected and recombinant protein purified by affinity chromatography on Sepharose 4B Fast Flow Resin (Sigma, Germany). Subsequently, the purified ployclonal antibody was evaluated by Western blotting. Briefly, total proteins extracted from XX and XY gonads from 5, 10, 30, 90, 180, 300 dah, YY gonads from 30 and 90 dah tilapia and the recombinant proteins (both purified and unpurified) were separated using 12% SDS-PAGE under reducing condition. Notably, the proteins used for 5 and 10 dah Western blot was extracted from the fish after removing head, tail, viscera and muscle. Separated proteins were transferred onto polyvinylidene fluoride (PVDF) membranes and then blocked with 5% fat milk and incubated with primary antibody of Amhy at 1:500 dilution, and then with a second antibody conjugated with horseradish peroxidase (Bio-Rad, USA) at 1:2000. Finally, the immunoreactive signals were detected with BeyoECL Plus Kit (Beyotime, China) and visualized on Fusion FX7 (Vilber Lourmat, France).

### Immunohistochemistry (IHC)

Gonads of 5-, 30-, 90- and 180- dah fish were dissected, fixed in Bouin's solution for 24 hrs at room temperature, and subsequently dehydrated, embedded in paraffin and serially sectioned at 5 μm thickness. IHC was performed to determine the cellular localization of Amh/AmhΔ-y/Amhy and AmhrII in gonads using the Amh and AmhrII antibodies at 1:500, 1:1000 dilution respectively. IHC staining was carried out as follows: After washing with PBS, the sections were treated in blocking solution, incubated with the primary rabbit polyclonal antibodies overnight at 4°C. Subsequently, the sections were incubated with a second antibody conjugated with horseradish peroxidase (Bio-Rad, USA) at 1:2000. Immunoreactive signals were visualized using diaminobenzidine (Sigma, USA) as substrate. Sections were counterstained with hematoxylin.

### gRNA design and Cas9 mRNA *in vitro* transcription

Two gRNA target sites were selected for each gene on the sense or antisense strand of *amh*/*amhy*, *amhrII* and *gsdf* with ZIFIT Targeter. BLAST with the tilapia genome was performed to avoid off-targets according to the principles reported previously [59]. In addition, a restriction enzyme cutting site adjacent to the NGG (PAM) was included for convenient mutagenesis analysis. The DNA template preparation, PCR conditions, gRNA *in vitro* transcription, gRNA purification, Cas9 mRNA *in vitro* transcription and purification were carried out as described previously [34]. *In vitro* transcription was performed with the Megascript T7 Kit (Ambion, USA) for 4 hrs at 37°C using 500 ng purified DNA as templates. The Cas9 plasmids were linearized with *Xba* I and purified by ethanol precipitation as templates. Cas9 mRNA was produced by *in vitro* transcription of 1 μg linearized template DNA with a T7 mMESSAGE mMACHINE Kit (Ambion, USA) according to the manufacturer’s instructions.

### Knockout of *amhy* and *amhrII* in tilapia

About 500 pg of the gRNA (150 ng/μl) and Cas9 mRNA (500 ng/μl), mixed at a molar ratio of 1:1, was microinjected directly into XX or XY fertilized eggs. Mutated fish were identified by loss of the restriction enzyme site. Mutation efficiencies and sequences of the mutated targets were evaluated by restriction enzyme digestion and Sanger sequencing as follows: the DNA fragments spanning the target for each fish were amplified. The recovered PCR products were purified and digested by restriction enzyme within the target. The uncleaved bands were recovered, sequenced and then aligned with the wild type. In addition, the percentage of uncleaved band (i.e., potential mutations in target site) was measured by quantifying the band intensity of the restriction enzyme digestion with Quantity One Software (Bio-Rad, USA). The indel frequency was calculated by dividing uncleaved band intensity to the total band intensity.

All the procedures for RNA and protein extraction from 90 dah control (n = 5) and F_0_ fish gonads (n = 5), cDNA synthesis, RT-PCR and Western blot were performed as described above. Two forward primers were designed in the target sites, together with reverse primers in another exon, for detection of *amh/amhΔ-y/amhy* mRNA expression in mutated gonads by RT-PCR. The primers were listed in S4 Table.

### Transgenic overexpression of Y156 fosmid, Amhy or AmhΔ-y ORF in XX fish

*Not* I linearized fosmid clone containing *amhy* (Y156) was injected into all XX fertilized eggs of tilapia. To detect *amhΔ-y* and *amhy* mRNA expression in transgenic fish, total RNA was extracted from the whole bodies of XX fry after removal of the yolk at 3 and 5 dah. All the procedures for RNA extraction, cDNA synthesis, and RT-PCR using Y specific primers (*amhΔ-y*-F1/R1) were carried out as described above. The detection of *amhΔ-y* expression was indirectly stated the successful overexpression of *amhy*. PCR was performed with Y specific primers (*amhΔ-y*-F1/R1) to detect the transgene in extracted genomic DNA (S4 Table).

Overexpression of Amhy or AmhΔ-y alone in XX fish was performed as follows: the *amhy* or *amhΔ-y* ORF was subcloned into the multiple cloning sites downstream of the cytomegalovirus (CMV) promoter of the pIRES-hrGFP-1a vector. Transgenic overexpression was carried out by injection of these constructs into the blastodisc of fertilized eggs of XX population. Genomic DNA extraction and positive fish screening were performed as described previous study [44]. Additionally, the transgenic fish gonads were subjected to histological and IHC assays to examine the effects of Amhy overexpression on sex determination.

### Phenotype, histological and IHC analyses of F_0_ knockout and overexpression fish

Gonads of *amh*/*amhΔ-y*/*amhy*, or *amhrII* knockout and control fish were dissected at 10, 30 and 90 dah. After fixation in Bouin's solution for 24 hours at room temperature, they were dehydrated and embedded in paraffin. Tissue blocks were sectioned at 5 μm and stained with hematoxylin and eosin for histological analysis or used for IHC. IHC using Dmrt1 and Cyp19a1a antibodies, which were diluted at 1:100, and 1:2500 respectively, was performed. Additionally, the gonads of fish overexpressing Y156 fosmid or *amhy* were subjected to histological and IHC assays at 90 dah after injection. The genotypes of all sex reversed fish were confirmed by a pair of sex specific primer (SPF/SPR) (S4 Table).

### Estrogen assay

Blood samples were collected from the caudal veins of the 3-month-old knockout (F_0_) as well as control fish. Serum estradiol-17β (E2) levels were measured using the E2 enzyme immunoassay kits (Cayman, USA). Sample purification and assays were performed according to the manufacturer's instructions.

### Production and gonadal phenotype analysis of the F_1_ XY fish

The *amh*/*amhΔ-y*/*amhy* mutant XY fish (target 1 and 2) with the moderate indel frequency were randomly selected as F_0_ founders. They were raised to sexual maturity and mated with wild-type XX tilapia to produce F_1_ fish. At 90 dah, the genomic DNA from F_1_ fish was extracted individually for genotyping and mutation assays. The genotype of all F_1_ fish were determined by a pair of sex specific primer (SPF/SPR). For F_1_ XY individual mutation assays, one pair of gene specific primers was designed to ensure specific amplification of *amhy* and *amhΔ-y* respectively in the first round of PCR. Then, the first round PCR products were diluted and used as template for the second round of PCR using the knockout fish screening primers (S4 Table). Restriction enzyme *BstN* I digestion of the amplified fragments from second PCR and Sanger sequencing were performed to confirm mutation types of F_1_ fish. The SNPs near the two targets were used to distinguish *amhΔ-y* and *amhy*. The fish were processed for histological analysis to analyze their gonadal phenotype.

## Supporting Information

S1 Fig Fosmid library construction and PCR screening of fosmid clones containing the sex specific fragments. **A**, A schematic illustration of colony storage and pool construction. The fosmid library was arrayed in three hundred 384-well plates. Column-, row- and plate-pools were constructed for each plate. In total, 25 super-pools were made, each covering twelve 384-well plates. Any gene of interest can be screened only by 3 rounds of PCR (minimally 77 (25+12+40) PCR reactions: 25 super-pools, 12 plate-pools, 40 row- and column-pools). **B**, Gel electrophoresis of fosmid clones digested by *Not* I. To check the insert size, 10 clones were randomly selected from the library. The plasmids of these clones were prepared, digested with *Not* I and visualized by electrophoresis. All 10 clones contained insert with an average size of around 40 kb, ranging from 38 to 42 kb. **C**, PCR screening of the library was performed using a pair of sex specific primer (SPF: ATGGCTCCGAGACCTTGACTG; SPR: CAGAAATGTAGACGCCCAGGTAT). The super-, plate-, row- and column-pools were identified to locate the positive clone. An X specific and a Y specific fosmid clone, designated as X278 and Y156, which produced a band of 1422 and 982 bp, respectively, were identified in the XY tilapia fosmid library. M, DNA molecular standard.
(TIF)

S2 Fig Demonstration of the 5 bp insertion of *amhΔ-y* in the Nile tilapia. **A**. Sanger sequencing showed the 5 bp insertion was only present in *amhΔ-y*, but not in *amh* and *amhy*. A Taq^α^ I (TCGA) restriction site overlapped the 5 bp insertion. **B**. Identification of the existing 5 bp insertion in *amhΔ-y* by restriction enzyme digestion. The results showed that cleaved two bands were found in the XY and YY genomic DNA, not in XX genomic DNA.
(TIF)

S3 Fig Verification of six differences in the gene sequence of *amh*, *amhΔ-y* and *amhy*. **A**, X-specific amplification for Ins1; **B**, Y-specific amplification for Ins1; **C**, X-specific amplification for Del1; **D**, Y-specific amplification for Del1; **E**, X-specific amplification for Del2; **F**, Y-specific amplification for Del2; **G**, Y-specific amplification for Del3; **H**, Y-specific amplification for Ins2; **I**, Y-specific amplification for Del4; **J**, Demonstration of the deletion of 5608 bp (Del5) in the upstream of the *amhy*. A forward primer before the 5608 bp deletion and a reverse primer located in the exonVI of *amhy* without the 5 bp insertion were designed to demonstrate the 5608 bp deletion. As expected, two fragments of 2414 bp and 8022 bp were amplified in the XY genomic DNA, while one fragment of 2414 bp was amplified in the Y156 fosmid and YY genomic DNA and one fragment of 8022 bp was amplified in the X278 fosmid and XX genomic DNA. Y156, plasmid of Y-specific positive fosmid clone; X278, plasmid of X-specific positive fosmid clone; -, negative control; M, DNA molecular standard. Primers used in this experiment are listed in S4 Table.
(TIF)

S4 Fig Alignments of *amhy*, *amh* and *amhΔ-y* ORFs and their encoded amino acid sequences. The coding sequence of *amhy* was identical to the *amh* except a single nucleotide polymorphisms (SNP) (C/T) in exonII, which changes an amino acid (Ser/Leu92) in the N-terminal region. AmhΔ-y has an open reading frame (ORF) of 777 bp encoding a putative protein of 258 aa (amino acid) without the TGF-β domain. The Amh/Amhy has an ORF of 1,545 bp, encoding a putative protein of 514 aa with the TGF-β domain.
(TIF)

S5 Fig The tissue distribution analysis of *amhΔ-y*, *amhy/amh* and *amhrII* in tilapia gonads by RT-PCR. *amh/amhy* and *amhrII* were expressed exclusively in gonads, with greater expression in testis. *amhΔ-y* was expressed only in the XY testis, not in the XX ovary. Tissues from 3 XX and 3 XY fish at 180 dah were pooled for the experiment. B, brain; P, pituitary; G, gill; H, heart; S, spleen; L, liver; I, intestine; O, ovary; K, kidney; Mu, muscle; T, testis; HK, head kidney; M, DNA molecular standard; +, positive control; -, negative control. *β-actin* as the internal control.
(TIF)

S6 Fig The expression profile of *amhrII* in XX and XY gonads of tilapia at 5, 30, 90 and 180 dah. *amhrII* were observed in both XX and XY gonads, with significantly higher expression in XX than in XY gonads at 5 dah, while it showed higher expression in XY than in XX gonads at 30 dah onwards. A normalized measure of RPKM (reads per kb per million reads) was used to normalize the expression of *amhrII*. The numbers over the bars indicate RPKM.
(TIF)

S7 Fig Characterization of Amh antibody and expression of Amh, AmhΔ-y and Amhy in gonads by western blot. The recombinant constructs of *amh* and *amhΔ-y* were prepared by cloning the ORF into the pCold I vector. The specificity of the Amh antibody was confirmed by Western blotting. The blots revealed specific bands of ~54 kDa, corresponding to the calculated molecular weight of Amh/Amhy, in the protein samples extracted from the XX ovary, XY and YY testis. Another band of ~27 kDa, corresponding to AmhΔ-y, was detected exclusively in the protein samples extracted from the XY and YY testis. Amh/Amhy was detected in XY gonads of 5, 10, 30, 90, 180 and 300 dah tilapia, and also with expression of Amh in XX tilapia at these stages, while AmhΔ-y was only detected in 5, 10, 30 and 90 dah XY gonads. In addition, Amh and Amhy were also detected in the proteins from the YY gonads of 30 and 90 dah tilapia. Gapdh was used as the internal control. Lane 1, molecular weight markers (kDa); lanes 2 and 5, uninduced; lanes 3 and 6, IPTG induced; lanes 4 and 7, purified His-Amh and -Amhy recombinant protein respectively; lanes 8, 10, 12, 14, 16,18, proteins extracted from XY testis at 5, 10, 30, 90, 180 and 300 dah; lanes 9, 11, 13, 15, 17, 19, proteins extracted from XX ovary at 5, 10, 30, 90, 180 and 300 dah; lanes 20, 21, proteins extracted from YY testis at 30 and 90 dah.
(TIF)

S8 Fig Cell localization of Amh/AmhΔ-y/Amhy and AmhrII in gonads by immunohistochemistry. The Amh/AmhΔ-y/Amhy and AmhrII proteins were located in somatic cells surrounding germ cells in the XY gonads at 5 dah (**A**, **E**), at later stages Amh/AmhΔ-y/Amhy in myoid cells and Sertoli cell (**B**, **C**, **D**), while AmhrII was observed in spermatogonia and Sertoli cells of the testis at 30, 90 and 180 dah (**F**, **G**, **H**). dah, days after hatching. Scale bars, 15μm (B, C, D, F, G, H); 10μm (A, E).
(TIF)

S9 Fig The CRISPR system disrupt *amhy* and *amhΔ-y* on the Y chromosome equally. Two pairs of primers were designed to amplified *amhy* and *amhΔ-y* on the Y chromosome specifically in the genome of F_0_ knockout XY fish. The genomic DNA fragments spanning the target for each fish were amplified from pooled genomic DNA from up to about 20 embryos. Restriction enzyme digestion with *BstN* I and *BsrB* I showed that the CRISPR system disrupt both genes on the Y chromosome equally.
(TIF)

S10 Fig RT-PCR and Western blot analysis of *amh*/*amhΔ-y*/*amhy* expression in knockout and control fish. Schematic drawing of primer pairs used for detecting mutagenesis in cDNA by RT-PCR. A forward primer was designed in the target sequence. RT-PCR analysis using cDNA from 90 dah mutated gonads as template demonstrated that indels from gRNA/Cas9 resulted in less amplification of the expected *amh*/*amhΔ-y*/*amhy* mRNA fragments and gave weak bands in agarose gel, compared with the control (**A**). *β-actin* was used an internal control. Consistently, Western blot analysis showed that no AmhΔ-y and decreased Amh/Amhy protein level was detected in the gonads of F_0_ knockout fish by Western blot (**B**).
(TIF)

S11 Fig Genotype of the sex-reversed XY fish was confirmed by sex-specific marker (SPF/SPR). 1–13, sex-reversed XY fish with *amh*/*amhΔ-y*/*amhy* knockout. M, DNA molecular standard.
(TIF)

S12 Fig Knockout of *amh* in XX resulted in blockage of oogenesis. A number of oogonia and few oocytes were observed in F_0_ Amh knockout XX gonads at 90 dah (**A**), while all stages of oogenic cells were observed in the control ovary (**B**). Scale bars, 10μm (A); 50μm (B).
(TIF)

S13 Fig Amino acid alignments of CRISPR/Cas9-induced *amhΔ-y* and *amhy* mutant alleles in F_1_ XY fish. A frame shift occurs in the target 1 and 2 before the TGF-β domain to cause premature terminations.
(TIF)

S14 Fig No sex reversal was detected after overexpression of Amh and AmhΔ-y in XX fish. **A**, **D**, Screening of transgenic fish by RT-PCR using the GFP specific primers. P, positive control using the plasmid containing the GFP; N, Negative control using water. **B**, **E**, Overexpression of Amh or AmhΔ-y in XX fish resulted in no sex reversal, compared with the control XX fish (**C**, **F**).
(TIF)

S15 Fig Confirmation of the assembled sequence by gene specific primers. Twenty five pairs of gene specific primers were designed in the differential regions of *amhy* and *amhΔ-y* to amplify fragments with overlapping ends from the Y156 fosmid.
(TIF)

S1 Table Test of sex specific primers from the *amhΔ-y* region in genotyping progeny from XX (♀) × XY (♂) crosses in Nile tilapia. (DOC)

S2 Table *amhΔ-y* is close to neutral evolution in comparison to *amhy*. (DOC)

S3 Table Mutation rates of *amh*/*amhΔ-y*/*amhy* and *amhrII* genes induced by CRISPR/Cas9. (DOC)

S4 Table Primers used in this study. (DOC)

S1 Sequence Sequences of tilapia X-linked *amh*, *amhΔ-y* and *amhy*. (DOC)
