# Supplementary figures and images for: A Tandem Duplicate of Anti-Müllerian Hormone with a Missense SNP on the Y Chromosome Is Essential for Male Sex Determination in Nile Tilapia, Oreochromis niloticus
Source: PLoS Genet. 2015 Nov 20;11(11):e1005678. doi: 10.1371/journal.pgen.1005678 (PMC4654491; doi:10.1371/journal.pgen.1005678)

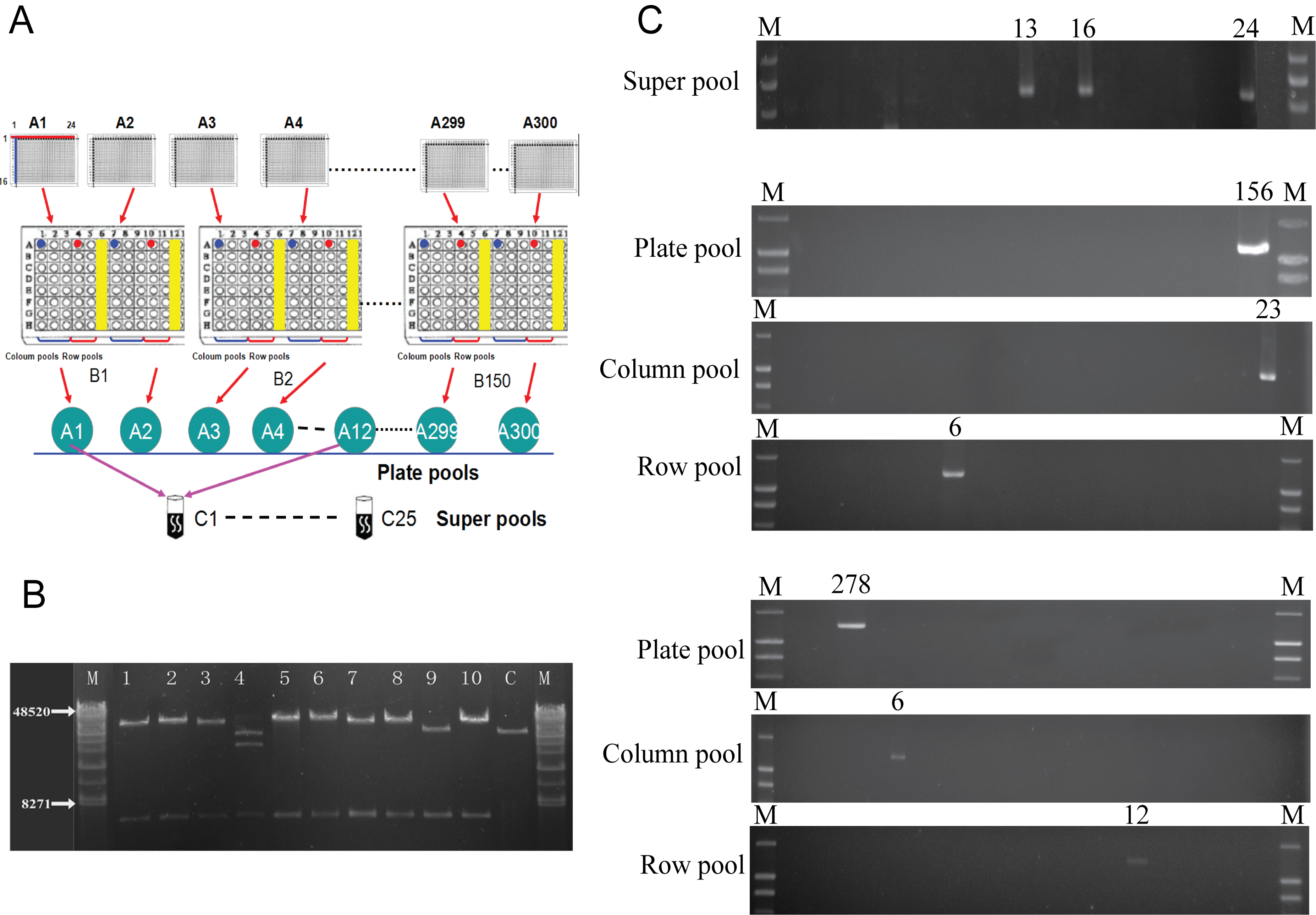

Supplement: S1 Fig — A, A schematic illustration of colony storage and pool construction. The fosmid library was arrayed in three hundred 384-well plates. Column-, row- and plate-pools were constructed for each plate. In total, 25 super-pools were made, each covering twelve 384-well plates. Any gene of interest can be screened only by 3 rounds of PCR (minimally 77 (25+12+40) PCR reactions: 25 super-pools, 12 plate-pools, 40 row- and column-pools). B, Gel electrophoresis of fosmid clones digested by Not I. To check the insert size, 10 clones were randomly selected from the library. The plasmids of these clones were prepared, digested with Not I and visualized by electrophoresis. All 10 clones contained insert with an average size of around 40 kb, ranging from 38 to 42 kb. C, PCR screening of the library was performed using a pair of sex specific primer (SPF: ATGGCTCCGAGACCTTGACTG; SPR: CAGAAATGTAGACGCCCAGGTAT). The super-, plate-, row- and column-pools were identified to locate the positive clone. An X specific and a Y specific fosmid clone, designated as X278 and Y156, which produced a band of 1422 and 982 bp, respectively, were identified in the XY tilapia fosmid library. M, DNA molecular standard. (TIF) [file pgen.1005678.s001.tif]

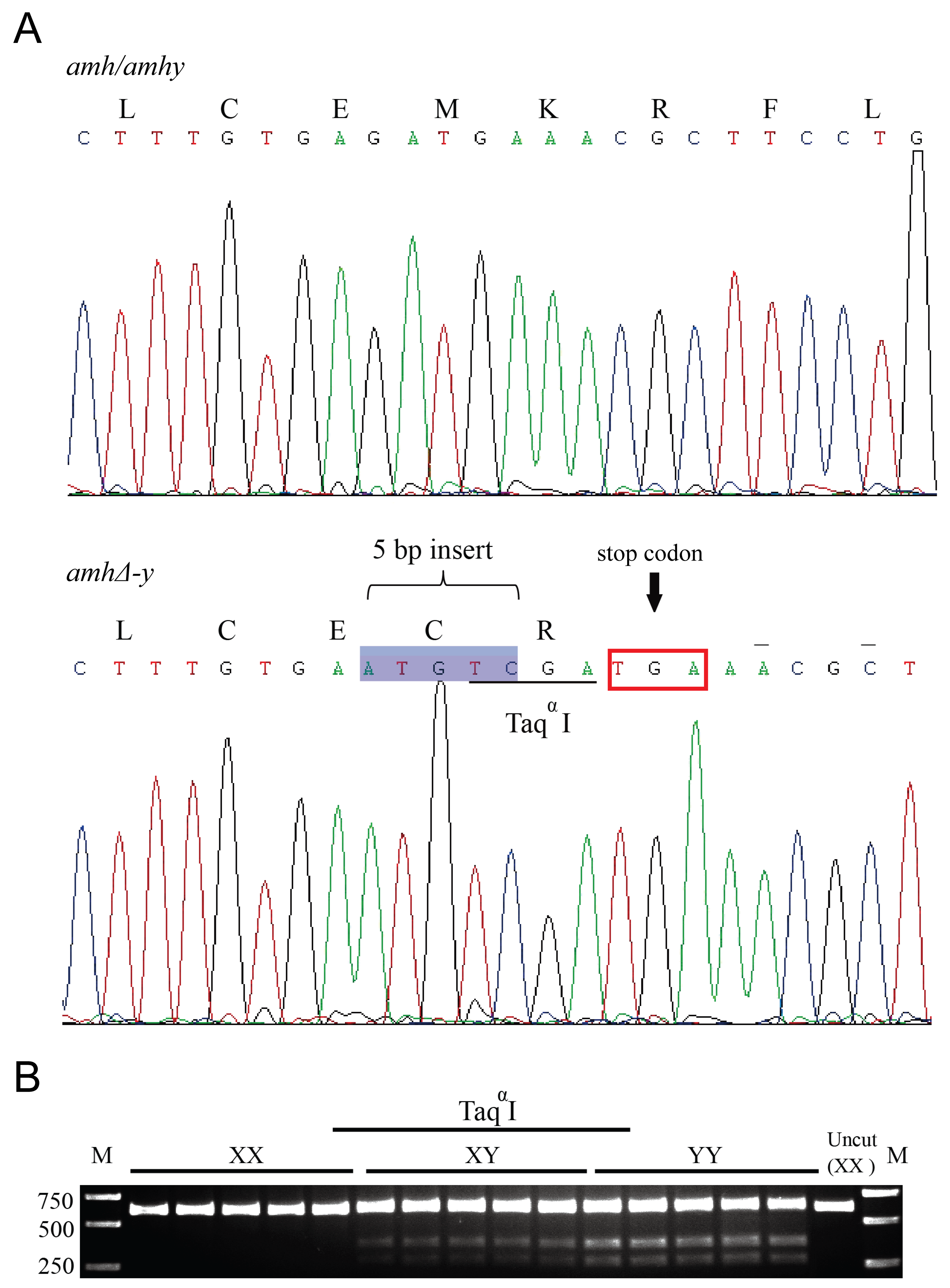

Supplement: S2 Fig — A. Sanger sequencing showed the 5 bp insertion was only present in amhΔ-y, but not in amh and amhy. A Taqα I (TCGA) restriction site overlapped the 5 bp insertion. B. Identification of the existing 5 bp insertion in amhΔ-y by restriction enzyme digestion. The results showed that cleaved two bands were found in the XY and YY genomic DNA, not in XX genomic DNA. (TIF) [file pgen.1005678.s002.tif]

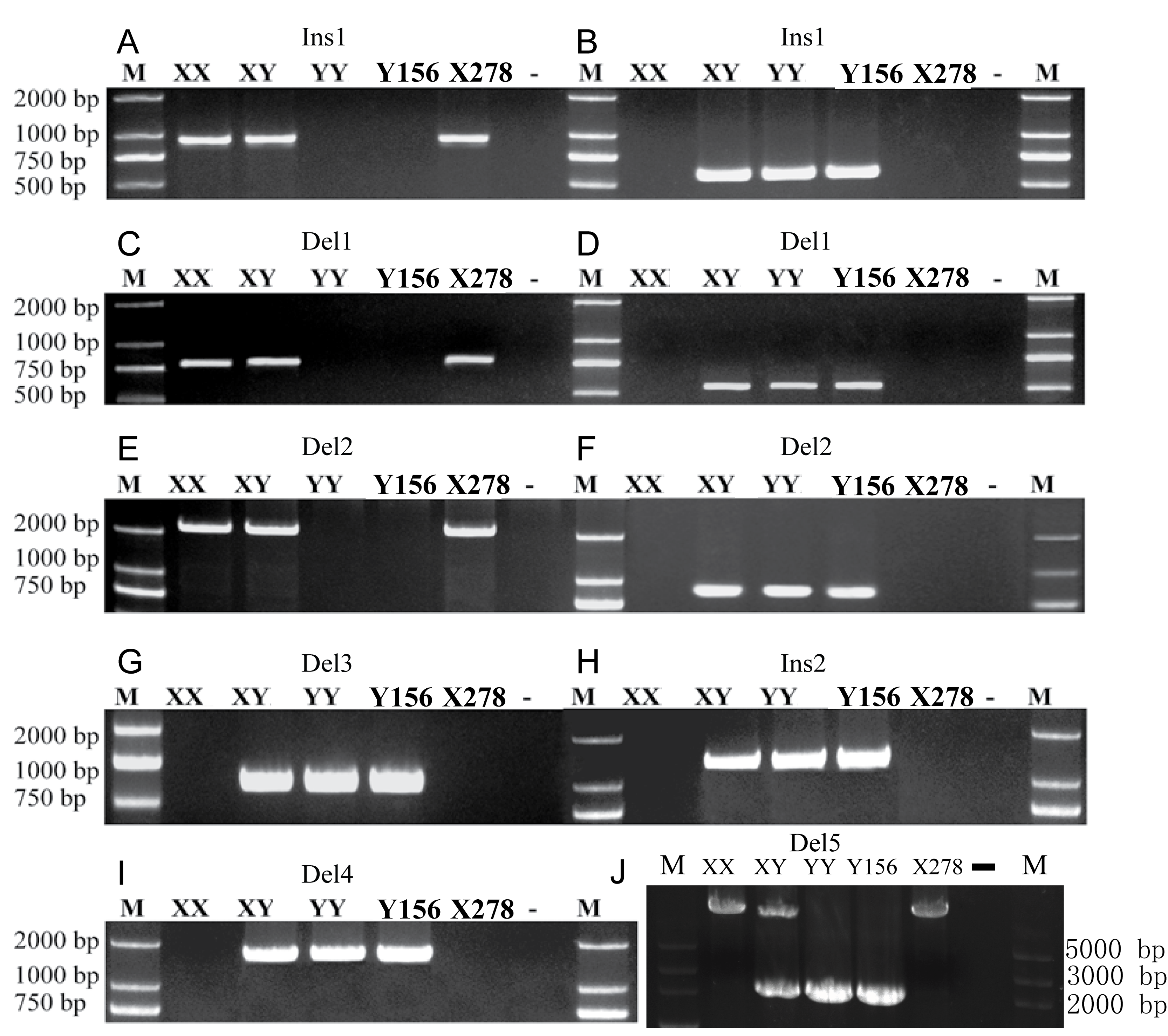

Supplement: S3 Fig — A, X-specific amplification for Ins1; B, Y-specific amplification for Ins1; C, X-specific amplification for Del1; D, Y-specific amplification for Del1; E, X-specific amplification for Del2; F, Y-specific amplification for Del2; G, Y-specific amplification for Del3; H, Y-specific amplification for Ins2; I, Y-specific amplification for Del4; J, Demonstration of the deletion of 5608 bp (Del5) in the upstream of the amhy. A forward primer before the 5608 bp deletion and a reverse primer located in the exonVI of amhy without the 5 bp insertion were designed to demonstrate the 5608 bp deletion. As expected, two fragments of 2414 bp and 8022 bp were amplified in the XY genomic DNA, while one fragment of 2414 bp was amplified in the Y156 fosmid and YY genomic DNA and one fragment of 8022 bp was amplified in the X278 fosmid and XX genomic DNA. Y156, plasmid of Y-specific positive fosmid clone; X278, plasmid of X-specific positive fosmid clone; -, negative control; M, DNA molecular standard. Primers used in this experiment are listed in S4 Table. (TIF) [file pgen.1005678.s003.tif]

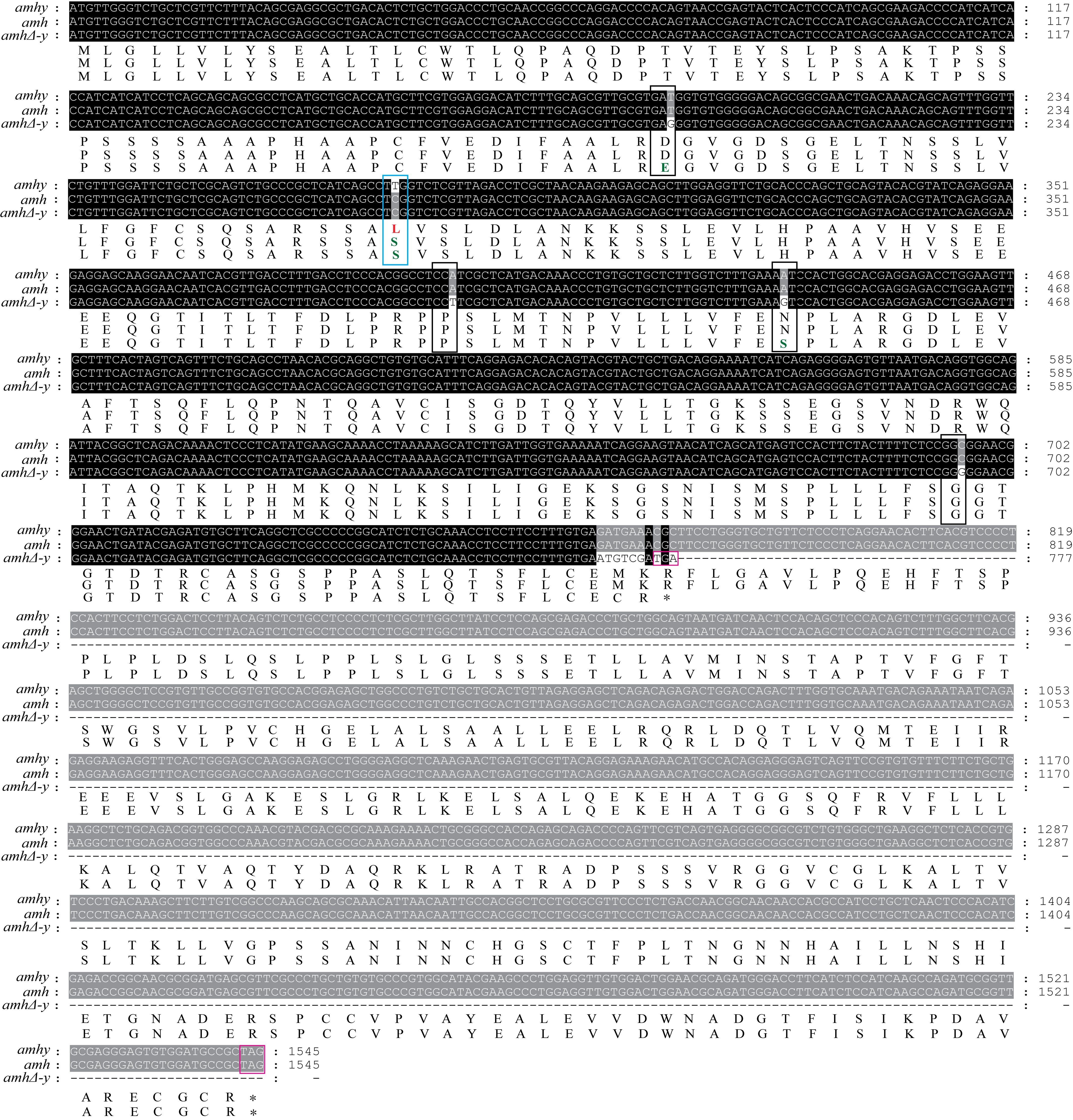

Supplement: S4 Fig — The coding sequence of amhy was identical to the amh except a single nucleotide polymorphisms (SNP) (C/T) in exonII, which changes an amino acid (Ser/Leu92) in the N-terminal region. AmhΔ-y has an open reading frame (ORF) of 777 bp encoding a putative protein of 258 aa (amino acid) without the TGF-β domain. The Amh/Amhy has an ORF of 1,545 bp, encoding a putative protein of 514 aa with the TGF-β domain. (TIF) [file pgen.1005678.s004.tif]

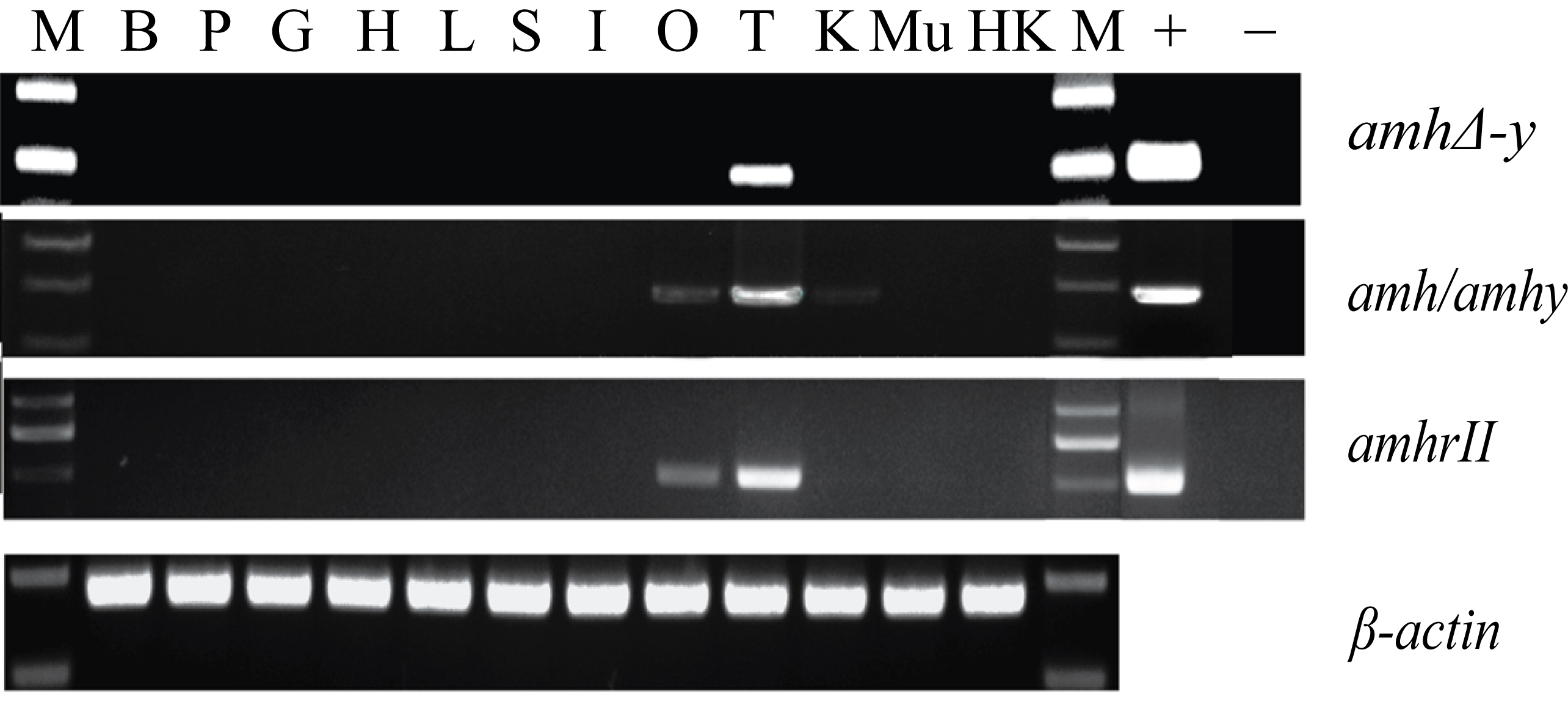

Supplement: S5 Fig — amh/amhy and amhrII were expressed exclusively in gonads, with greater expression in testis. amhΔ-y was expressed only in the XY testis, not in the XX ovary. Tissues from 3 XX and 3 XY fish at 180 dah were pooled for the experiment. B, brain; P, pituitary; G, gill; H, heart; S, spleen; L, liver; I, intestine; O, ovary; K, kidney; Mu, muscle; T, testis; HK, head kidney; M, DNA molecular standard; +, positive control; -, negative control. β-actin as the internal control. (TIF) [file pgen.1005678.s005.tif]

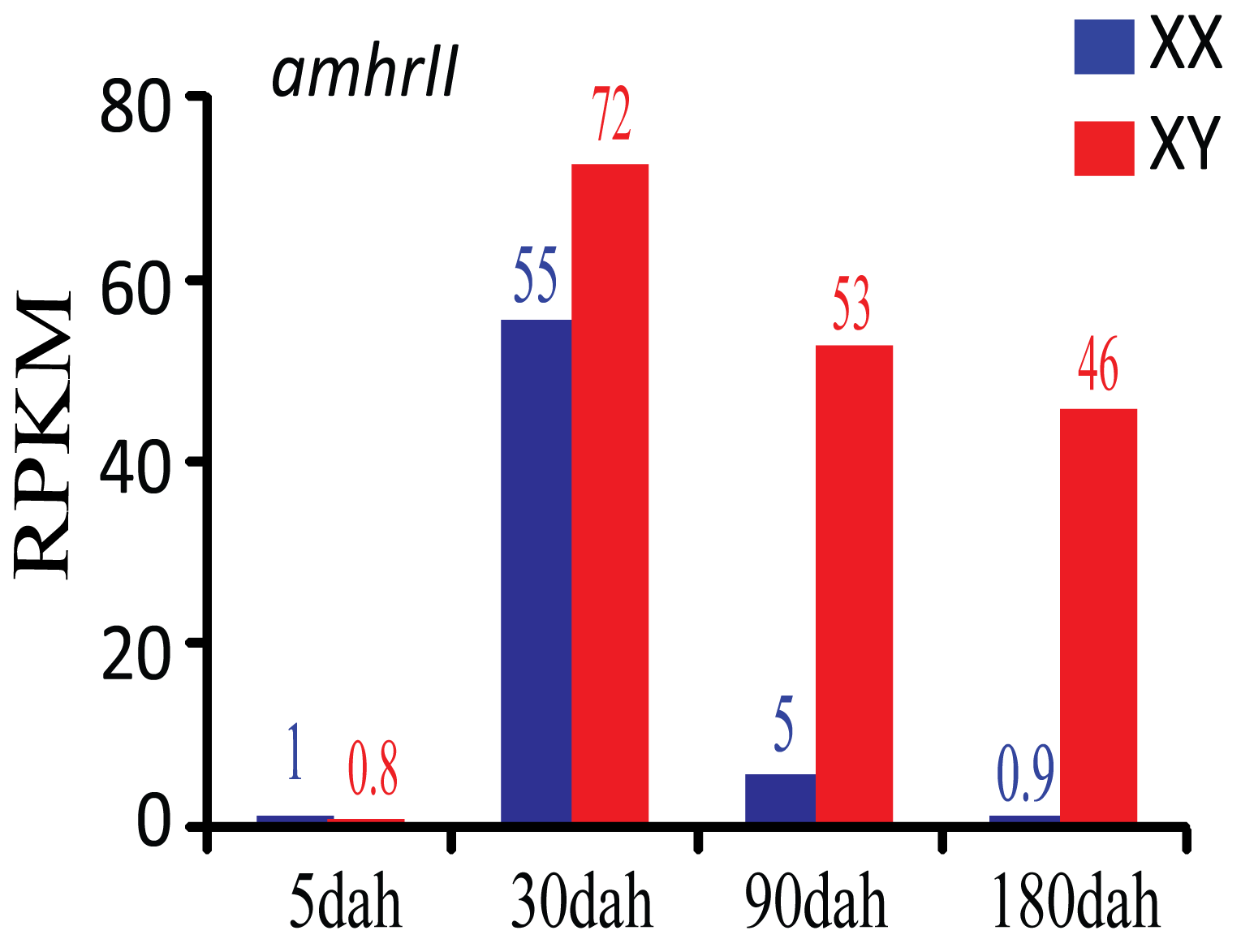

Supplement: S6 Fig — amhrII were observed in both XX and XY gonads, with significantly higher expression in XX than in XY gonads at 5 dah, while it showed higher expression in XY than in XX gonads at 30 dah onwards. A normalized measure of RPKM (reads per kb per million reads) was used to normalize the expression of amhrII. The numbers over the bars indicate RPKM. (TIF) [file pgen.1005678.s006.tif]

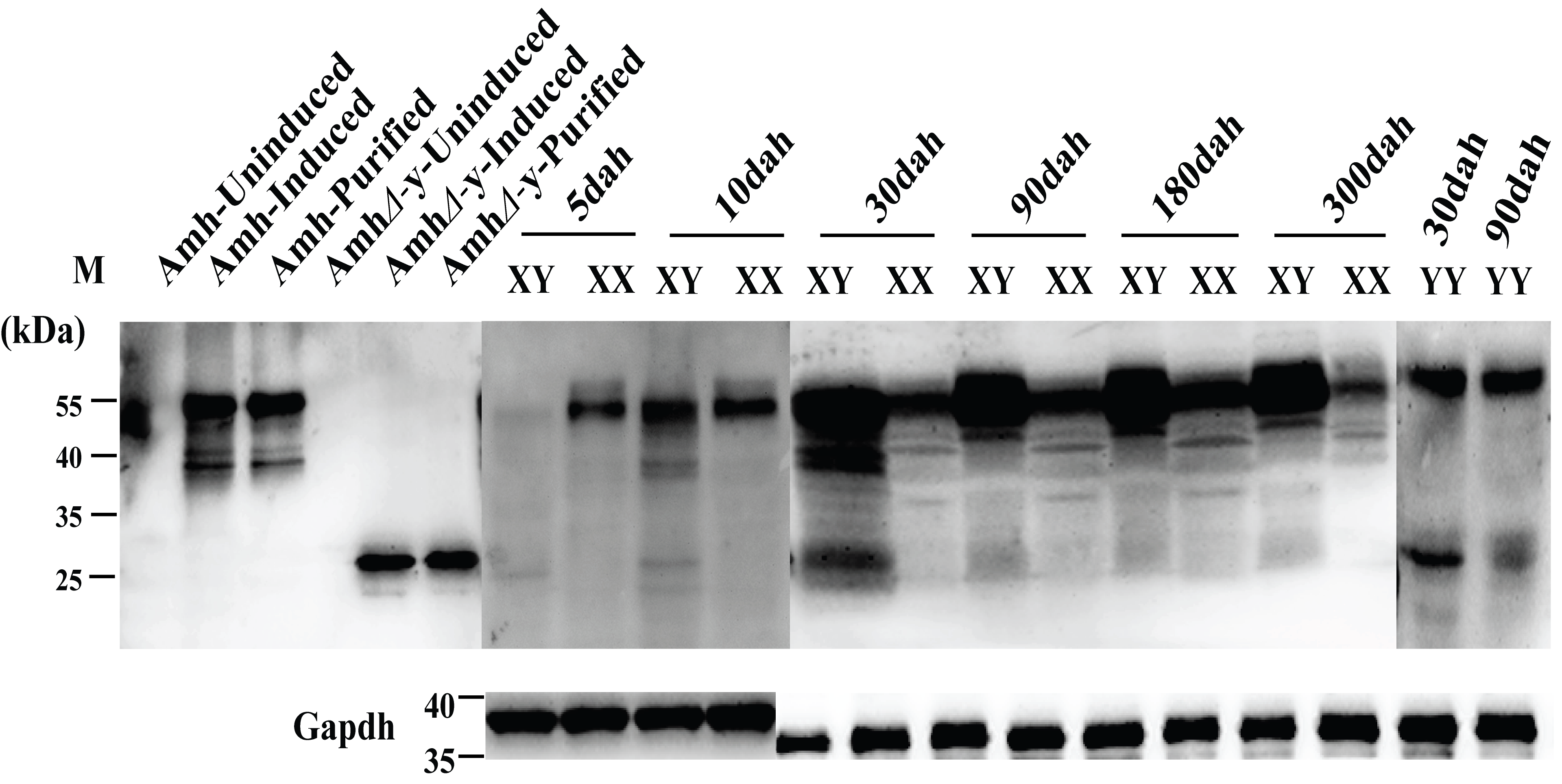

Supplement: S7 Fig — The recombinant constructs of amh and amhΔ-y were prepared by cloning the ORF into the pCold I vector. The specificity of the Amh antibody was confirmed by Western blotting. The blots revealed specific bands of ~54 kDa, corresponding to the calculated molecular weight of Amh/Amhy, in the protein samples extracted from the XX ovary, XY and YY testis. Another band of ~27 kDa, corresponding to AmhΔ-y, was detected exclusively in the protein samples extracted from the XY and YY testis. Amh/Amhy was detected in XY gonads of 5, 10, 30, 90, 180 and 300 dah tilapia, and also with expression of Amh in XX tilapia at these stages, while AmhΔ-y was only detected in 5, 10, 30 and 90 dah XY gonads. In addition, Amh and Amhy were also detected in the proteins from the YY gonads of 30 and 90 dah tilapia. Gapdh was used as the internal control. Lane 1, molecular weight markers (kDa); lanes 2 and 5, uninduced; lanes 3 and 6, IPTG induced; lanes 4 and 7, purified His-Amh and -Amhy recombinant protein respectively; lanes 8, 10, 12, 14, 16,18, proteins extracted from XY testis at 5, 10, 30, 90, 180 and 300 dah; lanes 9, 11, 13, 15, 17, 19, proteins extracted from XX ovary at 5, 10, 30, 90, 180 and 300 dah; lanes 20, 21, proteins extracted from YY testis at 30 and 90 dah. (TIF) [file pgen.1005678.s007.tif]

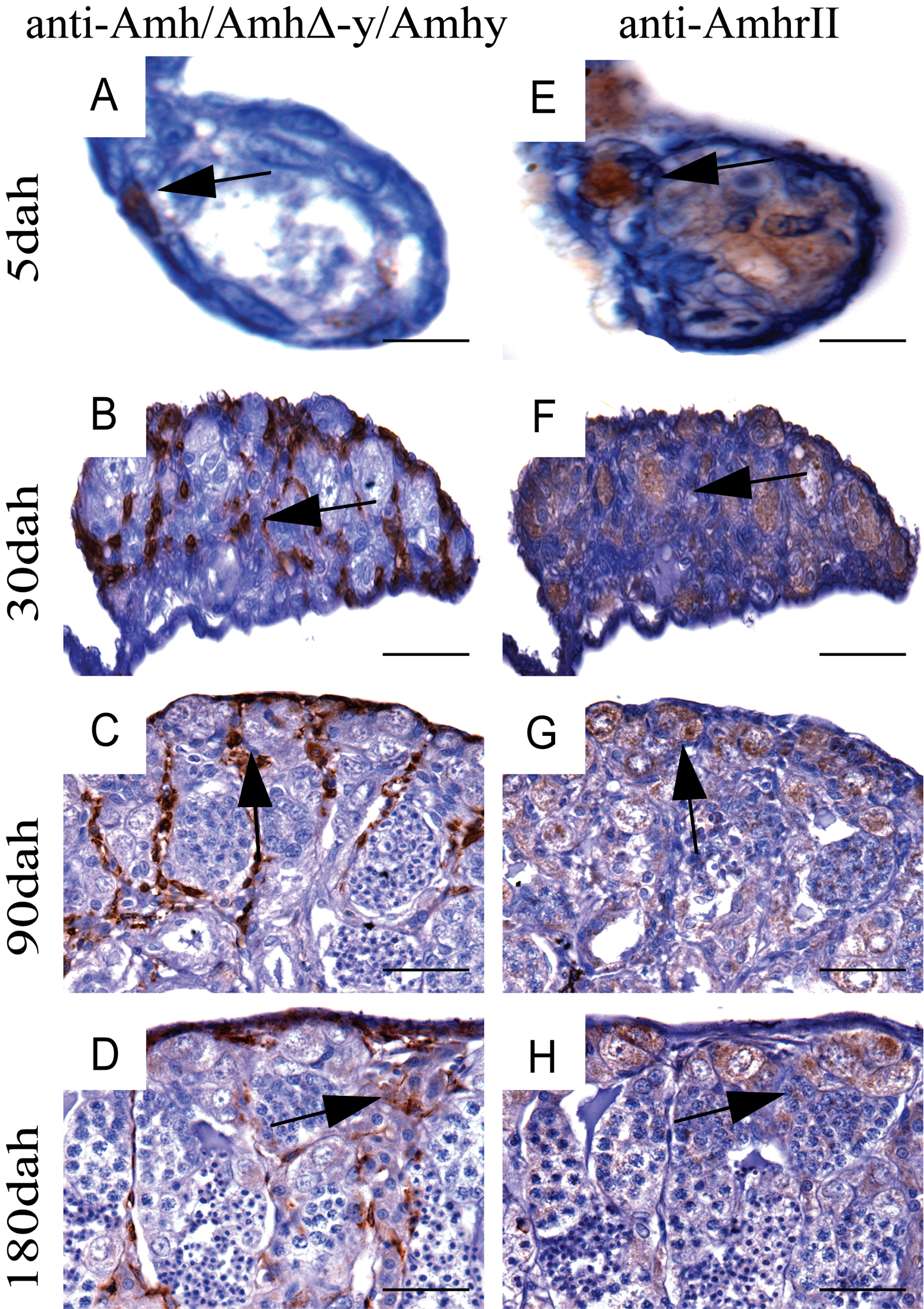

Supplement: S8 Fig — The Amh/AmhΔ-y/Amhy and AmhrII proteins were located in somatic cells surrounding germ cells in the XY gonads at 5 dah (A, E), at later stages Amh/AmhΔ-y/Amhy in myoid cells and Sertoli cell (B, C, D), while AmhrII was observed in spermatogonia and Sertoli cells of the testis at 30, 90 and 180 dah (F, G, H). dah, days after hatching. Scale bars, 15μm (B, C, D, F, G, H); 10μm (A, E). (TIF) [file pgen.1005678.s008.tif]

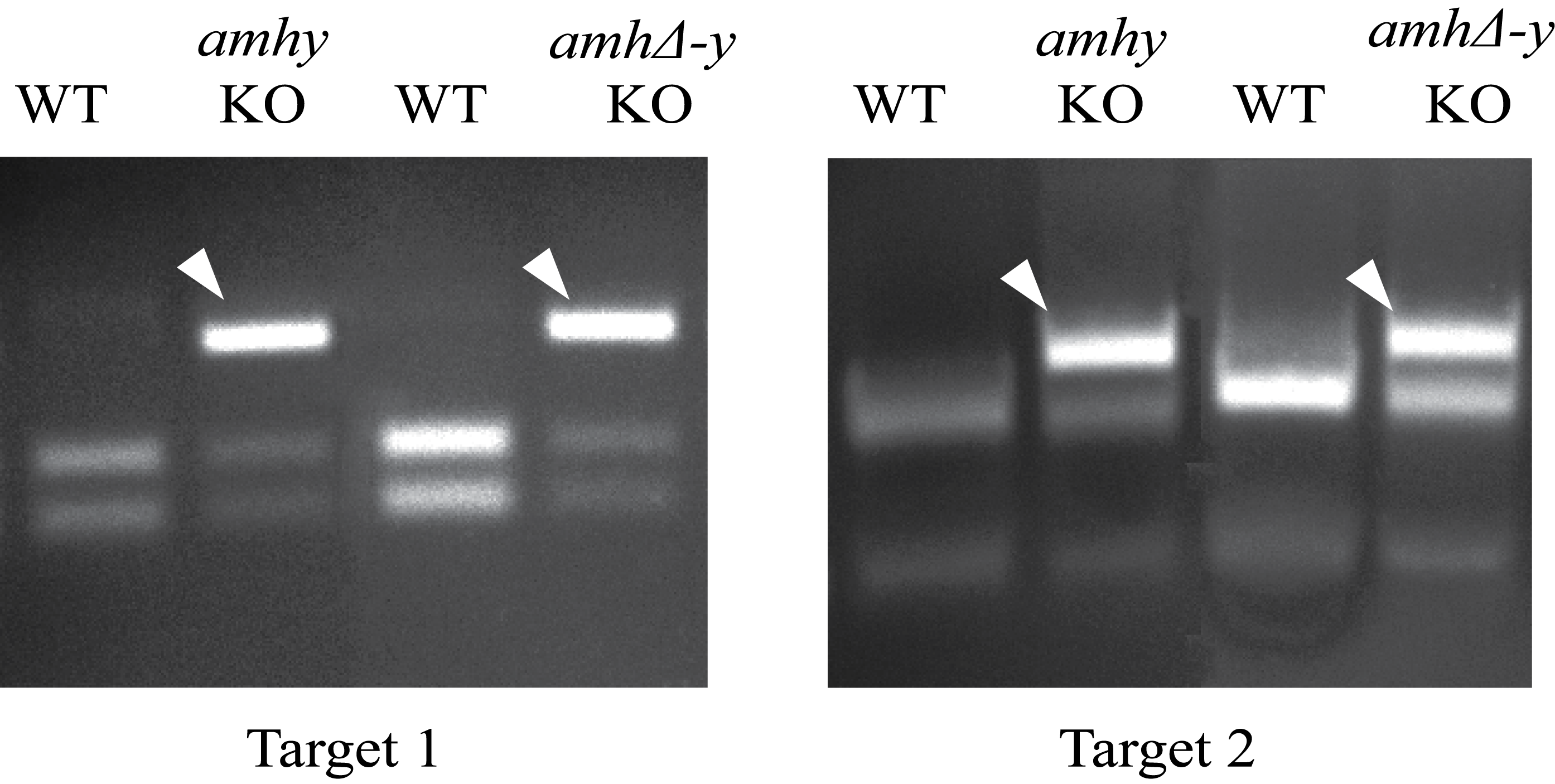

Supplement: S9 Fig — Two pairs of primers were designed to amplified amhy and amhΔ-y on the Y chromosome specifically in the genome of F0 knockout XY fish. The genomic DNA fragments spanning the target for each fish were amplified from pooled genomic DNA from up to about 20 embryos. Restriction enzyme digestion with BstN I and BsrB I showed that the CRISPR system disrupt both genes on the Y chromosome equally. (TIF) [file pgen.1005678.s009.tif]

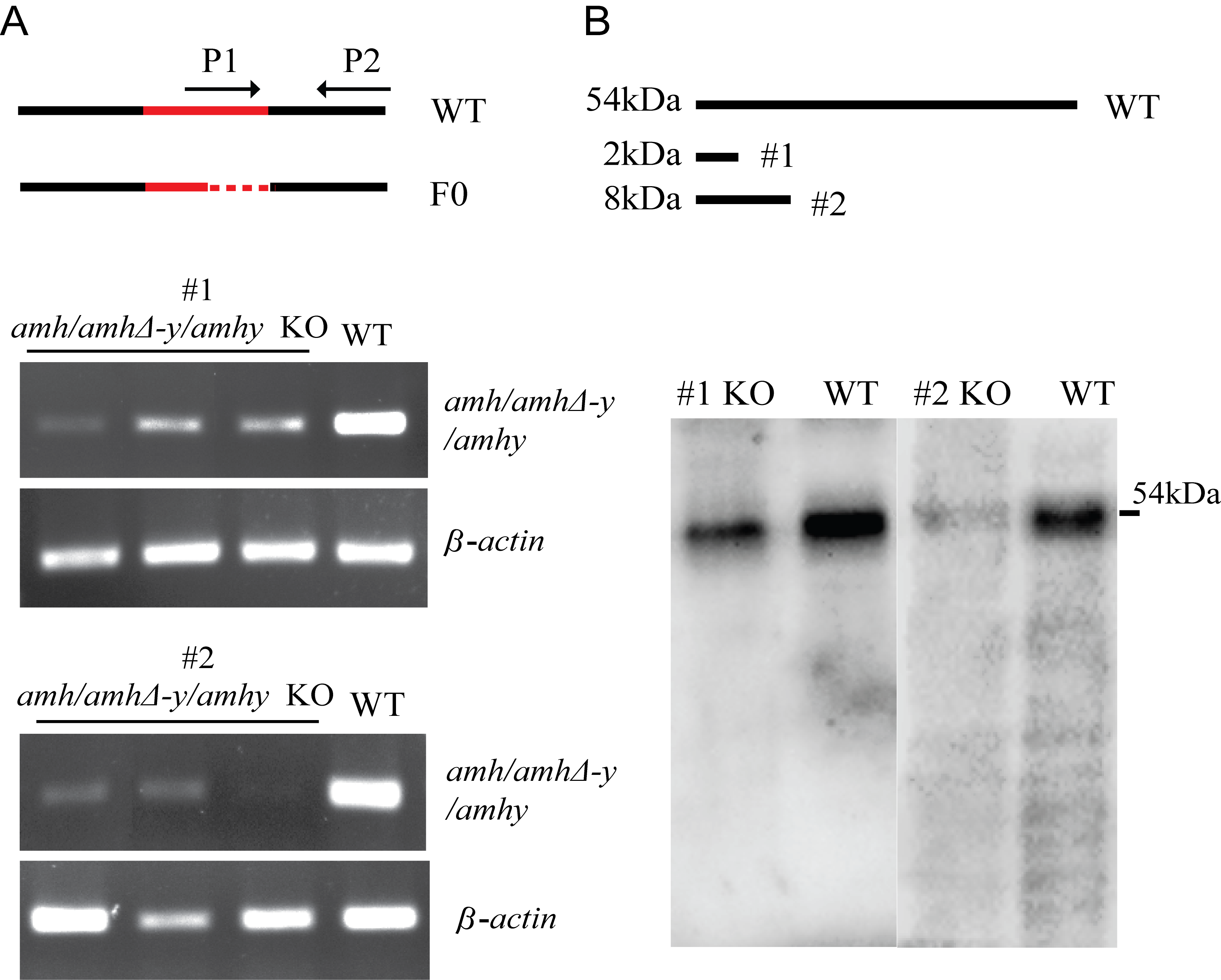

Supplement: S10 Fig — Schematic drawing of primer pairs used for detecting mutagenesis in cDNA by RT-PCR. A forward primer was designed in the target sequence. RT-PCR analysis using cDNA from 90 dah mutated gonads as template demonstrated that indels from gRNA/Cas9 resulted in less amplification of the expected amh/amhΔ-y/amhy mRNA fragments and gave weak bands in agarose gel, compared with the control (A). β-actin was used an internal control. Consistently, Western blot analysis showed that no AmhΔ-y and decreased Amh/Amhy protein level was detected in the gonads of F0 knockout fish by Western blot (B). (TIF) [file pgen.1005678.s010.tif]

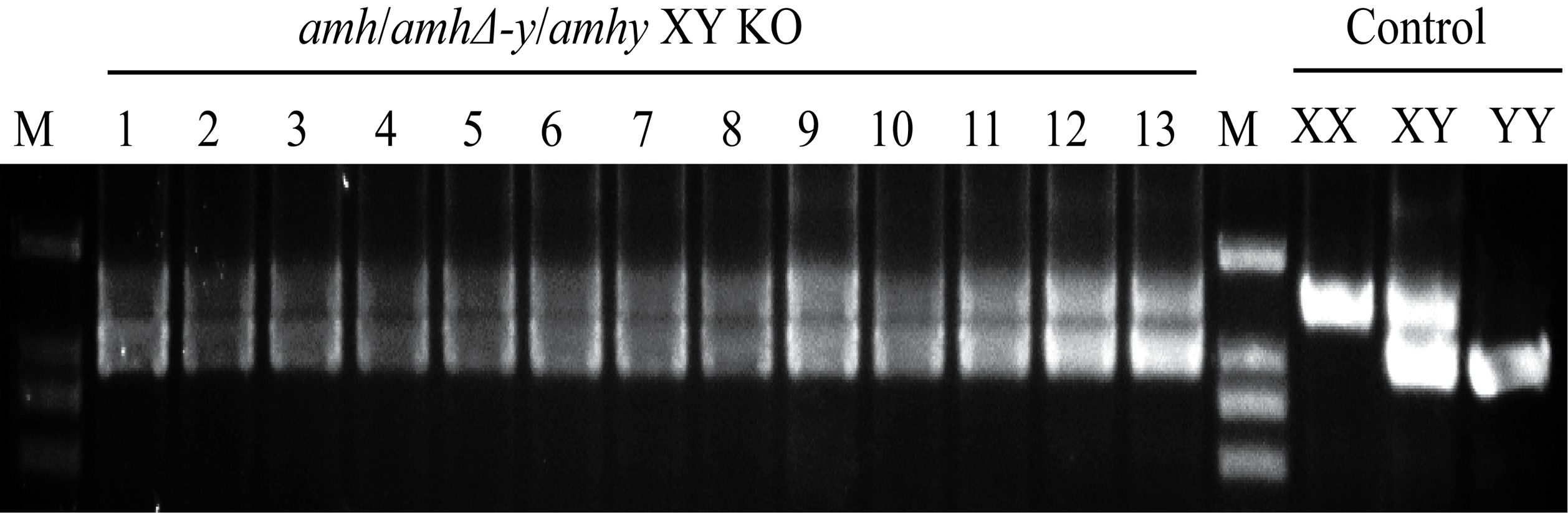

Supplement: S11 Fig — 1–13, sex-reversed XY fish with amh/amhΔ-y/amhy knockout. M, DNA molecular standard. (TIF) [file pgen.1005678.s011.tif]

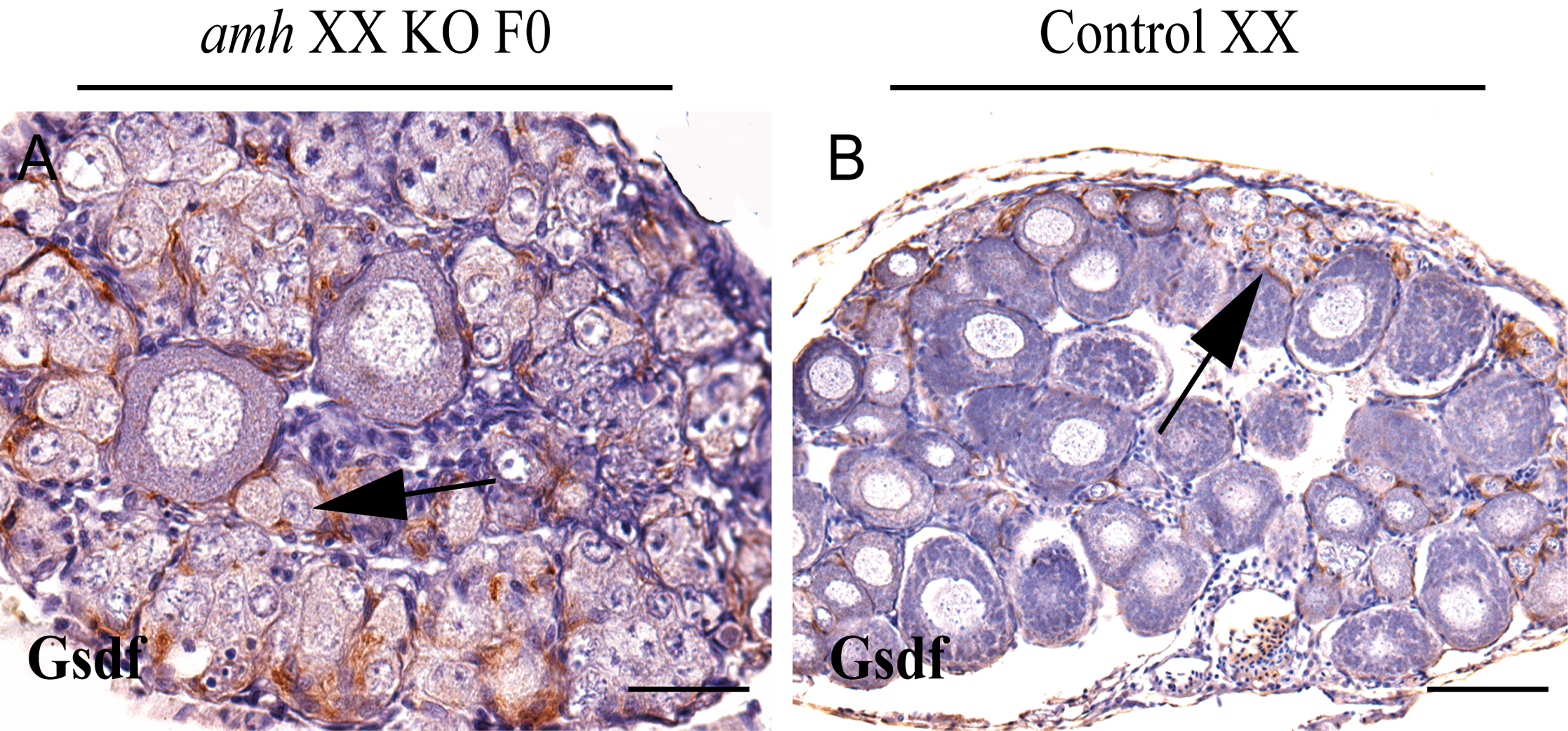

Supplement: S12 Fig — A number of oogonia and few oocytes were observed in F0 Amh knockout XX gonads at 90 dah (A), while all stages of oogenic cells were observed in the control ovary (B). Scale bars, 10μm (A); 50μm (B). (TIF) [file pgen.1005678.s012.tif]

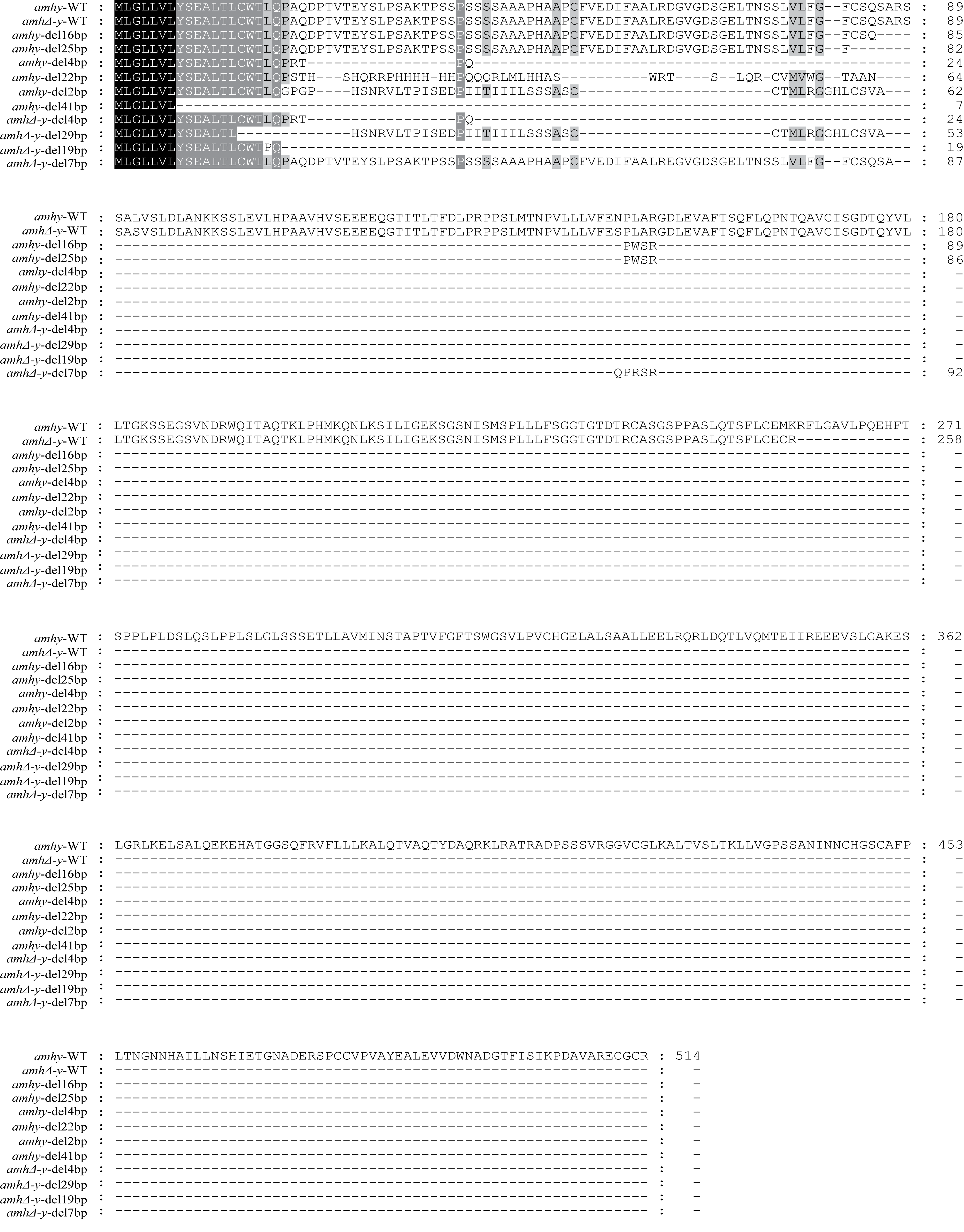

Supplement: S13 Fig — A frame shift occurs in the target 1 and 2 before the TGF-β domain to cause premature terminations. (TIF) [file pgen.1005678.s013.tif]

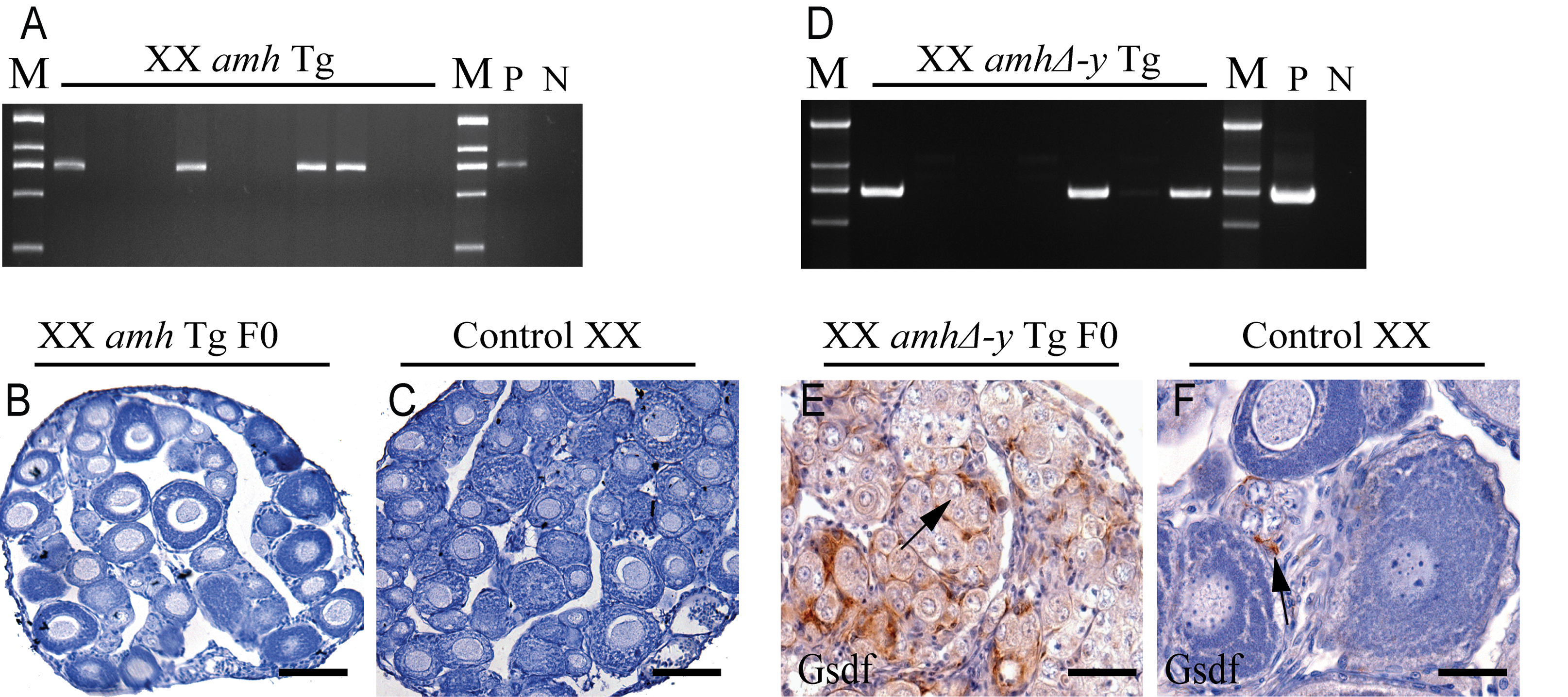

Supplement: S14 Fig — A, D, Screening of transgenic fish by RT-PCR using the GFP specific primers. P, positive control using the plasmid containing the GFP; N, Negative control using water. B, E, Overexpression of Amh or AmhΔ-y in XX fish resulted in no sex reversal, compared with the control XX fish (C, F). (TIF) [file pgen.1005678.s014.tif]

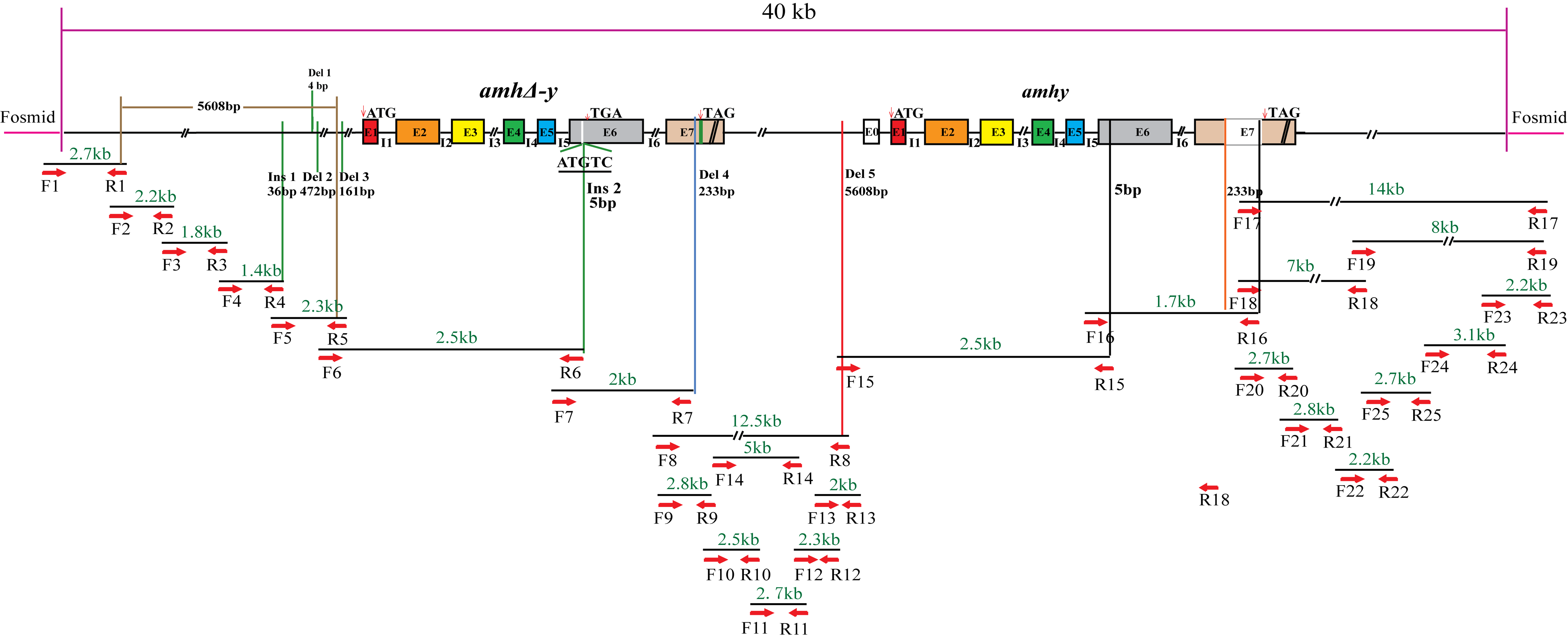

Supplement: S15 Fig — Twenty five pairs of gene specific primers were designed in the differential regions of amhy and amhΔ-y to amplify fragments with overlapping ends from the Y156 fosmid. (TIF) [file pgen.1005678.s015.tif]
