# Supplementary material for: A Tandem Duplicate of Anti-Müllerian Hormone with a Missense SNP on the Y Chromosome Is Essential for Male Sex Determination in Nile Tilapia, Oreochromis niloticus
Source: PLoS Genet. 2015 Nov 20;11(11):e1005678. doi: 10.1371/journal.pgen.1005678 (PMC4654491; doi:10.1371/journal.pgen.1005678)
Supplement: S1 Table — (DOC) [file pgen.1005678.s016.doc]

**Supplemental Table 1**

***Test of sex specific primers from the amhΔ-y region in genotyping progeny from XX (♀) × XY (♂) crosses in Nile tilapia***.

| Cross types | No. of  Cross | No. of  progeny | Sex of  phenotype | Sites of primers | | Correspondence rate (%) |
| --- | --- | --- | --- | --- | --- | --- |
|  |  |  |  | 5 bp insertion | 233 bp deletion |  |
| 68969 (♀) × 65891 (♂) | 1 | 80 | 38♀:42♂ | 38XX:42XY | 38XX:42XY | 100 |
| 67474 (♀) × 66093(♂) | 3 | 70 | 33♀:37♂ | 33XX:37XY | 33XX:37XY | 100 |
| 68349 (♀) × 66674 (♂) | 3 | 80 | 41♀:39♂ | 41XX:39XY | 41XX:39XY | 100 |
| 67404(♀) × 70278 (♂) | 4 | 70 | 34♀:36♂ | 34XX:36XY | 34XX:36XY | 100 |
