# Supplementary material for: A Tandem Duplicate of Anti-Müllerian Hormone with a Missense SNP on the Y Chromosome Is Essential for Male Sex Determination in Nile Tilapia, Oreochromis niloticus
Source: PLoS Genet. 2015 Nov 20;11(11):e1005678. doi: 10.1371/journal.pgen.1005678 (PMC4654491; doi:10.1371/journal.pgen.1005678)
Supplement: S2 Table — (DOC) [file pgen.1005678.s017.doc]

**Supplemental Table 2**

***amhΔ-y is close to neutral evolution in comparison to*** amhy.

| **Method** | **Ka** | **Ks** | **Ka/Ks** | **P-Value (Fisher)** |
| --- | --- | --- | --- | --- |
| NG | 0.00349991 | 0.0204557 | 0.171097 | 0.040667 |
| LWL | 0.00346404 | 0.0213615 | 0.162163 | 0.00473977 |
| MLWL | 0.00336242 | 0.0235227 | 0.142944 | 0.00298741 |
| LPB | 0.00364947 | 0.0244756 | 0.149106 | 0.00348304 |
| MLPB | 0.00364947 | 0.0244756 | 0.149106 | 0.00348304 |
| GY-HKY | 0.00360454 | 0.0197515 | 0.182495 | 0.00739593 |
| YN | 0.00371868 | 0.017461 | 0.212971 | 0.112443 |
| MYN | 0.00372266 | 0.0174913 | 0.212829 | 0.112443 |
| MS | 0.00364641 | 0.0191518 | 0.190395 | 0.00820685 |
| MA | 0.00364911 | 0.0195182 | 0.18696 | 0.00746972 |

Ka and Ks were calculated using the KaKs calculator program through model selection and model averaging.
