# Supplementary material for: A Tandem Duplicate of Anti-Müllerian Hormone with a Missense SNP on the Y Chromosome Is Essential for Male Sex Determination in Nile Tilapia, Oreochromis niloticus
Source: PLoS Genet. 2015 Nov 20;11(11):e1005678. doi: 10.1371/journal.pgen.1005678 (PMC4654491; doi:10.1371/journal.pgen.1005678)
Supplement: S3 Table — (DOC) [file pgen.1005678.s018.doc]

**Supplemental Table 3**

**Mutation rates of *amhy*/*amhΔ-y/amh* and *amhrII* genes induced by CRISPR/Cas9.**

| Gene | No. of injected | No. of Mutants | PR | Indel mutation frequency | | | | | | | |
| --- | --- | --- | --- | --- | --- | --- | --- | --- | --- | --- | --- |
|  |  |  |  | #1 | #2 | #3 | #4 | #5 | #6 | #7 | #8 |
| *amh/amhΔ-y/amhy-*XY*-#*1 | 45 | 8 | 18% | 34% | 57% | 41% | 56% | 32% | 77% | 91% | 83% |
| *amh/amhΔ-y/amhy-*XY*-#2* | 45 | 28 | 62% | 42% | 72% | 56% | 62% | 96% | 91% | 82% | 87% |
| *amhrII-*XY*-#*1 | 30 | 22 | 73% | 26% | 33% | 58% | 62% | 44% | 68% | 66% | 56% |
| *amhrII-*XY*-#*2 | 30 | 15 | 50% | 22% | 27% | 62% | 55% | 48% | 57% | 65% | 63% |

The indel mutation frequency within each individual was estimated by dividing the uncleaved band intensity to the total band intensity of the restriction enzyme digestion. Mutation rates of eight fish randomly selected were listed. PR, positive rate.
