# Supplementary material for: A Tandem Duplicate of Anti-Müllerian Hormone with a Missense SNP on the Y Chromosome Is Essential for Male Sex Determination in Nile Tilapia, Oreochromis niloticus
Source: PLoS Genet. 2015 Nov 20;11(11):e1005678. doi: 10.1371/journal.pgen.1005678 (PMC4654491; doi:10.1371/journal.pgen.1005678)
Supplement: S4 Table — (DOC) [file pgen.1005678.s019.doc]

**Supplementary Table 4**

**Primers used in this study.**

| **Primer** | **Sequence（5'-3')** | **Purpose** |
| --- | --- | --- |
| *amh/amhΔ-y/amhy*-#1-F | GTGAGAAGGGGAGGTCTGAGGATG | Screening of knockout fish |
| *amh/amhΔ-y/amhy*-#1-R | GCTGCTGAGGATGATGATGGTG |
| *amh/amhΔ-y/amhy*-#2-F | CACCATGCTTCGTGGAGGACATC |
| *amh/amhΔ-y/amhy*-#2-R | CAGGGTTTGCACCTGAGCTCTC |
| *amhrII*-#1-F | CAGCAAGCCCTTACAGCAGA |
| *amhrII*-#1-R | TGATGCGTGGTTGTGCCTTG |
| *amhrII*-#2-F | CTTGTGGTGTAAAAGGACTG |
| *amhrII*-#2-R | ATTTCAGCTGTTAGTGAGTGG |
| SPF | ATGGCTCCGAGACCTTGACTG | Genotyping |
| SPR | CAGAAATGTAGACGCCCAGGTAT |
| *amhΔ-y*-F1 | AAACCTCCTTCCTTTGTGAATGTC | Transgenic cDNA and gDNA validation |
| *amhΔ-y*-R1 | CAGCAGAAGAAACGCACGGAACT |
| *amhΔ-y*-F2 | AAACCTCCTTCCTTTGTGAATGTC | Y-specific fragment amplification |
| *amhΔ-y*-R2 | CGTGGCCACTCCCTCCACCC |
| *amhrII-*TD-F | AACCACGCATCAACAACCAA | Tissue distribution and ontogenesis |
| *amhrII*-TD-R | GTTTCCTCCCAGCCCCGA |
| *amhΔ-y*-TD&OT-F | CTCGCAGTCTGCCCGCTCATCAGC |
| *amhΔ-y*-TD&OT-R | CCAGGAAGCGTTTCATCGACAT |
| *amh/amhy*-TD-F | AAACCTCCTTCCTTTGTGAGATG |
| *amh/amhy*-TD-R | GTTGCCGTTGGTCAGAGGGAACG |
| *amh*-ORF-F | ATGTTGGGTCTGCTCGTTC | Antibody preparation |
| *amh*-ORF-R | TCATCGACATTCACAAAGGAAGG |
| GFP-F | ATGGTGAGCAAGCAGATCCTG | Screening of transgenic fish |
| GFP-R | TTACACCCACTCGTGCAGGCTGC |
| *amh/amhΔ-y/amhy*-gRNA1-F1 | TAATACGACTCACTATAGCTGGACCCTGCAACCGGCCCGTTTTAGAGCTAGAAATAGC | gRNA synthesis |
| *amh/amhΔ-y/amhy*-gRNA2-F2 | TAATACGACTCACTATAGAGACCGAGGCTGATGAGCGTTTTAGAGCTAGAAATAGC |
| *amhrII*-gRNA-F1 | TAATACGACTCACTATAGAGACCACCGACTGTTGCGTGTTTTAGAGCTAGAAATAGC |
| *amhrII*-gRNA-F2 | TAATACGACTCACTATAGGCTACTACTGGATCATTAAGTTTTAGAGCTAGAAATAGC |
| gRNA-R | AGCACCGACTCGGTGCCAC |
| *amh*/*amhΔ-y*/*amhy*-ko1-mRNA-F | GCTGGACCCTGCAACCGGCCCAGG | Detection of *amh*/*amhΔ-y*/*amhy* mRNA in knockout fish |
| *amh*/*amhΔ-y*/*amhy*-ko1-mRNA-R | GACCAAGAGCAGCACAGGGTTTG |
| *amh*/*amhΔ-y*/*amhy*-ko2-mRNA-F | CGCAGTCTGCCCGCTCATCAGCC |
| *amh*/*amhΔ-y*/*amhy*-ko2-mRNA-R | GTCAGCAGTACGTACTGTGTG |
| *amhΔ-y*-#1-F | GTGAGAAGGGGAGGTCTGAGGATG | Screening of F1 XY  fish |
| *amhΔ-y*-#1-R | CCAGGAAGCGTTTCATCGACAT |
| *amhy*-#1-F | GAAAGGGGTGTTTTGGTGCTGGC |
| *amhy*-#1-R | GCTGCTGAGGATGATGATGGTG |
| *amhΔ-y/amhy*-#1-nest-F | GTGAGAAGGGGAGGTCTGAGGATG |
| *amhΔ-y/amhy*-#1-nest-R | GCTGCTGAGGATGATGATGGTG |
| *amhΔ-y*-#2-F | CACCATGCTTCGTGGAGGACATC |
| *amhΔ-y*-#2-R | CCAGGAAGCGTTTCATCGACAT |
| *amhy*-#2-F | GAAAGGGGTGTTTTGGTGCTGGC |
| *amhy*-#2-R | CAGGGTTTGCACCTGAGCTCTC |
| *amhΔ-y/amhy*-#2-nest-F | CACCATGCTTCGTGGAGGACATC |
| *amhΔ-y/amhy*-#2-nest-R | CAGGGTTTGCACCTGAGCTCTC |
| Ins1-X-F | GTTTGCAATAGTTAGGGTGCTGCTG | Verification of six differences exists in the gene structure of *amh* and *amhy* |
| Ins1-X-R | GGAAATGCAGCCATTCCTGAG |
| Ins1-Y-F | ATGGCTCCGAGACCTTGACTG |
| Ins1-Y-R | TTACAGCAGCACCCAGAGTCAT |
| Del1-X-F | CAAAATTGAGTCAGGAGGGAG |
| Del1-X-R | CCTGAGCCTTCTTGACCAGC |
| Del1-Y-F | GACAACATGTTTCCTGCTCG |
| Del1-Y-R | AGGACAGATCCCTGAGGAACTCC |
| Del2-X-F | GGATCGTGGGCCACATTCAGC |
| Del2-X-R | CAGAAGACGACTTTGGACACAC |
| Del2-Y-F | ATGGCTCCGAGACCTTGACTG |
| Del2-Y-R | TCTTCGTGTCCACTATCATCTCTTT |
| Del3-Y-F | CATTGCTGAAATGCAAAGTGTTTC |
| Del3-Y-R | CACGCAACGCTGCAAAGATGTC |
| Ins2-Y-F | AAACCTCCTTCCTTTGTGAATGTC |
| Ins2-Y-R | CTAGCGGCATCCACACTCCCTCAC |
| Del4-Y-F | GCTGTGTGCATTTCAGGAGACAC |
| Del4-Y-R | CCACACTCCCTCACAACCGGT |
| Del5-F | GAAAGGGGTGTTTTGGTGCTGGC |
| Del5-R | ACCCAGGAAGCGTTTCATCTCA |
| Fosmid-F1 | GTACAACGACACCTAGAC | Confirmation of the assembled Y156 fosmid sequence |
| R1 | CTGATGTGCAGCCATGTTAAGGTCC |
| F2 | GTTGACATTGTAGAATGCATTAGTG |
| R2 | CAAGACACCGAACCTCCTCC |
| F3 | GGTACATTATTCGTAGATAATCTTTC |
| R3 | CAGCTCTCTTATGCAGGACTAC |
| F4 | GAAAGGGAAGCTTCTCTGGTCCGG |
| R4 | CTATTGCAAACCAGGAAATAATC |
| F5 | GTAGAGAGGTGAATACATGATGGG |
| R5 | CTAACAACCTGATTACCCTACATAC |
| F6 | GGACACATGACAATAAACACTTTG |
| R6 | CACCCAGGAAGCGTTTCATCGACAT |
| F7 | GTCTCAGTGATGAAATGTGCAG |
| R7 | CACTCCCTCACAACCGGTGAGAGC |
| F8 | GCTCTCACCGGTTGTGAGGGAGTG |
| R8 | CTTGAAGGATGGCATAAATTTGCCAGC |
| F9 | GCTCTCACCGGTTGTGAGGGAGTG |
| R9 | GCTTCTGTTTAACACGCTCTCAG |
| F10 | CCCATTATTCTCCATCTCACAGTG |
| R10 | CGCAGTTACTCAGCACCGTCACTTCAC |
| F11 | CGGCTGCTCCCTTCAGGGGTCAC |
| R11 | CATGTTCTAGCCTGCACTGTTAGAC |
| F12 | GCTGACACATAGCTATCATGTGTG |
| R12 | CGTGGGAGGAGAGGGGCTATGCAGGACC |
| F13 | GGGCAACGCTCCCCACCTCAGGAG |
| R13 | CTTGAAGGATGGCATAAATTTGCCAGC |
| F14 | GACATAAACCACTTCTCCTTTCCG |
| R14 | CGGTTGCAGGGTCCAGCAGAGTGTC |
| F15 | AAAGGGGTGTTTTGGTGCTGGC |
| R15 | CACCCAGGAAGCGTTTCATCTC |
| F16 | GCATCTTGATTGGTGAAAAATCAG |
| R16 | GGCGTGGTTGTTGCCGTTGGTCAG |
| F17 | CCCTGACAAAGCTTCTTGTCGGCCC |
| Fosmid-R17 | CAGGAAACAGCCTAGGAA |
| F18 | GAGACCGGCAACGCGGATGAGCG |
| R18 | CGCAATGACGCCCCGACGGTCATC |
| F19 | CATGCATTAAAATAACATCATC |
| Fosmid-R19 | CAGGAAACAGCCTAGGAA |
| F20 | GAGCGTTCGCCCTGCTGTGTGCCCG |
| R20 | GCGCAACCCTTCAGTTCATCCGCTG |
| F21 | GCCATCCAGTGGTTCATCAGCCG |
| R21 | CTGTCACTCTGACGTTTACCGTCG |
| F22 | CCCAATGTATTACTACTTAGATAC |
| R22 | CGCAATGACGCCCCGACGGTCATC |
| F23 | CAAGTTGTGTTCTGCCTTGTCCCTC |
| Fosmid-R23 | CAGGAAACAGCCTAGGAA |
| F24 | CCTCATTACCTTTTCACACTAGTTTC |
| R24 | CTCTGAACTATAGTATACCCACGGC |
| F25 | GTGCAGGCTAGAACATGGGATTAAAG |
| R25 | CAACACACCCGTGACATTTCATAATC |

F, forward; R, reverse. SPF, sex specific forward primer; SPR, sex specific reverse primer;

TD, tissue distribution; OT, ontogenesis; ko, knockout; ORF, open reading frame; GFP, green fluorescent protein.
