# Supplementary material for: A Tandem Duplicate of Anti-Müllerian Hormone with a Missense SNP on the Y Chromosome Is Essential for Male Sex Determination in Nile Tilapia, Oreochromis niloticus
Source: PLoS Genet. 2015 Nov 20;11(11):e1005678. doi: 10.1371/journal.pgen.1005678 (PMC4654491; doi:10.1371/journal.pgen.1005678)
Supplement: S1 Sequence — (DOC) [file pgen.1005678.s020.doc]

**Supplementary sequence**

***amh*:**

GGAGTAGTGTTTTACAGTCTGGCTGTTGTCCAGCTCTGAGTGAACTTAAATATCGTGTTTGAATTTAAGGTGCTATCTAATCTGTTCATATGTAGTTTCTTTGTTCTGGTGCCAATTATAGCAACTGGGATTCAGCAAAATCAGGAAAAAAAAAAAAAAAGAATTATCATTTAACGACAGGATAAACCAGGGTCTACTTCCCCCCCAAAGTTTCACTTCATGAGTCACGCACACACACACTCAGACACACACACACAGCCAGTTCATTGAACTCTGCTCAGACGTCAAGCACCTTTGCTTTTGCGTGCATGCGTTATTTCCTCCCTCGGTGAAATTCTGTATTTGTTGCAATATATTTGGTTGCGTAACAAAATGAGGAACTTTTTTGGTCTAACTTCGCACTGGATATTAAGCTCACGTAGCAGAAGATGTACAAGCCCTTGCTTGTGTTAGTTGGCACCCCTGCCATGTTCGTGATTTACTGTATTTACTGTGTTTTCATATTTAAATTTGTGACAAACTGCAATGAGTTGTTGGTGCTTAATTCAAAGCCAAATAATAAATTAGATTGTGTCTGTTTGGCTTGCTCTGACTCCTGTCTCAAAAAATATTAAATATTTCCTTAAAGTTAAGTTGTTTCTGTTTTTCTGCTAATGTCAGATATTTAATGTTAGCGATAATCAGACAAATCTCTCAAAGGATGAGAGGAATGAGGAAAACATCTGCAGATGTTGATTTTGGCTTTATTTTGATGATAAGTTAAATTAATAACATGCCTGTGGGTGCCAATAATGCAATGCAGCGTCTTGTACATTAACATCTGTTTTAAAAAAAATGAAATGGTGCTCTTCAGCCACCCGTCACTGTGGCTGCTCTGTTAAGGTTCAGCGAACAGCTGAATCCACATGTCCAAGAACAACAGAGTTCAGTGAGTTTGCGTGTATTTATTAACCGTTTCTTTCCCGTCGGTTTGTGAAGTCACACAAACGGCAGCAGAAATATTCCTCTTTGTAAGCTAAGCATAACAGCAGAGATCCATGCATTCATATTTATCCGTGCTGTTAGGAAATGCAGGTTTAAATGAAAGTGGACTGATATGTGAAAATTTACTGCGTTTAGTTTAAACACACATACACACACACAGTCATGCATGCAAATCCTCACACACTGGGAAAACATAGTTGACCATAACCACAACAAACTACTGGACTTGTTTCACACCTCTTTGTTTGCTTTTTAAAAAACGGTGTAATTTGTTTTTGTAAACAATGTTTGTACTGTGAATGTGATTCATTCTCTGACTGTATAGTTGTGGATAATTTAGATGATTATATTTATGCTGTCGGTCTTTCCATTTGCCTTTTTTTTTTTTTTTATAATTtAGATGATTATATTTATGCTGTCGGTCTTTCCATTtGCCTTTTTTtgTTTtAAaTAAATCCTGAATTGTGTTGACATTGTAGAATGCATTAGTGTGTGTGTACTTGACTCACAGTCTGAAAACAATGGTGCTAAGTTTGGACTATGCCTGATCTTATTTATTCTGTAATGAAAACATCAAAAACAAGTCAAGTACTTGGTGCTGTACACAGGGACATTTGTAAAGCCGTTTTTTGGCATTTCCTTTCTCATATTTAATATGCTATTGTCTCGTTCTTTGAATGCATCGTGTACAACTGTAGACGCCACCTTTGGATGAAAGTGCTCTCGTGCAGCCGGCTCGGGGCCCGAGCGACGGCCACGGGGGCCCCTGTTAATGTTTTAAACATACATCGGCCCGCTGTTGGTGAACAAGACACACTGTTTTTGTACATAAGTTTTCTTTAATTGTACATCTCCATCATAAACAGAAAGGTATATAAAATAAGTAACTATATACAGTCTATATAAATATATATAAGTATATAAATATATATACATATATATGTGAAAGGGGTGTTTTGGTGCTGGCATTGTGACCCTTAAATTCACCCTGTAACAGGAGGCTTGAAGCTGAGCAGAGCAAAGCATTGCCACCAGAGGACCTTAACATGGCTGCACATCAGCACCTGAAAACACACACACACACACACACACACATCCCCCGCAGCCACAAACTCAGAGGCTGTGTGTTTATTACACAAAGGTGCGGCGGCTCAAACGCCACCAGATTCCAGTACTAGAACTGTTATCACTTGTTTCAGGTATTTTCACTTCTTTGATTTATTGTTGTTTTTTTTGTTTGTTTTTGGTGTTTAATTTTTTTACCAACCCAGATTCTGCAGGAAAAATGGTGCTTCTGTTTCCAAGCGAGGGCAGTTCAGACCTTACAATCAAAATCTTCACCGGTGCATACAGAACTGCGCGTCAGCCTCTTTACACACACCGCCTGTAGACGTCTTACTGACGAGGCCGTACCTCGCCGCGTGAGCTGATGTGAAACACTTGTCTTAGTGTGTGTGTGTGTGAGTGAGATCAGACAGGCTTCCTTTTTTCCTCTGATAGTGGGATTACACTAAAGCTCAGTTTGTACGTGTGTGTTTTTGTTTGGTTTTTTTTTAATTTTTCACTTGCCAGCGCATATCTAAGGGAATATTAGTGTAATAATCAAAGCTTTATAGAAAAATAAAACTCCTGTCCCGTGCACTCGTGTGCAAGTGAAGAGAAAAAGTCATTTTAGAATTAGACTAGTCCATTTGTTACCAGCACTGAGAGGTATTTTGTAATTATTGTCGATGGCGTTTTGATTTTCTTATTCTTGGTGTTTGATACGATTGCAGTTACATGTTATGCTTTTGATTGTGTCTTCAGATTTCTGTCAGTTTTTAATACTACTAGTCAGACAGCACGGTGCTCTCCTTCACTTAAAACAAGGAAATCGCAGCTATTGATGAATACTACTCTCCTCCTGTTGTGCTGCAGGGTTTTATGGTTTAAGAATCTTACACACAAGCTCACCTGGTGGCTTTTCTCACAGCCCAGTCTAACCTTTGAATCTTTAATCTGGAGGGACACCGGCGGGCGGAGGCGGGGGTGTACGTGCCTGTCAGAGCACTTTGAGGAAGGTGGGAGGTATTCCATTCATTACATACAGATTTTAGGCAGTTTTATAGTTTTTAAAAGTATTTCTGGGGTGTGATTTGGCTTTAAAGCTGTGCAGTTCTGCATCATACTGCATCCTGGTACATTATTCGTAGATAATCTTTCAGAGCTTTGCAGTCAAGGCTGTTTACTCGAAATCCTGCCAAAGGTCTTAACTCAGGAACTCCCCACACTGCTTGAAAGAACAAGTTTCCTAATCTGCTGCATGTTACGTTAATTTACCTTCAGGTGGCATGACAAAGTCCCTCTACGACCCAACTCAGGACCAGGGCCACCCTTCAGTCAGTGTATTTCAAGGTAAAATGTATCAGCTGCCCTCATCAGATCTCCTCAGACACAGTCCTGCCGGCACGTACACCCCCACCCCCGACAATCAGTCCGAGCTTTATGAGCGATCTGATCTCGAGAAGCTGTGAAATGGCAACGACTTTCAGCATCTTCCTCATCTCACAAATGCCTCTGTCTAGATTTGCGTCTCTAAGGAGGAGGTTCGGTGTCTTGTTTGAATCCCTCTTTGAATTGGGGGTAACATTCAAGGACACGCGTGACCTACAGGATGTGTATAGTGCTCTCTGATTGGTGAAGCCAGGGCAGGTTTGAGGTGCAATAATGGCAAACCTACTTTCCAAAACGCCCTCGGTTTTTCATGGAAACATTGTTACTGTTTGATGTTGGTGCTTTTATCCTAAGATGTTATAATGAAAAAATGGAATTAATGTTTTTTTTTCTCCCCTGCGATTTATGATAATTAATAAAATGTTTCTTATAATATTGAGTTTACGTTTGGAGTGAACTTTGTCTGAGTCCATTTGGTTTGCCGTGACGGAAGAGGGTGTCATGCTGATATCTTCATACCTGAACCATGTTTGGAGCCCCGCTACCTAAATCCTCAGTTAGTAGACTTCACAGGTAGGCGGCATTTACCTTGTAATGCCAAGTCGGCCCGGCAAAGACTCCAATCTGGCCCACTCGACGGCAAAATGTGAACTTCCACAAGTTTGATAGTTTTTCCTAATGAAAAGACAAAGTAACCAACAAATATATAAACCCTTAAAGAAATTTTTTTTTAATCTACTATAGCAGTTTGTTTTTTACAGCTGGTCCACAGTATCTGTTGTGCTGACAGATTTCTTCAGTTCATTACAGCAGCACTTCTTAATTGAGTAGAAAAACAGATATGTGGTTGAAACTGCACTTCCTGTTTCCACACGTCTCTGAGGTCAAATGTGTAGGTAAACCTCTTCACAGAGACAGAAAGGGAAGCTTCACTGGTCCGGTTCACAAAAGTTCAAAGTGGGCTGCATGTGGCCTGCGATGTAAAATAAATTGATTAAAAAATGCTTCTCTGTATAAGATACTCGAGTTTTTGAGAAGTCAGCATCATTAGTGATGGGTGTGTGTGTGTGTGTGTGTGTGTGTGTGTCAGCCCTGAGAAAGACTAACAGCCTGTTCACTGTGGTCCTGCCCTCCTGCAAGCCTGAATCAGAGAGATGGATATGAAATTGATGGATGATTAAAAACCACAATTAATGACATTGTTTTAAAAAATATGTCAGCTTTAAAAAGAAATGGAGAACGTTATAATAAACATAATGTAGTCCTGCATAAGAGAGCTGCTTCAGTCACTAACTTGAGGATTAATGGTCCAGTGAGGTCTGTAAAAACAAACAGCTTCTGCTGCTTCAGTTCACAGACGTGTTACCTTATTCTGTGTCGAATGCCTGTGATGCCTCAGAGAAAGCCTCCCAAAAGGTCCGTGCACTGAAGCTTTCCACCTGAAGCCAGGCCTGTGGCGTTCCCCGGTTACCGCCTTTGGCTTCGGGGTAATGAGGATACAACCTTACAACATGGACAGATGAGATTCCCCAGAAATGTATGTCTTCTGTGTTTTTGTCCTTTTTCCAACCTGGAAAACTTAAGTGTGCTACTGTAGAGAGGTGAATACATGATGGGGAGAAACAGGAGTGTAGATGTTGATTACTTTTTCATCAAAGTGCAATTCCTGGTTCCCACAAGTGTGACTGAAAACATCCAGGCTGGTGTGCTTCAAACACCGCCTTGATACAATGAACATTTCCTGCCATTCCTCACTGATGATTTATAAGGTATGGTGAACCTTGGCTTTATCACCGGGGGAAAACGTCCACAAATGGCTCCGAGACCTTGACTGTCTGCACAACAAGCCTGCAGCTGTGAGAATGCTGAGGGGAGTAACAGCTCCTCGAAGCCACTCAGGTCTCCCTCTGACAACATGTTTCCTGCTCGTCTTCCAGCGTGAATGCTTCCAGATCTCCAGTGTGGGTTCATTAAGATAACCCATTCTCTTCCCATGACCTTTAATGGTAAGTTCACCCAAAATGCAGGCAGACCACACAGTTGATGAACAATCTGATTCATTTCTCATCTGACATAGATGAAAATAAATTAATGACATTTCAGTTATGAAACAAACTAAAGCGGATTGGAACTGTGAAATATTTTGCTCCCGAACAGTTTCTTTTCCACAAAGCTTCTCTAAAAATTAGAAAAAGATATTAACTGAATGAAAATTAAGCAAAATAAAACAAATAGCAGTATATTTTGTACTTAAAAACATTAATATAAAAAGTGTTAAAAGACTGCAGTCCTTATAAACCAGATTATTTCCTGGTTTGCAATAGTTAGGGTGCTGCTGTAATGTTTTTATGAGCTGTAATAGTTAATTTTTTCAGTTTTTTAAAATTGGACTCTGAAAATAACGATTCAAAAACTGCATTTGCAAACACTGGCTTTGAATTCATTGCACATTTTGGCTTTTTCATTCAAAGACTTCATGGTTCAGTTGGTTTAACTGGTGAAGTACTGAAGGTTTCATGTTTTAGTTGATCAAATCAAAATTGAGTCAGGAGGGAGTTCCTCAGGGATCTGTCCTTGGTCCGCTACTCTTTAATATGCTCATAATATGTCTTATAAAAACAGATCAGACTTTTATTTAGAGTAGAATAAAAGCCTTTTAACAGTGTTTAGTAGTGTCACTAGTGATCGTACATTTACACACGGAGCCGGGGGCAGGAGATGGTTAGCCTGAAGCAGGGATGTCCAACTGATTGTGGATCGTGGGCCACATTCAGCCCACTTTGGTCTTAAGTGTGCCTGACCAGTGAAACGCCCCGCTCTGTCAGAGTAAAGAAGCTTAACTACATGTGAATCTTAATATAAGAAAGTGCAACCTCAACTCTACGTCTCAGTTTTTCCATATATAATGTAATGTCAAAAGTCCTCAGTCCCATCCTCTACACCCTGTACGCTCACGACTGTGTCACCTCCAACAAAGATAACATCATTTTGAAGTTCTCAGATGGCACTGCAGTGATCCGTCGCATCACTGGAGGGGACGAAGCGGCTTACAGGAGAGAGGTGGCCGGTCTGGTGTCATGGTGTGAGGACAACAACCTCACCCTCAACACTGACAAGACAAAGGAGATGATAGTGGACACGAAGAAGAAGAGGAGGCCTCACCAGCCGCGGTTTATCCGGGGCCTTGAAGTGGAGAGGGTGAGCAGCTTTAGATACCTGGGCGTCTACATTTCTGTGGACATCACCTGGACACTGAACACCACACAGCTGGTCAAGAAGGCTCAGGAATGGCTGCATTTCCTGAGGAGGCTGAGGAAATTTGGTATGATCCTCAGCAGGTTCTACAGCTGCATTGTGGAGAGCACACTGACCAGCTGCATCACTGCATGATACGGCAGCACTACTGCCATGGACTGCAATCACCTGCAGAGAGTGATAACAACTGCAGGGAAGATAATCAGGACTCCGCTGCCCTCTCTGCACAGCATCTACCATCGCAGAGTCCAAAGGAGAGCTGCCTCCATCCTCAAAGACCCCACACATCCCCAACACGGACTGTTCACACTTCTGCCCTCGGGGCGGAGGTTTAGAAGTGTGAAATCCAGAACATCCAGACTCAACAACGCCTTCTTCCCCACTGCCATCAGACTCAAACAAGCTGACCTATTTTTGAACATTAATAATAATTTTTTGCACATCAGGTCATCCTGCATATTAAGCCAGCATATTAAGCTATAGCTCTTTTGCACACATCTATGTTTCACACTTCAATAATTTGTCTCCTTTTCTGTCTTTATTTATTTGTGCTATTTGTATTTATCTGTTCTATTTCTACTGTAAATATTAACCGGCATCTGTGGGTGGACAGCAAAGCAAAAGTTTCATTGCACAGAGAAATGCCTTTTTTCTTTGGACACATGACAATAAACACTTTGAATCTTGAAATCAAAGAAATTTTCCATTATAAATAAATAAATGTGTAATATTACAGAAAAAAACTAATTTCTGGAGCAGATGGTGAAAAAAACAGGAACTTTTTTGTCATGTAATTGTTAATCACTTTGGTGTAGCATGAACATGTGGCAGAGTTCTTTATTAGCAGGAAAAGCCATAAAATTTAAGAAAATACAGTTAAAAGTCCAAAATTTATGCCATCCTTCAAGTGAGCTGGGTTGGAGTCATTGCTGGGTGATTCTAGCCCCTGGGCCTGATGTTGGACATCCCCCGCTTAGAGAAACAGAAAGATGAAAAACAGTGGATGTGGCTGAAGTTTCCCTTCAAGGCTTTCTGCCAGCAGTCTCTCTAAGAGGAGTCATCAGTCCAAAGCAACAAGTAAGCAGATTTACCAAAATGCAAAGTGTTTCTTTAAAATCGTTTTGAGCTTTGTTGTCGACGTGATGGAAGCAAACCTCCAGTTTTACTGTCCTCTTTGTTTAACCCTCTCAGGCTCAAATTAAGTTTTAGTTACAGGAAAAATCTGATATTTGGGGAAAATTATCAAAAAAAAAAAAAGACTCATAAAATATGTATGTAGGGTAATCAGGTTGTTAGTTTTTAACTGTTGCAAATCGGCAACGCCTGCCTCGAGAGGGTTAAGTTAAGCAGCGTGCATATTAGCTCTGCAGCCACGAAACATTAAACAGCACTCTTCATCTTTCAGCCTGGTGTATTCTATGAAAGGTTCGTGAGAAACTTTCTTTCCGGGACCTGTTTGTGTCTGCTAGATAAGGATGTTCAGTGTGTCCAAAGTCGTCTTCTGTTTACCTCCGTGTCCCGTTTGCTGTCTGATTTTAGAATGTTCTCCACAAGCAGGTTAAAATACCTCAAATACTGACAATCTCAGTCATCAAGGACACACAGTGCTGCTATTTTGGCAGAAAGGGGACCCCAGGTGGTGAGAAGGGGAGGTCTGAGGATGTAACTCCCATCCTGTGAGAGATCTGACCACACAAACGCACACGCAGACACTGCAAACATGTTGGGTCTGCTCGTTCTTTACAGCGAGGCGCTGACACTCTGCTGGACCCTGCAACCGGCCCAGGACCCCACAGTAACCGGTCAGACGCCGCTCAGCTGATTAATTAATAACTCGTTAACTTTGAATGGAAAAATGCATGCATGTGGTTATTGCTTCAGACTGGTTCTTTAGTGCGTGAAGTAATTTGTCCGTTTCAGAGTACTCACTCCCATCAGCGAAGACCCCATCATCACCATCATCATCCTCAGCAGCAGCGCCTCATGCTGCACCATGCTTCGTGGAGGACATCTTTGCAGCGTTGCGTGATGGTGTGGGGGACAGCGGCGAACTGACAAACAGCAGTTTGGTTCTGTTTGGATTCTGCTCGCAGTCTGCCCGCTCATCAGCCTCGGTCTCGTTAGACCTCGCTAACAAGAAGAGCAGCTTGGAGGTTCTGCACCCAGCTGCAGGTAAAAGCTGAAACCGCATTCCTGCTATTTTTCTTTCTTCATTTGTAAATTGTTTGGTTTCGTTCTTTTAGTACACGTATCAGAGGAAGAGGAGCAAGGAACAATCACGTTGACCTTTGACCTCCCACGGCCTCCATCGCTCATGACAAACCCTGTGCTGCTCTTGGTCTTTGAAAATCCACTGGCACGAGGAGACCTGGAAGTTGCTTTCACTAGTCAGTTTCTGCAGCCTAACACGCAGGTAAAGAGAGCTCAGGTGCAAACCCTGCTTCAAGGCTGCTGTCAAACATAAGGCCCGGGAGCCAGAATGGGCCCCGCAAAGACTCCACACTAGACGGCTTTGAGCTACTGGACGTTTTTCTGTAATTTTGCACATTTATTTCCTTCATTTATTACAGAAACCTTTCTCTGTCATGCACAAAAACTGAAGCTTGGTGTACAAATTGCACTTGTTTTTTTTTTCATATTAAGATGTCTCAGTGATGAAATGTGCAGTTAAACTTGTCACACTCACCGAAAGAGGAGTTTCACTTAAGATCACAGCGAGCTGCATCAAGTGGCCCTCAATGTAAAACGAGGTTGCCATCCCTGCCTTAGGTGATAAAATGAAATGAAAGGTTTACAGATCTCTAACTCTGGGTTTTTGTGTTGCTTTGTTTTCCAGGCTGTGTGCATTTCAGGAGACACACAGTACGTACTGCTGACAGGAAAATCATCAGAGGGGAGTGTTAATGACAGGTGGCAGATTACGGCTCAGACAAAACTCCCTCATATGAGTGAGCTATCATCTTCTTCATTTTATTTCCCCATCTCTGGTTCATTGTGTACCCTACTTACATTTCCTCTCATTGTAGAGCAAAACCTAAAAAGCATCTTGATTGGTGAAAAATCAGGAAGTAACATCAGCATGAGTCCACTTCTACTTTTCTCCGGCGGAACGGGAACTGATACGAGGTCAGCCCGGCTTTCTTTCTGCTGATATTCACTGTCATCAGAGACGCGCTCAGCTTTGGTTTTTATTTTTGCTTTCCCAGATGTGCTTCAGGCTCGCCCCCGGCATCTCTGCAAACCTCCTTCCTTTGTGAGATGAAACGCTTCCTGGGTGCTGTTCTCCCTCAGGAACACTTCACGTCCCCTCCACTTCCTCTGGACTCCTTACAGTCTCTGCCTCCCCTCTCGCTTGGCTTATCCTCCAGCGAGACCCTGCTGGCAGTAATGATCAACTCCACAGCTCCCACAGTCTTTGGCTTCACGAGCTGGGGCTCCGTGTTGCCGGTGTGCCACGGAGAGCTGGCCCTGTCTGCTGCACTGTTAGAGGAGCTCAGACAGAGACTGGACCAGACTTTGGTGCAAATGACAGAAATAATCAGAGAGGAAGAGGTTTCACTGGGAGCCAAGGAGAGCCTGGGGAGGCTCAAAGAACTGAGTGCGTTACAGGAGAAAGAACATGCCACAGGTGACATGTGCAAGAACAGTTACAGTTACATAGGAAGCATGTTTTTTTTTAAATGAAACAACACCCTGCAGCACGGTCGGGTGCATGTAAGTATCCCGCTGCAGCTTTCAGCTCCCAGCTTTAGGAGATGTTGGGTGGAGGGAGTGGCCACGGATTCTCTAAACCCCATTTATAACAGGCCTTACAGTCAAAGGTTTATGGGCCGAAAACAGTAATCATGCTGCAGACTGTCTGTTCAGTTCTACAGCGGTCCCAGTGACCTATGAGTTCAGATTATTTTAAGTCTTGTGCACAAACAGATTCCCATTATTCAAATTTTAAAAGTTCTGAAGTCGGCAGATTAAAAAAATAAATAAATTAGATCTCATTCATCAAAAAACTAAAAACCTGCATGTGAAATATTTAGATAGATATCAGCAGATGTCTTTAAGAAAACTAATCATGTGAGTGTGTGTGAGCTTGGCTGACGTTCAAAAAGGCTTCGTTTCTATTATTATATCATTTGCTGTTTTGTCAGTGGTAAAATTTGGGTCTATATTACCCACTGACCTTTGACATATATAGATGAGCAACTATATTAAATTTAATTTTATTAGATTTTGTTATTTTGTCATTTTCCTCTGCTGTGTTCATCAGTTTATCAGCTCCACAGACTCTACAAACTGGGACTCATTTCCACTTTTGCTTTTTGCTTAGCGTGAATTTTGTCGTCACCACTCTAAAGGTCACGTATATCCCTTTTTAAGTGCTGCATGTGTTTTCTTTCTGCGTCCGCCAGGAGGGAGTCAGTTCCGTGTGTTTCTTCTGCTGAAGGCTCTGCAGACGGTGGCCCAAACGTACGACGCGCAAAGAAAACTGCGGGCCACCAGAGCAGACCCCAGTTCGTCAGTGAGGGGCGGCGTCTGTGGGCTGAAGGCTCTCACCGTGTCCCTGACAAAGCTTCTTGTTGGCCCAAGCAGCGCAAACATTAACAATTGCCACGGCTCCTGCGCGTTCCCTCTGACCAACGGCAACAACCACGCCATCCTGCTCAACTCCCACATCGAGACCGGCAACGCGGATGAGCGTTCGCCCTGCTGTGTGCCCGTGGCATACGAAGCCCTGGAGGTTGTGGACTGGAACGCAGATGGGACCTTCATCTCCATCAAGCCAGATGCGGTTGCGAGGGAGTGTGGATGCCGCTAGAGCTGCTCTCTTCTGCTACTTTACCCCAGCATTTATAACTTTCACACAACTGATTAGTTATGCGCATGTTTCAGTTATGAAATATCAATTACAATACACCACGTGTACTTCTAATTAAATATAACTGCTTTTATGCTTTATTAAAGATCACAAGTCTTAAGATCACAAAGTTAATGTTAATTCTATTTATGTTAAGATATTTTGATAATTAGTGATGCTAAAATGAATCAGATTTTGTCATTTTGAAAATATTTCTAATTGGAATAATGTTTCTGAGTGTTAAAGTTAAACTGTTAAAAGGATGAAATATAGCCTTCTCAAACTTGTAATAATTAAATGCAAAGTGTACTTAAAGTTCTGTATTCATAGCCATTCAGCTACATAAGCTACAGTATATGTTTTATGCTTTGATTTTGGGTTTCAGGATTGCTTTATTAGCAAGTGGCTTCTTCCTCCTTCTTATCGCTCCGACTTCTTCAGATCCCCACCACCGGGTGAAGCCGTCCCATATGGAAACCTGGAGGCAGACAATGGGACACAAGACACGTGTGATTCCATATCAACCAGCCCTAAATACTGTGGAAATCCTATAGTGTTGCATAATAAAAATTCTCATATATCATAATATTTGAGCATGTGATATTTAAAGAGATGATCCAAAGTCCAGTTTATTTTTGGTCCTCTGAGCTGTAGAGCTGTTTGTCCAGCTTGAGCAGTTTAGTGTGCATTTCCTAGACTTGGAGAAACCGGCTGTAGACATTTCTGCGCTCTTTGGAACATTTATGAAAATTATTATTGCTATATTATAAAATTATTAAGCACACATCTTGCAGTACCAAAAATACGATAATGACTCTGTTACTCTCAAAGATAGCTGTGAAAAGTTCGGCATCCATCACCCAAAGAAGTATACACTAACCTGTGATAGCCAAGGTTATTACTGTTAACTAATACTAACTTGTTACGCCGCTCTGCAGCCCCTAATCTCTTTGCAGTGGCAGTGCAAGAGTGTTCAGCTGTTGCTAATTGACCACACTTAATCAGGTGTGGATAAAGGCATACTTGAGGCAGGCTTTCATGGAGGCGCACTAGTCTTTGTCTTAGTGGTACCAGCTATTTTAGCTAACTTATTATATAATTTAAAATAAAACCTCTTCTCTGTGAAATTCTGGTGTTGGTCTCCTTAATTATGTTGCAGCTTTTGAGCTGGCTCGTAACATAACTAAAATTAAAATATAGAGGAAATATTGTGGAGTTTTAGCATCTGTTAATCTCGTTTAACCTGTCAGCTGATGAGGTTTTGATAAACGTGTTTGAGACTTTGGATCTCTACCAGACAAAAACTTATAGTTTGATTGAAATATATGCTCTGCTTTTTCTAAATCTTACCTCTGACATACAATAAAAGGTTAAAACTAACACTGCACCGAATAAAAACTAAACTAAACATTTTCGAGCAGTAAAAACAAAAAAGTCAAAATAAAATAAAAACTTATTAGAGAATACAAAAAACTATAATAACTCTGGTGACAGGATTTAATCGTGGCGCCACCCTTCAGCTGAGCTACAGCAAGCTGCAAACCTCTTTTGGTTGGTATTTGTAATTCTCTCTGTTGTGAGCATGAAACCGCTCACAACAGTCTCATTGAGGCATACATCTCCAAAAATGGGAAAATCACACCAAAATAGTCCAGATGGGTGCACAGATGATGTCATCTAGACACAGGATGCACTCTGATGTCTTTAACGCTTAAAAACATGTGGAAGAATCAGAAACGTGATCTTGATTGTACAACATAAAAATCTTGATGTATGTAAAATAAGACACACTATAAGATACACTTCTAATGTTGTTCTTTTAACACACAGAAGCTCCAAATGCTGTTCACTGCTCTAGTCCACAGGATCACCAGGCAGCTCTGCTCAGTACACCAACACCTGCACCGTACACAGTGTTTCATGCTTTATGCTTTGGGTTGAACAGCTACGGCTATTGTCAATCTTGCAAAACTTTATTTTTTCATCCATGAGAGGGTAGGTTTGCTCATTCAGTCTTTAAAAATGGGAATAAAATTGTTTAAACCAGTAAGATTACATGTAAGGAGATATGGAGCCATCCAGTGGTTCATCAGCCGTGTGTTGACCGGGTATTTGTTAATTCTCAGCCATACAAACGGACACTTTACGGTTTCCTTATTTCCAAATCTGACAAAGACTTTCTCAAAATCTCTGTGAACTTACAGCCGGCCCATTATTCTCCATCTCACAGTGTTTATCCTACTGTTGTTGCTTTATGATGCCAGGACAATATGGCCCTCTCAGACTGATAGCTTGTCACTGAGATGCTTTGAACAGCAAGAGAAGAAGATGGATTACGGCTTTAAAGGCAGAAAAGCTAAAATGGCTCATTTCAGACAGCGGATGAACTGAAGGGTTGCGCTCATTAAAGAATAATGTTTAAGATATATGATTAAAAGTCCAAGTACTAAAATACCGAGCTGGAAATGAGCACGACTGTGTATTCAGCCAGAAAACAGAGTAAAAAACTTTACTGTATCTGTAAATTACACATAAAATCACCCTCAGTCTTTAAACAAGAACAAATGAACCGAAAGTTGCAGGCTAACTGACAGAATCACAACGAGAAACCATACCTGTGTTATTGAAACACTCTGCAGACTTCCAAAGAAAGACCACACACCTGTCTCTTCAACAAAATAGTCCAGAGCATCTAGAAAACACAATTGCACAAAAGACTGAGAGCGTGTTAAACAGAAGCTGTCGGCTGTTACAAATCACAGCAATCAGTCAGAAACAAGCTCAAAACATGTGTGATGTGGGTAAAGAGACAGTGCACCGGCACTCACCGTGCACTTCGTTAACTCCTGAGCTCACCAGTAGCACAACCAGCGCCGCCACTAAACAGCGGTTCATAGTGAATCCTTTCCACGGATTTGGAACATCGCTCAGATTTTCAATGCACAGAATCCGCTCATAAGACACTGCAGACAGACACGAGTCAGACGCATTTAGTCCATGCTCAGTTTCATCAGTCTGGTGGCTGTTTAGTGAAACGTGTGTCTTACTCGTGAAGCCTTCAGACAGGCCTGCTCCAGCTTCTTAAGGCTGAAAAATAACACTGCAAGATGAGGAAAGATTGGGTAGGACTGTGTGGTTGAGATTTAGTAAAGTAGCAGTACACAGAATACACAGAATGATCACACGTGGAGTTTGTTTAATCCCCGATATCTTATATCTGTGATCATATCCCACTTTCCCCTGTACTGCCTTTTGCACCCTCTTCCAAGCCATACACTAACATGTGACACTAACACAGCTTAAAATAGTCACATGCAGCTCTTTAGAAAGACAAATATGGAGCTGAGCGTTTAGTTTGATACAAACCTCTGGACAGAAAAATACGCTTACCTGATTTTCAGTTGTGCTTAACCACAGAGGTGAGGGATAGTAGAGCTGTGCTGTTGCGGTTGTGTACACGCTCTGCACCTCGCTTCTTTGCTTGATTGACAGGCACAGGTGTGAAAACAGGGAAAGGGACATTCAAACTTAATAGCAGTAAGCCAGTAGGTGAACGAGGATCAGCAACACCCGCCTTCCAGTGCCATGAACACTCCTACTCCTCCACAGAGGCAGATAAGCCAGCGTTACTAGGGTGGTTTAACAGTTTACTTAATTATGAGGAGGTCCTAAAACACAATACATTGTTAAAAGTTCATCACATAATAAATTAGTCCCAATTCAAATTAAAGCAGGCACCAACAGTGTCATCCCCAGAGCCATCCACACCCTCGACAAACCCTCAGTGATTCTGTATGTTACTAAATTCACATGACACTGATGTGTATGATGTATGATTGGGTACCTGCAGTCTCAGTGTATCGCTGTATATAGTGTCAATAAAGGCATCATCAGCAGTAATAACTTCTCCTTCTTCTTTCAATTCTTCTTTCGGCTGCTCCCTTCAGGGGTCACCACAGCACATCATCTGCATCCATTTCATCCTTTACCTACCATCGTCTTGTCACATCAACCCTCTGGAGGCCTTCCTCCGGCCTGGCAGCTCCATTTTTAACAGCCTTAGTCCAGTATATCCATATCTCTGCTCTGCACATGTCCAAACTTTGTCTGGAGCAGTCCAGTCTTTTTGATCAGCAATAATAACCTATTACTCAAATATGTACTCGGCTAAACCATGGTCCACAGTACCACTGCACATAGCTGAAATGACAATAAAGCCACTTGACTTGGCAAAAATTATATAACTCAATTTTAATTTTTGGCATAAATTATATATATATATACAACAAAAGACAAACTGAAAAAAAATTATGCTAATTCTACACAAAACAAGACCGTCTGTACCAGGGGTACGCAATAAGTAAGTAAAATTTATTTATATAGCGCTTTTCACAGATAAAAAATCACAAAGTGCTTTACAGTACAAGAAATGGAAATATAAAACAATGTAACCGTAAATAAAAAAGCAAGGTAAAACAATAAAGAAAACAATAAAAACAACGTAAGAACAAATTAATCAAAAGCTTTTCTATATAAAAATGTCTTCAGCTATTTTTAAAAGATGTGCCCAATCTAAATCCCAATCTAAAACCCAATGTATTACTACTTAGATACTCTTTTGTGATATAAATGTTTTTATACTGAACGCGTTGTTTTATTGTTGTATATTTTATTACACTGTGAAGTGACGGTGCTGAGTAACTGCGTAACATTTCGACGGTAAACGTCAGAGTGACAGAACACGCCTCCGCCAAATACCGCAGCTCCCCAGCTTAGCCCAGCATACGGTGACGAGGAGAGGACAGAGGGAGCAGCTGGTGTGGACGCTGGCCGGATGGTTAAATCTAACCTACAGACGATTTTGAATAGTCACTGTTTTGCTAGAGAAAAAGAGAGAAACTTACCCGAGATGCCTGTTATCGAGAAGTCCAGTAATAAACCTGAAAGTGAAAGGTATGTTGTGGTCCGTGTCAGCTGCTCTCAGTGAGCTAAGCTAAGCTACGTTTAAAAACCGCGGCCTAGCTTTCGCATAAGCTGTGAGGCGACTAGCACAGCCCTTCATGTTTACATAACAGCGGCACTTATTTCAGTTGACATTTATAGAGAACATTATTAATCACGGGCAGCTTATTTCAATTCCATAAAAGATGCATATTAATCACCTTAAAATACATCGCAGTAGCGGGATTATGAGCAAAATGTTAGAAGTGACAGCGCTGCTTTGGTAGCTTCACAGATGCTAACGCGTCAGCTAATATGTCAAACGCCACAGGTTCGCGTTATCGATGGCTGGGCTAACGTGTCGTAAGGTTTTCTATTAAGACCTGGTAGCGACTAGAATTGTAATTTATAACATTTAAAATACAAATTTTGCGTGTTAAAAAGCGGAATAAGAGTGTTTTATCGTCACTAGGTGTGTTTTCCCCTTAAATATTAGCGTAGACATTTAAGCTAAATGCTAGCTCTTTAGCATTAGGTAATCTGACGGATGCGTTCATATTTTCGTCCTTCGCAATGAAAATGATCAATGGCTTTGAAACCTCGTTACTATCTTTTGTGATGTGGCTTTTGTGATGTAGAACTAGTACAAAAATATTTGCGCGGGGTTATGTAAAGTTAGCTAACAATGTAGTTAAAATGATGGCTGTACGGATTAATTCGTGGTTTCAGGGATTTTCTTATTTATTTATATATTTTATTTTATTTTTCCCTTTTCGCCCTTCTGAACCCACGATAAGACATCCAGTAACTTCTGGAAAACACGGGTTCCAGGTGGGCGGACAGGGAATACGTTTAACCGACGCTGAGAAGAACCTTTGTAATGTTTTTAATCAGATTCGGTTTGATTTATGCGAGCTGCTGCGGGGCCATTCTTCAGCCAGCCGTGTGCCTGTTTGGGTGCAGTTAAAGTACCGATTGACTGGAAGTGAGGGGGCTGGTAGGGGTGGATACCTCGAAACTGGTTGTTAGCGATTAACCCAATCTGGTGGTCGGTGGTTGCACCACTCGTGCCCTGACAGGATTTGTTGACGCGCTCTGTTTGTGCTGATCTATAGTGTTTGAGGCACTCATAGGAAATTTCCACTGCGGGGGAGGAGGGACGGAGGGGGGCAGGGTGGGTGCTGTATGGGGAATTTTGGAAGTTATGGCCTACACACAAATCTCGATGTATTTTTGCTTATCGTGGCAAGTAGTTGGTTGGAACTTGGATATAGTTATGTAATTGTCATGGTTAGATATTTATATATGTATATTATATAATAGTGTTTCTCTTTAAGCCAAAGCTTGAAAAGATGCCTTTATAAATATGCTGACACATAGCTATCATGTGTGATAAGCCTGGTTTAACATCCACACCTTCCAATAATAAAGCATGCATTAAAATAACATCATC19TATTATAGCAGCTAATAGCCTCCCCCCTCCCAAGGTAAAAAGTCTCTGTTACCTTAAGAGAAGGCTAGGCCTTTTCTTTTCATTCTTTTCATTTTTGACCACCTGATAAAATCAAATGTATTTAAACTTGTGTAAATTTAACCCCACATTAAAGACTTACATTTTTAACCTGCTAATTTTTCTTTTGTTGAAAGCCAGAACGTGTTAGGTGAGCTAAAATAAAACCCACATTGCAAATGTCATACTACATTGAAGTGTCTAACAGTGCAGGCTAGAACATGGGATTAAAGF25ATGATTCTCTGCTAGATGGATGCATGAGATTTATTGCAGATGTCCTTTACAATGATGTTCATTTACAGCAGAGTCTCCTACAAACACTACAAACACTATTCACAGCAGCTGTTTTTATTAAGATGACCGTCGGGGCGTCATTGCGR18CAGCACTGCCTACTGTTCACATGTCAGTCTCAAAGAATGCTAAAGGCAGAAACTGACTGAGAACTTTGTGTTCTCTTTAAATCGCAGAAAGGAGGCGTTAAGGGGTTCTGCAAGGATGGCAAGTGGCTTCCTTTCCTAATCTGATTACCGGATTTAGGGAGTAACTGCATTTGGTTAAAATCCCCAATCCTCTGTGAGATCATAAGTTGGATATTACACACTGCGAATGACACTGGTTTCTACACCTCTGCTCAACATGTAGCGCATTGGTGGCATTTGCGTAAGAGAGCATCCCTGTGTGTGATGGTGTTTGCGGCGTTGTTATTTTTGTTCCTAATAGAATCCCAGTGAACAGGCCTTCGCTCAAAGCTGTAAATGTATGCAGTCACAGCAAGCTAAATCTGAGGAGTGCTGTGACAGCGCTGAATTAGAACGGCTGATGAGTATTGTTTGTCCCAGTCTCTTAAACTGCAGTGACGCGGTGAGTGAAGAGCTAAAGTAGGCTAGCGGCTAGAGGAAAAAAATAGCCTGTTCTTCTGGCAGGTTTCCTGTGAGAACTGTGGAACAAGCTTAAAGTGCCCGCAGCAGAAGTGTGCAGAAGCCGTAGCCTCGATGTGTCTTTGTGAGACAAAAAACAAACAATTTCTTATAGTTGGTGGTCAATATTTGACTGACAGCTGACATAAAATTGTTAACAGTTTTAGTGAGGCATCAGTAATGTGGTGGTGTGACATAAACCACTTCTCCTTTCCGTTACGTGTTCCTCATCCGTCGGCACGTCATGCACGCATCTGTGCTCAATGTAGGAACGGTTATTGATTGAACCGGTTCCCCCAGTCTTGTGCTTTTGTGTCTGAAGACTCACTCTGGGTTAGTAAATGTTTTACTGCAGTATCACAGATGAGTCACTCGGCAGTTTAATGTGTGCCGAACCTGCTGCTTATAAAGAGTTTGGTTTGTGTTTGGTAGCAGAATCTCGACTGAATACCCAGCTCATGATGGCTCCATCTGTGTGTGCGTTTGTTTCGCTTTCCAGTATCTCCAGTCCTCTGAGGTGCTCCCGCTGTTGCAGTAACCCGTGTCCGGGGCCTCTGTGGTGCTCCTGATGCCCCTCTCCCACCCCTGAAGATCCCAGGTGGGCGAGGGAATGACCAACGGGATCGCAATCTTTCAGCTAAGCTGTT

***amhΔ-y*:**

GGAGTAGTGTTTTACAGTCTGGCTGTTGTCCAGCTCTGAGTGAACTTAAATATCGTGTTTGAATTTAAGGTGCTATCTAATCTGTTCATATGTAGTTTCTTTGTTCTGGTGCCAATTATAGCAACTGGGATTCAGCAAAATCAGGAAAAAAAAAAAAAAAGAATTATCATTTAACGACAGGATAAACCAGGGTCTACTTCCCCCCCAAAGTTTCACTTCATGAGTCACGCACACACACACTCAGACACACACACACAGCCAGTTCATTGAACTCTGCTCAGACGTCAAGCACCTTTGCTTTTGCGTGCATGCGTTATTTCCTCCCTCGGTGAAATTCTGTATTTGTTGCAATATATTTGGTTGCGTAACAAAATGAGGAACTTTTTTGGTCTAACTTCGCACTGGATATTAAGCTCACGTAGCAGAAGATGTACAAGCCCTTGCTTGTGTTAGTTGGCACCCCTGCCATGTTCGTGATTTACTGTATTTACTGTGTTTTCATATTTAAATTTGTGACAAACTGCAATGAGTTGTTGGTGCTTAATTCAAAGCCAAATAATAAATTAGATTGTGTCTGTTTGGCTTGCTCTGACTCCTGTCTCAAAAAATATTAAATATTTCCTTAAAGTTAAGTTGTTTCTGTTTTTCTGCTAATGTCAGATATTTAATGTTAGCGATAATCAGACAAATCTCTCAAAGGATGAGAGGAATGAGGAAAACATCTGCAGATGTTGATTTTGGCTTTATTTTGATGATAAGTTAAATTAATAACATGCCTGTGGGTGCCAATAATGCAATGCAGCGTCTTGTACATTAACATCTGTTTTAAAAAAAATGAAATGGTGCTCTTCAGCCACCCGTCACTGTGGCTGCTCTGTTAAGGTTCAGCGAACAGCTGAATCCACATGTCCAAGAACAACAGAGTTCAGTGAGTTTGCGTGTATTTATTAACCGTTTCTTTCCCGTCGGTTTGTGAAGTCACACAAACGGCAGCAGAAATATTCCTCTTTGTAAGCTAAGCATAACAGCAGAGATCCATGCATTCATATTTATCCGTGCTGTTAGGAAATGCAGGTTTAAATGAAAGTGGACTGATATGTGAAAATTTACTGCGTTTAGTTTAAACACACATACACACACACAGTCATGCATGCAAATCCTCACACACTGGGAAAACATAGTTGACCATAACCACAACAAACTACTGGACTTGTTTCACACCTCTTTGTTTGCTTTTTAAAAAACGGTGTAATTTGTTTTTGTAAACAATGTTTGTACTGTGAATGTGATTCATTCTCTGACTGTATAGTTGTGGATAATTTAGATGATTATATTTATGCTGTCGGTCTTTCCATTTGCCTTTTTTTTTTTTTTTATAATTtAGATGATTATATTTATGCTGTCGGTCTTTCCATTtGCCTTTTTTtgTTTtAAaTAAATCCTGAATTGTGTTGACATTGTAGAATGCATTAGTGTGTGTGTACTTGACTCACAGTCTGAAAACAATGGTGCTAAGTTTGGACTATGCCTGATCTTATTTATTCTGTAATGAAAACATCAAAAACAAGTCAAGTACTTGGTGCTGTACACAGGGACATTTGTAAAGCCGTTTTGGCATTTCCTTTCTCATATTTAATATGCTATTGTCTCGTTCTTTGAATGCATCGTGTACAACTGTAGACGCCACCTTtGGATGAAAGTGCTCTCGTGCAGCCGgCTCGGGCCcGAGCGACGGCCACGGGGCccTGTTAATGTTTAAACATACATCGGCCCGCTGTTGGTGAACAAGACACACTGTTTtGTACATAAGTTTTCTTtAATTGTACATCTCCATCATAAACAGAAAGGTATATAAATAAGTAACTATATACAGTCTATATAAaTATATATAAGTATATAAaTATATATACATATATATGTGAAAGGGgTGTTTGGTGCTGGCATTGTGACCCTTAAATTCACCCTGTAACAGGAGGCTTGAAGCTGAGCAGAGCAAAGCATTGCCACCAGAGGACCTTAACATGGCTGCACATCAGCACCTGAAACACACACACACACACACACACACACACACACACACACACACACACACACACACACACACACACATCCCCCGCAGCCACAAACTCAGAGGCTGTGTGTTTATTACACAAAGGTGCGGCGGCTCAAACGCCACCAGATTCCAGTACTAGAACTGTTATCACTTGTTTCAGGTATTTTCACTTCTTTGATTTATTGTTGGTTTTTTTGTTTGTTTTGGTGTTTAATTTTTACCAACCCAGATTCTGCAGGAAAATGGTGCTTCTGTTTCCAAGCGAGGGCAGTTCAGACCTTACAATCAAATCTTCACCGGTGCATACAGAACTGCGCGTCAGCCTCTTTACACACACCGCCTGTAGACGTCTTACTGACGAGGCCGTACCTCGCCGCGTGAGCTGATGTGAAACACTTGTCTTAGTGTGTGTGTGTGTGTGTGTGTGTGTGAGTGAGATCAGACAGGCTTCCTTTTTTCCTCTGATAGTGGGATTACACTAAAGCTCAGTTTGTACGTGTGTGTTTTTGTTTGGTTTTTAATTTTCACTTGCCAGCGCATATCTAAGGGAATATTAGTGTAATAATCAAAGCTTTATAGAAAATAAAACTCCTGTCCCGTGCACTCGTGTGCAAGTGAAGAGAAAAGTCATTTAGAATTAGACTAGTCCATTTGTTACCAGCACTGAGAGGTATTTGTAATTATTGTCGATGGTGTTTTGATTTCTTATTCTTGGTGTTTGATACGATTGCAGTTACATGTTATGCTTTGATTGTGTCTTCAGATTTCTGTCAGTTTTAATACTACTAGTCAGACAGCACGGTGCTCTCCTTCACTTAAAACAAGGAAATCGCAGCTATTGATGAATACTACTCTCCTCCTGTTGTGCTGCAGGGTTTATGGTTTAAGAATCTTACACACAAGCTCACCTGGTGGCTTTCTCACAGCCCAGTCTAACCTTTGAATCTTTAATCTGGAGGGACACCGGCGGGCGGAGGCGGGGGTGTACGTGCCTGTCAGAGCACTTTGAGGAAGGTGGGAGGTATTCCATTCATTACATACAGATTTAGGCAGTTTTATAGTTTTAAAGTATTTCTGGGGTGTGATTTGGCTTTAAAGCTGTGCAGTTCTGCATCATACTGCATCCTGGTACATTATTCGTAGATAATCTTTCAGAGCTTTGCAGTCAAGGCTGTTTACTCGAAATCCTGCCAAAGGTCTTAACTCAGGAACTCCCCACACTGCTTGAAAGAACAAGTTTCCTAATCTGCTGCATGTTACGTTAATTTACCTTCAGGTGGCATGACAAAGTCCCTCTACGACCCAACTCAGGACCAGGGCCACCCTTCAGTCAGTGTATTTCAAGGTAAATGTATCAGCTGCCCTCATCAGATCTCCTCAGACACAGTCCTGCCGGCACGTACACCCCCACCCCCGACAATCAGTCCGAGCTTTATGAGCGATCTGATCTCGAGAAGCTGTGAAATGGCAACGACTTTCAGCATCTTCCTCATCTCACAAATGCCTCTGTCTAGATTTGCGTCTCTAAGGAGGAGGTTCGGTGTCTTGTTTGAATCCCTCTTTGAATTGGGGGTAACATTCAAGGACACGCGTGACCTACAGGATGTGTATAGTGCTCTCTGATTGGTGAAGCCAGGGCAGGTTTGAGGTGCAATAATGGCAAACCTACTTCCAAAACGCCCTCGGTTTTCATGGAAACATTGTTACTGTTGATGTTGGTGCTTTTATCCTAAGATGTTATAATGAAAATGGAATTAATGTTTTTTCTCCCCTGCGATTTATGATAATTAATAAAATGTTTCTTATAATATTGAGTTTACGTTTGGAGTGAACTTTGTCTGAGTCCATTTGGTTTGCCGTGACGGAAGAGGGTGTCATGCTGATATCTTCATACCTGAACCATGTTTGGAGCCCCGCTACCTAAATCCTCAGTTAGTAGACTTCACAGGTAGGCGGCATTTACCTTGTAATGCCAAGTCGGCCCGGCAAAGACTCCAATCTGGCCCGCTCGACGGCAAATGTGAACTTCCACAAGTTTGATAGTTTTCCTAATGAAAAGACAAAGTAACCAACAAATATATAAACCCTTAAAGAAATTATTTTAATCTACTATAGCAGTTTGTTTTTACAGCTGGTCCACAGTATCTGTTGTGCTGACAGATTTCTTCAGTTCATTACAGCAGCACTTCTTAATTGAGTAGAAAACAGATATGTGGTTGAAACTGCACTTCCTGTTTCCACACGTCTCTGAGGTCAAATGTGTAGGTAAACCTCTTCACAGAGACAGAAAGGGAAGCTTCTCTGGTCCGGTTCACAAAAGTTCAAAGTTGGCTGCATGTGGCCTGCGATGTAAAATAAATTGATTAAAATGCGTCTCTGTATAAGATACTCGAGTTTTGAGAAGTCAGCATCATTAGTGATGGGTGTGTGTGTGTGTGTGTGTGTGTGTGTGTCAGCCCTGAGAAAGACTAACAGCCTGTTCACTGTGGTCCTGCCCTCCTGCAAGCCTGAATCAGAGAGATGGATATGAAATTGATGGATGATTAAAACCACAATTAATGACATTGTTTAAAAATATGTCAGCTTTAAAAGAAATGGAGAACGTTATAATAAACAATGTAGTCCTGCATAAGAGAGCTGCTTCAGTCACTAACTTGAGGATTAATGGTCCAGTGAGGTCTGTAAAACAAACAGCTTCTGCTGCTTCAGTTCACAGACGTGTTACCTTATTCTGTGTCGAATGCCTGTGATGCCTCAGAGAAAGCCTCCCAAAAGGCCCGTGCACTGAAGCTTTCCACCTGAAGCCAGGCCTGTGGCGTTCCCTGGTTACCGCCTTTGGCTTCGGGGTAATGAGGATACAACCTTACAACATGGACAGATGAGATTCCCCAGAAATGTATGTCTTCTGTGTTTTGTCCTTTTCCAACCTGGAAACTTAAGTGTGCTACTGTAGAGAGGTGAATACATGATGGGGAGAAACAGGAGTGTAGATGTTGATTACTTTTCATCAAAGTGCAATTCCTGGTTCCCACAAGTGTGACTGAAAACATCCAGCTGGTGTGCTTCAAACACCGCCTTGATACAATGAACATTTCCTGCCATTCCTCACTGATGATTTATAAGGTATGGTGAACCTTGGCTTTATCACCGGGGAAAACGTCCACAAATGGCTCCGAGACCTTGACTGTCTGCACAACAAGCCTGCAGCTGTGAGAATGCTGAGGGGAGTAACAGCTCCTCGAAGCCGCTCAGGTCTCCCTCTGACAACATGTTTCCTGCTCGTCTTCCAGCGTGAATGCTTCCAGATCTCCAGTGTGGGTTCATTAAGATAACCCATTCTCTTCCCATGACCTTTAATGGTAAGTTCACCCAAATGCAGGCAGACCACACAGTTGATGAACAATCTGATTCATTTCTCATCTGACATAGATGAAATAAATTAATGACATTTCAGTTATGAAACAAACTAAAGCGGATTGGAACTGTGAAATATTTTGCTCCCGAACAGTTTCTTTCCACAAAGCTTCTCTAAAATTAGAAAAGATATTAACTGAATGAAATTAAGCAAATAAACAAATTGCAGTATATTTGTACTTAAAACATTAATATAAAAGTGTTAAAGACTGCAGTCCTTATAAACCAGATTATTTCCTGGTTTGCAATAGTTACTTTTCTTATAATTTTAGAAATGCTAAAATGACTCTGGGTGCTGCTGTAATGTTTTATGAGCTGTAATAGTTAATTTTCAGTTTTAAATTGGACTCTGAAATAACGATTCAAAACTGCATTTGCAAACACTGGCTTTGAATTCATTGCACAT**C**TTGGCTTTTTCATTCAAAGACTTCATGGTTCAGTTGGTTTAACTGGTGAAGTACTGAAGGTTTCATGTTTTAGTTGATCAAATCAAATTGAGTCAGGAGTTCCTCAGGGATCTGTCCTTGGTCCGCTA**T**TCTTTAATATGCTCATAATATGTCTTATAAAACAGATCAGACTTTATTTAGAGTAGAATAAAGAGATGATAGTGGACACGAAGAAGAAGAGGAGGCCTCACCAGCCGCGGTTTATCCGGGGCCTTGAAGTGGAGAGGGTGAGCAGCTTTAGATACCTGGGCGTCTACATTTCTGTGGACATCACCTGGACACTGAACACCACACAGCTGGTCAAGAAGGCTCAGGAATGGCTGCATTTCCTGAGGAGGCTGAGGAAATTTGGTATGATCCTCAGCAGGTTCTACAGCTGCATTGTGGAGAGCACACTGACCAGCTGCATCACTGCATGATACGGCAGCACTACTGCCATGGACTGCAATCACCTGCAGAGAGTGATAACAACTGCAGGGAAGATAATCAGGACTCCGCTGCCCTCTCTGCACAGCATCTACCATCGCAGAGTCCAAAGGAGAGCTGCCTCCATCCTCAAAGACCCCACACATCCCCAACACGGACTGTTCACACTTCTGCCCTCGGGGCGGAGGTTTAGAAGTGTGAAATCCAGAACATCCAGACTCAACAACGCCTTCTTCCCCACTGCCATCAGACTCAAACAAGCTGACCTATTTTTGAACATCAATAATAATTTTTTGCACATCAGGTCATCCTGCATATTAAGCCAGCATATTAAGCTATAGCTCTTTTGCACACATCTCTGTTTCACACTTCAATAATTTGTCTCCTTTTCTGTCTTTATTTATTTGTGCTATTTGTATTTATCTGTTCTATTTCTACTGTAAATATTAACCGGCATCTGTGGGTGGACAGCAAAGCAAAAGTTTCATTGCACAGAGAAATGCCTTTTTTCTTTGGACACATGACAATAAACACTTTGAATCTTGAAATCAAAGAAATTTTCCATTATAAATAAATAAATGTGTAATATTACAGAAAAAAACTAATTTCTGGAGCAGATGGTGAAAAAAACAGGAACTTTTTTGTCATGTAATTGTTAATCACTTTGGTGTAGCATGAACATGTGGCAGAGTTCTTTATTAGCAGGAAGTTAAAAGTCCAAAATTTTTGCCATCCTTCAAGTGAGCTGGGTTGGAGTCATTGCTGAAATGCAAAGTGTTTCTTTAAAATCGTTTTGAGCTTTGTTGTCGACGTGATGGAAGCAAACCTCCAGTTCTACTGTCCTCTTTGTTTAACCCTCTCAGGCTCAAATTAAGTTTTAGTTACAGGAAAAATCTGATATTTGGGGAAAATTATCAAAAAAAAAAAGACTCATAAAATATGTATGTAGGGTAATCAGGTTGTTAGTTTTTAACTGTTGCAAATCGGCAACGCCTGCCTCGAGAGGGTTAAGTTAAGCAGCGTGCATATTAGCTCTGCAGCCACGAAACATTAAACAGCACTCTTCATCTTTCAGCCTGGTGTATTCTATGAAAGGTTTGTGAGAAACTTTCTTTCTGAGACCTGTTTGTGTCTGCTAGATAAGGATGTTCAGTGTGTCCAAAGTCGTCTTCTGTTTACCTCCGTGTCCCGTTTGCTGTCTGATTTTAGAATGTTCTCCACAAGCAGGTTAAAATACCTCAAATACTGACAATCTCAGTCATCAAGGACACACAGTGCTGCTATTTTGGCAGAAAGGGGACCCCGGGTGGTGAGAAGGGGAGGTCTGAGGATGTAACTCCCATCCTGTGAGAGATCTGACCACACAAACGCACGCGCAGACACTGCAAACATGTTGGGTCTGCTCGTTCTTTACAGCGAGGCGCTGACACTCTGCTGGACCCTGCAACCGGCCCAGGACCCCACAGTAACCGGTCAGACGCCGCTCAGCTGATTAATTAATAACTCGTTAACTTTGAATGGAAAAATGCATGCATGTGGTTATTGCTTCAGGCTGGTTCTTTAGTGCGTGAAGTAATTTGTCCGTTTCAGAGTACTCACTCCCATCAGCGAAGACCCCATCATCACCATCATCATCCTCAGCAGCAGCGCCTCATGCTGCACCATGCTTCGTGGAGGACATCTTTGCAGCGTTGCGTGAGGGTGTGGGGGACAGCGGCGAACTGACAAACAGCAGTTTGGTTCTGTTTGGATTCTGCTCGCAGTCTGCCCGCTCATCAGCCT**C**GGTCTCGTTAGACCTCGCTAACAAGAAGAGCAGCTTGGAGGTTCTGCACCCAGCTGCAGGTAAAAGCTGAAACTGCATTCCTGCTATTTTTCTTTCTTCATTTGTAAATTGTTTGGTTTCGTTCTTTTAGTACACGTATCAGAGGAAGAGGAGCAAGGAACAATCACGTTGACCTTTGACCTCCCACGGCCTCCTTCGCTCATGACAAACCCTGTGCTGCTCTTGGTCTTTGAAAGTCCACTGGCACGAGGAGACCTGGAAGTTGCTTTCACTAGTCAGTTTCTGCAGCCTAACACGCAGGTAAAGAGAGCTCAGGTGCAAACCCTGCTTCAAGGCTGCTGTCAAACATAAGGCCCGGGAGCCAGAATGGGCCCTGCAAAGACTCCACACTAGACGGCTTTGAGCTACTGGACGTTTTTCTGTAATTTTGCACATTTATTTCCTTCATTTATTACAGAAACCTTTCTCTGTCATGCACAAAAACTGAAGCTTGGTGTACAAATTGCACTTATTTTTTTTTTCATATTAAGATGTCTCAGTGATGAAATGTGCAGTTAAACTTGTCACACTCACCGAAAGAGGAGTTTCACTTAAGATCACAGCGAGCTGCATCAAGTGGCCCTCAATGTAAAACGAGGTTGCCATCCCTGCCTTAGGTGATAAAATGAAATGAAAGGTTTACAGATCTCTAACTCTGGGTTTTTGTGTTGCTTTATTTTCCAGGCTGTGTGCATTTCAGGAGACACACAGTACGTACTGCTGACAGGAAAATCATCAGAGGGGAGTGTTAATGACAGGTGGCAGATTACGGCTCAGACAAAACTCCCTCATATGAGTGAGCTATCATCTTCTTCATTTTATTTCCCCATCTCTGGTTCATTGTGTACCCTACTTACATTTCCTCTCATTGTAGAGCAAAACCTAAAAAGCATCTTGATTGGTGAAAAATCAGGAAGTAACATCAGCATGAGTCCACTTCTACTTTTCTCCGGGGGAACGGGAACTGATACGAGGTCAGCCCGGCTTTCTTTCTGCTGACATTCACTGTCGTCAGAGACGCGCTCAGCTTTGGTTTGTATTTTTGCTTTCCCAGATGTGCTTCAGGCTCGCCCCCGGCATCTCTGCAAACCTCCTTCCTTTGTGAATGTCGATGAAACGCTTCCTGGGTGCTGTTCT**G**CCTCAGGAACACTTCGCGTCCCCTCCACTTCCTCTGGACTCCTTACAGTCTCTGCCTCCCCTCTCGCTTAGCTTATCCTCCAGCGAGACCCTGCTGGCAGTAATGATCAACTCCACAGCTCCCACAGTCTTTGGCTTCACGAGCTGGGGCTCCGTGTTGCCGGTGTGCCACGGAGAGCTGGCCCTGTCTGCTGCACTGTTAGAGGAGCTCAGACAGAGACTGGACCAGACTTTGGTGCAAATGACAGAAATAATCAGAGAGGAAGAGATTTCACCGGGAACCAAGGAGAGCCTGGGGAGGCTCAAAGAACTGAGTGCGTTACAGAAGAAAGAACATGCCACAGGTGACATGTGCAAGAACAGTTACAGTTACATAGGAAGCATGTTTTTTTTTAAATGAAACAACACC**C**TGCAGCACGGTC**G**GGTGCATTTAAGTATCCCGCTGCAGCTTTCAGCTCCCAGCTTTAGGAGATGTTGGGTGGAGGGAGTGGCCACGGATTCTCATTTATAACAGGCCTTACAGTCAAAGGTTTATGGGCCGAAAACAGTAATCATGCTGCAGACTGTCTGTTCAGTTCTACAGCGGTCCCAGTGACCTATGAGTTCAGATTATTTTAAGTCTTGTGCACAACAGATTCCCATTATTCAAATTTTAAAAGTTCTGAAGTCGGCAGATTAAAAAAATAAATAAATTAGATCTCATTCATCAAAAAACTAAAAACCTGGATGTGAAATATTTAGATAGATATCAGCAGATGTCTTTAAGAAAACTAATCATGTGAGTGTGTGTGAGCTTGGCTGATGTTCCAAAAGGCTTCGTTTCTATTATTATATCATTTGCTGTTTTGTCAGTGGTAAAATTTGGGTCTATATTACCCACTGACCTTTGACATATATAGATGAGCAACTATATTAAATTTAATTTTATTAGATTTTGTTATTTTGTCATTTTCCTCTGCTGTGTTCATCAGTTTATCAGCTCCACAGACTCTACAAACTGGGACTCATTTCCACTTTTGCTTTTTGCTTAGCGTGAATTTTGTCCTCACCGCTCTAAAGGTCACGTATATCCCTTTTTAAGTGCTGCATGTGTTTGCTTTCTGCGTCCGCCAGGAGGGAGTCAGTTCCGTGCGTTTCTTCTGCTGAAGGCTCTGCAGACGGTGGCCCAAACGTACGACGCGCAAAGAAAACTGCGGGCCACCAGAGCAGACCCCAGTTCGTCAGTGAGGGGCGGCGTCTGTGGGCTGAAGGCTCTCACCGGTTGTGAGGGAGTGTGGATGCCGCTAGAGCTGCTCTCTTCTGCTACTTTACCCCAGCATTTATAACTTTCACACAACTGATTAGTTATGCGCATGTTTCAGTTATGAAATATCAATTACAACACACCACGTGTACTTCTAATTAAATATAACTGCTTTTATGCTTTATTAAAGATCACAAGTCTTAAGATCACAAAGTTAATGTTAATTCTATTTATGTTAAGATATTTTGATAATTAGTGATGCTAAAATGAATCAGATTTTGTCATTTTGAAAATATTTCTAATTGGAATAATGTTTCTGAGTGTTAAAGTTAAACTGTTAAAAGGATGAAATATAGCCTTCTCAAACTTGTAATAATTAAATGCAAAGTGTACTTAAAGTTCTGTGTTCGTAGCCATTCAGCTACATAAGCTACAGTATATGTTTTATGCTTTGATTTTGGGTTTCAGGATTGCTTTATTAGCAGCTGGCTTCTTCCTCCTTCTTATCGCTCCGACTTCTTCAGATCCCCACCACCGGGTGAAGCCGTCCCATATGGAAACCTGGAGGCAGACAATGGGACACAAGACACGTGTGATTCCATATCAACCAGCCCTAAATACTGTGGAAATCCTATAGTGTTGCATAATAAAAATTCTCATATATCATAATATTTGAGCATGTGATATTTAAAGAGATGATCCAAAGTCCAGTTTATTTTTGGTCCTCTGAGCTGTAGAGCTGTTTGTCCAGCTTGAGCAGTTTAGTGTGCATTTCCTAGACTTGGAGAAACCGGCTGTAGACATTTCTGCGCTCTTTGGAACATTTATGAAAATTATTATTGCTATATTATAAAATTATTAAGCACACATCTTGCAGTACCAAAAATACGATAATGACTCTGTTACTCTCAAAGATAGCTGTGAAAAGTTCGGCATCCATCACCCAAAGAAGTATACACTAACCTGTGATAGCCAAGGTTATTACTGTTAACTAATACTAACTTGTTACGCCGCTCTGCAGCCCCTAATCTCTTTGCAGTGGCAGTGCAAGAGTGTTCAGCTGTTGCTAATTGACCACACTTAATCAGGTGTGGATAAAGGCATACTTGAGGCAGGCTTTCATGGAGGCGCACTAGTCTTTGTCTTAGTGGTACCAGCTATTTTAGCTAACTTATTATATAATTTAAAATAAAACCTCTTCTCTGTGAAATTCTGGTGTTGGTCTCCTTAATTATGTTGCAGCTTTTGAGCTGGCTCGTAACATAACTAAAATTAAAATATAGAGGAAATATTGTGGAGTTTTAGCATCTGTTAATCTCGTTTAACCTGTCAGCTGATGAGGTTTTGATAAACGTGTTTGAGACTTTGGATCTCTACCAGACAAAAACTTATAGTTTGATTGAAATATATGCTCTGCTTTTTCTAAATCTTACCTCTGACATACAATAAAAGGTTAAAACTAACACTGCACCGAATAAAAACTAAACTAAACATTTTCGAGCAGTAAAAACAAAAAAGTCAAAATAAAATAAAAACTTATTAGAGAATACAAAAAACTATAATAACTCTGGTGACAGGATTTAATCGTGGCGCCACCCTTCAGCTGAGCTACAGCAAGCTGCAAACCTCTTTTGGTTGGTATTTGTAATTCTCTCTGTTGTGAGCATGAAACCGCTCACAACAGTCTCATTGAGGCATACATCTCCAAAAATGGGAAAATCACACCAAAATAGTCCAGATGGGTGCACAGATGATGTCATCTAGACACAGGATGCACTCTGATGTCTTTAACGCTTAAAAACATGTGGAAGAATCAGAAACGTGATCTTGATTGTACAACATAAAAATCTTGATGTATGTAAAATAAGACACACTATAAGATACACTTCTAATGTTGTTCTTTTAACACACAGAAGCTCCAAATGCTGTTCACTGCTCTAGTCCACAGGATCACCAGGCAGCTCTGCTCAGTACACCAACACCTGCACCGTACACAGTGTTTCATGCTTTATGCTTTGGGTTGAACAGCTACGGCTATTGTCAATCTTGCAAAACTTTATTTTTTCATCCATGAGAGGGTAGGTTTGCTCATTCAGTCTTTAAAAATGGGAATAAAATTGTTTAAACCAGTAAGATTACATGTAAGGAGATATGGAGCCATCCAGTGGTTCATCAGCCGTGTGTTGACCGGGTATTTGTTAATTCTCAGCCATACAAACGGACACTTTACGGTTTCCTTATTTCCAAATCTGACAAAGACTTTCTCAAAATCTCTGTGAACTTACAGCCGGCCCATTATTCTCCATCTCACAGTGTTTATCCTACTGTTGTTGCTTTATGATGCCAGGACAATATGGCCCTCTCAGACTGATAGCTTGTCACTGAGATGCTTTGAACAGCAAGAGAAGAAGATGGATTACGGCTTTAAAGGCAGAAAAGCTAAAATGGCTCATTTCAGACAGCGGATGAACTGAAGGGTTGCGCTCATTAAAGAATAATGTTTAAGATATATGATTAAAAGTCCAAGTACTAAAATACCGAGCTGGAAATGAGCACGACTGTGTATTCAGCCAGAAAACAGAGTAAAAAACTTTACTGTATCTGTAAATTACACATAAAATCACCCTCAGTCTTTAAACAAGAACAAATGAACCGAAAGTTGCAGGCTAACTGACAGAATCACAACGAGAAACCATACCTGTGTTATTGAAACACTCTGCAGACTTCCAAAGAAAGACCACACACCTGTCTCTTCAACAAAATAGTCCAGAGCATCTAGAAAACACAATTGCACAAAAGACTGAGAGCGTGTTAAACAGAAGCTGTCGGCTGTTACAAATCACAGCAATCAGTCAGAAACAAGCTCAAAACATGTGTGATGTGGGTAAAGAGACAGTGCACCGGCACTCACCGTGCACTTCGTTAACTCCTGAGCTCACCAGTAGCACAACCAGCGCCGCCACTAAACAGCGGTTCATAGTGAATCCTTTCCACGGATTTGGAACATCGCTCAGATTTTCAATGCACAGAATCCGCTCATAAGACACTGCAGACAGACACGAGTCAGACGCATTTAGTCCATGCTCAGTTTCATCAGTCTGGTGGCTGTTTAGTGAAACGTGTGTCTTACTCGTGAAGCCTTCAGACAGGCCTGCTCCAGCTTCTTAAGGCTGAAAAATAACACTGCAAGATGAGGAAAGATTGGGTAGGACTGTGTGGTTGAGATTTAGTAAAGTAGCAGTACACAGAATACACAGAATGATCACACGTGGAGTTTGTTTAATCCCCGATATCTTATATCTGTGATCATATCCCACTTTCCCCTGTACTGCCTTTTGCACCCTCTTCCAAGCCATACACTAACATGTGACACTAACACAGCTTAAAATAGTCACATGCAGCTCTTTAGAAAGACAAATATGGAGCTGAGCGTTTAGTTTGATACAAACCTCTGGACAGAAAAATACGCTTACCTGATTTTCAGTTGTGCTTAACCACAGAGGTGAGGGATAGTAGAGCTGTGCTGTTGCGGTTGTGTACACGCTCTGCACCTCGCTTCTTTGCTTGATTGACAGGCACAGGTGTGAAAACAGGGAAAGGGACATTCAAACTTAATAGCAGTAAGCCAGTAGGTGAACGAGGATCAGCAACACCCGCCTTCCAGTGCCATGAACACTCCTACTCCTCCACAGAGGCAGATAAGCCAGCGTTACTAGGGTGGTTTAACAGTTTACTTAATTATGAGGAGGTCCTAAAACACAATACATTGTTAAAAGTTCATCACATAATAAATTAGTCCCAATTCAAATTAAAGCAGGCACCAACAGTGTCATCCCCAGAGCCATCCACACCCTCGACAAACCCTCAGTGATTCTGTATGTTACTAAATTCACATGACACTGATGTGTATGATGTATGATTGGGTACCTGCAGTCTCAGTGTATCGCTGTATATAGTGTCAATAAAGGCATCATCAGCAGTAATAACTTCTCCTTCTTCTTTCAATTCTTCTTTCGGCTGCTCCCTTCAGGGGTCACCACAGCACATCATCTGCATCCATTTCATCCTTTACCTACCATCGTCTTGTCACATCAACCCTCTGGAGGCCTTCCTCCGGCCTGGCAGCTCCATTTTTAACAGCCTTAGTCCAGTATATCCATATCTCTGCTCTGCACATGTCCAAACTTTGTCTGGAGCAGTCCAGTCTTTTTGATCAGCAATAATAACCTATTACTCAAATATGTACTCGGCTAAACCATGGTCCACAGTACCACTGCACATAGCTGAAATGACAATAAAGCCACTTGACTTGGCAAAAATTATATAACTCAATTTTAATTTTTGGCATAAATTATATATATATATACAACAAAAGACAAACTGAAAAAAAATTATGCTAATTCTACACAAAACAAGACCGTCTGTACCAGGGGTACGCAATAAGTAAGTAAAATTTATTTATATAGCGCTTTTCACAGATAAAAAATCACAAAGTGCTTTACAGTACAAGAAATGGAAATATAAAACAATGTAACCGTAAATAAAAAAGCAAGGTAAAACAATAAAGAAAACAATAAAAACAACGTAAGAACAAATTAATCAAAAGCTTTTCTATATAAAAATGTCTTCAGCTATTTTTAAAAGATGTGCCCAATCTAAATCCCAATCTAAAACCCAATGTATTACTACTTAGATACTCTTTTGTGATATAAATGTTTTTATACTGAACGCGTTGTTTTATTGTTGTATATTTTATTACACTGTGAAGTGACGGTGCTGAGTAACTGCGTAACATTTCGACGGTAAACGTCAGAGTGACAGAACACGCCTCCGCCAAATACCGCAGCTCCCCAGCTTAGCCCAGCATACGGTGACGAGGAGAGGACAGAGGGAGCAGCTGGTGTGGACGCTGGCCGGATGGTTAAATCTAACCTACAGACGATTTTGAATAGTCACTGTTTTGCTAGAGAAAAAGAGAGAAACTTACCCGAGATGCCTGTTATCGAGAAGTCCAGTAATAAACCTGAAAGTGAAAGGTATGTTGTGGTCCGTGTCAGCTGCTCTCAGTGAGCTAAGCTAAGCTACGTTTAAAAACCGCGGCCTAGCTTTCGCATAAGCTGTGAGGCGACTAGCACAGCCCTTCATGTTTACATAACAGCGGCACTTATTTCAGTTGACATTTATAGAGAACATTATTAATCACGGGCAGCTTATTTCAATTCCATAAAAGATGCATATTAATCACCTTAAAATACATCGCAGTAGCGGGATTATGAGCAAAATGTTAGAAGTGACAGCGCTGCTTTGGTAGCTTCACAGATGCTAACGCGTCAGCTAATATGTCAAACGCCACAGGTTCGCGTTATCGATGGCTGGGCTAACGTGTCGTAAGGTTTTCTATTAAGACCTGGTAGCGACTAGAATTGTAATTTATAACATTTAAAATACAAATTTTGCGTGTTAAAAAGCGGAATAAGAGTGTTTTATCGTCACTAGGTGTGTTTTCCCCTTAAATATTAGCGTAGACATTTAAGCTAAATGCTAGCTCTTTAGCATTAGGTAATCTGACGGATGCGTTCATATTTTCGTCCTTCGCAATGAAAATGATCAATGGCTTTGAAACCTCGTTACTATCTTTTGTGATGTGGCTTTTGTGATGTAGAACTAGTACAAAAATATTTGCGCGGGGTTATGTAAAGTTAGCTAACAATGTAGTTAAAATGATGGCTGTACGGATTAATTCGTGGTTTCAGGGATTTTCTTATTTATTTATATATTTTATTTTATTTTTCCCTTTTCGCCCTTCTGAACCCACGATAAGACATCCAGTAACTTCTGGAAAACACGGGTTCCAGGTGGGCGGACAGGGAATACGTTTAACCGACGCTGAGAAGAACCTTTGTAATGTTTTTAATCAGATTCGGTTTGATTTATGCGAGCTGCTGCGGGGCCATTCTTCAGCCAGCCGTGTGCCTGTTTGGGTGCAGTTAAAGTACCGATTGACTGGAAGTGAGGGGGCTGGTAGGGGTGGATACCTCGAAACTGGTTGTTAGCGATTAACCCAATCTGGTGGTCGGTGGTTGCACCACTCGTGCCCTGACAGGATTTGTTGACGCGCTCTGTTTGTGCTGATCTATAGTGTTTGAGGCACTCATAGGAAATTTCCACTGCGGGGGAGGAGGGACGGAGGGGGGCAGGGTGGGTGCTGTATGGGGAATTTTGGAAGTTATGGCCTACACACAAATCTCGATGTATTTTTGCTTATCGTGGCAAGTAGTTGGTTGGAACTTGGATATAGTTATGTAATTGTCATGGTTAGATATTTATATATGTATATTATATAATAGTGTTTCTCTTTAAGCCAAAGCTTGAAAAGATGCCTTTATAAATATGCTGACACATAGCTATCATGTGTGATAAGCCTGGTTTAACATCCACACCTTCCAATAATAAAGCATGCATTAAAATAACATCATC19TATTATAGCAGCTAATAGCCTCCCCCCTCCCAAGGTAAAAAGTCTCTGTTACCTTAAGAGAAGGCTAGGCCTTTTCTTTTCATTCTTTTCATTTTTGACCACCTGATAAAATCAAATGTATTTAAACTTGTGTAAATTTAACCCCACATTAAAGACTTACATTTTTAACCTGCTAATTTTTCTTTTGTTGAAAGCCAGAACGTGTTAGGTGAGCTAAAATAAAACCCACATTGCAAATGTCATACTACATTGAAGTGTCTAACAGTGCAGGCTAGAACATGGGATTAAAGF25ATGATTCTCTGCTAGATGGATGCATGAGATTTATTGCAGATGTCCTTTACAATGATGTTCATTTACAGCAGAGTCTCCTACAAACACTACAAACACTATTCACAGCAGCTGTTTTTATTAAGATGACCGTCGGGGCGTCATTGCGR18CAGCACTGCCTACTGTTCACATGTCAGTCTCAAAGAATGCTAAAGGCAGAAACTGACTGAGAACTTTGTGTTCTCTTTAAATCGCAGAAAGGAGGCGTTAAGGGGTTCTGCAAGGATGGCAAGTGGCTTCCTTTCCTAATCTGATTACCGGATTTAGGGAGTAACTGCATTTGGTTAAAATCCCCAATCCTCTGTGAGATCATAAGTTGGATATTACACACTGCGAATGACACTGGTTTCTACACCTCTGCTCAACATGTAGCGCATTGGTGGCATTTGCGTAAGAGAGCATCCCTGTGTGTGATGGTGTTTGCGGCGTTGTTATTTTTGTTCCTAATAGAATCCCAGTGAACAGGCCTTCGCTCAAAGCTGTAAATGTATGCAGTCACAGCAAGCTAAATCTGAGGAGTGCTGTGACAGCGCTGAATTAGAACGGCTGATGAGTATTGTTTGTCCCAGTCTCTTAAACTGCAGTGACGCGGTGAGTGAAGAGCTAAAGTAGGCTAGCGGCTAGAGGAAAAAAATAGCCTGTTCTTCTGGCAGGTTTCCTGTGAGAACTGTGGAACAAGCTTAAAGTGCCCGCAGCAGAAGTGTGCAGAAGCCGTAGCCTCGATGTGTCTTTGTGAGACAAAAAACAAACAATTTCTTATAGTTGGTGGTCAATATTTGACTGACAGCTGACATAAAATTGTTAACAGTTTTAGTGAGGCATCAGTAATGTGGTGGTGTGACATAAACCACTTCTCCTTTCCGTTACGTGTTCCTCATCCGTCGGCACGTCATGCACGCATCTGTGCTCAATGTAGGAACGGTTATTGATTGAACCGGTTCCCCCAGTCTTGTGCTTTTGTGTCTGAAGACTCACTCTGGGTTAGTAAATGTTTTACTGCAGTATCACAGATGAGTCACTCGGCAGTTTAATGTGTGCCGAACCTGCTGCTTATAAAGAGTTTGGTTTGTGTTTGGTAGCAGAATCTCGACTGAATACCCAGCTCATGATGGCTCCATCTGTGTGTGCGTTTGTTTCGCTTTCCAGTATCTCCAGTCCTCTGAGGTGCTCCCGCTGTTGCAGTAACCCGTGTCCGGGGCCTCTGTGGTGCTCCTGATGCCCCTCTCCCACCCCTGAAGATCCCAGGTGGGCGAGGGAATGACCAACGGGATCGCAATCTTTCAGCTAAGCTGTT

***amhy*:**

TACACCACCTCCCAGCTCCCCAGCTCCCGACCTAGATAGGGTTTGGGGCTGAGGGGAAGGGGAGAGGAGAGGGGAAGGGCGGAGAACAATCTGAGGGTGGATAGAGGGTGAGCGAGAGAGGCTGGTAGGGAGTGAGAGAGACAGGATGCCTGTTTTCCATGTGCTTCTGCAAGCTGTTTGTCTGATTGAACAAGGTAAGAGAAGTAGGTGTTAGATTAAAAGTAAAGACTTTGATTTTGAACTTGTTAGGGTTTAGATCCTCAATCAGCTGTCTGTGTTTTTGCTGTGTTATTTCTGGAAGCCTTAGAATCACTCATGATGTCCTCGTCTGTCTTTGAATGTTTCAGGTAATTGATGAAAACTACCTCAACATTAAATAAACTATGCAAATGTCCAGGTGTGGACCAAGGCACTCTCCTCATTCACTGGTGCTTCAAACTCTGCACTACCCAATCGCCTCCAAGACCGGCTTCTGTACTGGGATCTTTGTCTCTCTCTCACACACACACACACACACACACACACACACACACACACACACACACAACACACACACTACACCACTAGTGCACCTGACGCAGAATTCATAGGTACTGGTCATGTTTGTGTGTGGAGTAGTGTTTTACAGTCTGGCTGTTGTCCAGCTCTGAGTGAACTTAAATATCGTGTTTGAATTTAAGGTGCTATCTAATCTGTTCATATGTAGTTTCTTTGTTCTGGTGCCAATTATAGCAACTGGGATTCAGCAAAATCAGGAAAAAAAAAAAAAAAGAATTATCATTTAACGACAGGATAAACCAGGGTCTACTTCCCCCCCAAAGTTTCACTTCATGAGTCACGCACACACACACTCAGACACACACACACAGCCAGTTCATTGAACTCTGCTCAGACGTCAAGCACCTTTGCTTTTGCGTGCATGCGTTATTTCCTCCCTCGGTGAAATTCTGTATTTGTTGCAATATATTTGGTTGCGTAACAAAATGAGGAACTTTTTTGGTCTAACTTCGCACTGGATATTAAGCTCACGTAGCAGAAGATGTACAAGCCCTTGCTTGTGTTAGTTGGCACCCCTGCCATGTTCGTGATTTACTGTATTTACTGTGTTTTCATATTTAAATTTGTGACAAACTGCAATGAGTTGTTGGTGCTTAATTCAAAGCCAAATAATAAATTAGATTGTGTCTGTTTGGCTTGCTCTGACTCCTGTCTCAAAAAATATTAAATATTTCCTTAAAGTTAAGTTGTTTCTGTTTTTCTGCTAATGTCAGATATTTAATGTTAGCGATAATCAGACAAATCTCTCAAAGGATGAGAGGAATGAGGAAAACATCTGCAGATGTTGATTTTGGCTTTATTTTGATGATAAGTTAAATTAATAACATGCCTGTGGGTGCCAATAATGCAATGCAGCGTCTTGTACATTAACATCTGTTTTAAAAAAAATGAAATGGTGCTCTTCAGCCACCCGTCACTGTGGCTGCTCTGTTAAGGTTCAGCGAACAGCTGAATCCACATGTCCAAGAACAACAGAGTTCAGTGAGTTTGCGTGTATTTATTAACCGTTTCTTTCCCGTCGGTTTGTGAAGTCACACAAACGGCAGCAGAAATATTCCTCTTTGTAAGCTAAGCATAACAGCAGAGATCCATGCATTCATATTTATCCGTGCTGTTAGGAAATGCAGGTTTAAATGAAAGTGGACTGATATGTGAAAATTTACTGCGTTTAGTTTAAACACACATACACACACACAGTCATGCATGCAAATCCTCACACACTGGGAAAACATAGTTGACCATAACCACAACAAACTACTGGACTTGTTTCACACCTCTTTGTTTGCTTTTTAAAAAACGGTGTAATTTGTTTTTGTAAACAATGTTTGTACTGTGAATGTGATTCATTCTCTGACTGTATAGTTGTGGATAATTTAGATGATTATATTTATGCTGTCGGTCTTTCCATTTGCCTTTTTTTTTTTTTTTATAATTtAGATGATTATATTTATGCTGTCGGTCTTTCCATTtGCCTTTTTTtgTTTtAAaTAAATCCTGAATTGTGTTGACATTGTAGAATGCATTAGTGTGTGTGTACTTGACTCACAGTCTGAAAACAATGGTGCTAAGTTTGGACTATGCCTGATCTTATTTATTCTGTAATGAAAACATCAAAAACAAGTCAAGTACTTGGTGCTGTACACAGGGACATTTGTAAAGCCGTTTTTTGGCATTTCCTTTCTCATATTTAATATGCTATTGTCTCGTTCTTTGAATGCATCGTGTACAACTGTAGACGCCACCTTTGGATGAAAGTGCTCTCGTGCAGCCGGCTCGGGGCCGACGACGGCCACGGGGGCCTGTTAATGTTTTAACATACATCGGCCCGCTGTTGGTGAACAAGACACACTGTTTTGTACATAAGTTTTCTTTAATTGTACATCTCCGTCATAAACAGAAGGTATGTAAATAAGTAACTATATACAGTCTATATAAATATATATAAGTATATAAATATATATACATATATATGTTGAAAGGGGTGTTTTGGTGCTGGCAAATTTATGCCATCCTTCAAGTGAGCTGGGTTGGAGTCATTGCTGGGTGATTCTAGCCCCTGGGCCTGATGTTGGACATCCCCCGCTTAGAGAAACAGAAAGATGAAAAACAGTGGATGTGGCTGAAGTTTCCCTTCAAGGCTTTCTGCCAGCAGTCTCTCTAAGAGGAAGAGTCATCAGTCCAAAGCAACAAGTAAGCAGATTTACCAAAATGCAAAGTGTTTCTTTAAAATCGTTTTGAGCTTTGTTGTCGACGTGATGGAAGCAAACCTCCAGTTTTACTGTCCTCTTTGTTTAACCCTCTCAGGCTCAAATTAAGTTTTAGTTACAGGAAAAATCTGATATTTGGGGAAAATTATCAAAAAAAAAAAGACTCATAAAATATGTATGTAGGGTAATCAGGTTGTTAGTTTTTAACTGTTGCAAATCGGCAACGCCTGCCTCGAGAGGGTTAAGTTAAGCAGCGTGCATATTAGCTCTGCAGCCACGAAACATTAAACAGCACTCTTCATCTTTCAGCCTGGTGTATTCTATGAAAGGTTCGTGAGAAACTTTCTTTCCGGGACCTGTTTGTGTCTGCTAGATAAGGATGTTCAGTGTGTCCAAAGTCGTCTTCTGTTTACCTCCGTGTCCCGTTTGCTGTCTGATTTTAGAATGTTCTCCACAAGCAGGTTAAAATACCTCAAATACTGACAATCTCAGTCATCAAGGACACACAGTGCTGCTATTTTGGCAGAAAGGGGACCCCAGGTGGTGAGAAGGGGAGGTCTGAGGATGTAACTCCCATCCTGTGAGAGATCTGACCACACAGACGCACGCGCAGACACTGCAAACATGTTGGGTCTGCTCGTTCTTTACAGCGAGGCGCTGACACTCTGCTGGACCCTGCAACCGGCCCAGGACCCCACAGTAACCGGTCAGACGCCGCTCAGCTGATTAATTAATAACTCGTTAACTTTGAATGGAAAAATGCATGCATGTGGTTATTGCTTCAGACTGGTTCTTTAGTGCGTGAAGTAATTTGTCCGTTTCAGAGTACTCACTCCCATCAGCGAAGACCCCATCATCACCATCATCATCCTCAGCAGCAGCGCCTCATGCTGCACCATGCTTCGTGGAGGACATCTTTGCAGCGTTGCGTGATGGTGTGGGGGACAGCGGCGAACTGACAAACAGCAGTTTGGTTCTGTTTGGATTCTGCTCGCAGTCTGCCCGCTCATCAGCCTTGGTCTCGTTAGACCTCGCTAACAAGAAGAGCAGCTTGGAGGTTCTGCACCCAGCTGCAGGTAAAAGCTGAAACCGCATTCCTGCTATTTTTCTTTCTTCATTTGTAAATTGTTTGGTTTCGTTCTTTTAGTACACGTATCAGAGGAAGAGGAGCAAGGAACAATCACGTTGACCTTTGACCTCCCACGGCCTCCATCGCTCATGACAAACCCTGTGCTGCTCTTGGTCTTTGAAAATCCACTGGCACGAGGAGACCTGGAAGTTGCTTTCACTAGTCAGTTTCTGCAGCCTAACACGCAGGTAAAGAGAGCTCAGGTGCAAACCCTGCTTCAAGGCTGCTGTCAAACATAAGGCCCGGGAGCCAGAATGGGCCCCGCAAAGACTCCACACTAGACGGCTTTGAGCTACTGGACGTTTTTCTGTAATTTTGCACATTTATTTCCTTCATTTATTACAGAAACCTTTCTCTGTCATGCACAAAAACTGAAGCTTGGTGTACAAATTGCACTTGTTTTTTTTTTCATATTAAGATGTCTCAGTGATGAAATGTGCAGTTAAACTTGTCACACTCACCGAAAGAGGAGTTTCACTTAAGATCACAGCGAGCTGCATCAAGTGGCCCTCAATGTAAAACGAGGTTGCCATCCCTGCCTTAGGTGATAAAATGAAATGAAAGGTTTACAGATCTCTAACTCTGGGTTTTTGTGTTGCTTTGTTTTCCAGGCTGTGTGCATTTCAGGAGACACACAGTACGTACTGCTGACAGGAAAATCATCAGAGGGGAGTGTTAATGACAGGTGGCAGATTACGGCTCAGACAAAACTCCCTCATATGAGTGAGCTATCATCTTCTTCATTTTATTTCCCCATCTCTGGTTCATTGTGTACCCTACTTACATTTCCTCTCATTGTAGAGCAAAACCTAAAAAGCATCTTGATTGGTGAAAAATCAGGAAGTAACATCAGCATGAGTCCACTTCTACTTTTCTCCGGCGGAACGGGAACTGATACGAGGTCAGCCCGGCTTTCTTTCTGCTGATATTCACTGTCATCAGAGACGCGCTCAGCTTTGGTTTTTATTTTTGCTTTCCCAGATGTGCTTCAGGCTCGCCCCCGGCATCTCTGCAAACCTCCTTCCTTTGTGAGATGAAACGCTTCCTGGGTGCTGTTCTCCCTCAGGAACACTTCACGTCCCCTCCACTTCCTCTGGACTCCTTACAGTCTCTGCCTCCCCTCTCGCTTGGCTTATCCTCCAGCGAGACCCTGCTGGCAGTAATGATCAACTCCACAGCTCCCACAGTCTTTGGCTTCACGAGCTGGGGCTCCGTGTTGCCGGTGTGCCACGGAGAGCTGGCCCTGTCTGCTGCACTGTTAGAGGAGCTCAGACAGAGACTGGACCAGACTTTGGTGCAAATGACAGAAATAATCAGAGAGGAAGAGGTTTCACTGGGAGCCAAGGAGAGCCTGGGGAGGCTCAAAGAACTGAGTGCGTTACAGGAGAAAGAACATGCCACAGGTGACATGTGCAAGAACAGTTACAGTTACATAGGAAGCATGTTTTTTTTTAAATGAAACAACACC**C**TGCAGCACGGTC**G**GGTGCATGTAAGTATCCCGCTGCAGCTTTCAGCTCCCAGCTTTAGGAGATGTTGGGTGGAGGGAGTGGCCACGGATTCTCTAAACCCCATTTATAACAGGCCTTACAGTCAAAGGTTTATGGGCCGAAAACAGTAATCATGCTGCAGACTGTCTGTTCAGTTCTACAGCGGTCCCAGTGACCTATGAGTTCAGATTATTTTAAGTCTTGTGCACAAACAGATTCCCATTATTCAAATTTTAAAAGTTCTGAAGTCGGCAGATTAAAAAAATAAATAAATTAGATCTCATTCATCAAAAAACTAAAAACCTGCATGTGAAATATTTAGATAGATATCAGCAGATGTCTTTAAGAAAACTAATCATGTGAGTGTGTGTGAGCTTGGCTGACGTTCAAAAAGGCTTCGTTTCTATTATTATATCATTTGCTGTTTTGTCAGTGGTAAAATTTGGGTCTATATTACCCACTGACCTTTGACATATATAGATGAGCAACTATATTAAATTTAATTTTATTAGATTTTGTTATTTTGTCATTTTCCTCTGCTGTGTTCATCAGTTTATCAGCTCCACAGACTCTACAAACTGGGACTCATTTCCACTTTTGCTTTTTGCTTAGCGTGAATTTTGTCGTCACCACTCTAAAGGTCACGTATATCCCTTTTTAAGTGCTGCATGTGTTTTCTTTCTGCGTCCGCCAGGAGGGAGTCAGTTCCGTGTGTTTCTTCTGCTGAAGGCTCTGCAGACGGTGGCCCAAACGTACGACGCGCAAAGAAAACTGCGGGCCACCAGAGCAGACCCCAGTTCGTCAGTGAGGGGCGGCGTCTGTGGGCTGAAGGCTCTCACCGTGTCCCTGACAAAGCTTCTTGTTGGCCCAAGCAGCGCAAACATTAACAATTGCCACGGCTCCTGCGCGTTCCCTCTGACCAACGGCAACAACCACGCCATCCTGCTCAACTCCCACATCGAGACCGGCAACGCGGATGAGCGTTCGCCCTGCTGTGTGCCCGTGGCATACGAAGCCCTGGAGGTTGTGGACTGGAACGCAGATGGGACCTTCATCTCCATCAAGCCAGATGCGGTTGCGAGGGAGTGTGGATGCCGCTAGAGCTGCTCTCTTCTGCTACTTTACCCCAGCATTTATAACTTTCACACAACTGATTAGTTATGCGCATGTTTCAGTTATGAAATATCAATTACAACACACCACGTGTACTTCTAATTAAATATAACTGCTTTTATGCTTTATTAAAGATCACAAGTCTTAAGATCACAAAGTTAATGTTAATTCTATTTATGTTAAGATATTTTGATAATTAGTGATGCTAAAATGAATCAGATTTTGTCATTTTGAAAATATTTCTAATTGGAATAATGTTTCTGAGTGTTAAAGTTAAACTGTTAAAAGGATGAAATATAGCCTTCTCAAACTTGTAATAATTAAATGCAAAGTGTACTTAAAGTTCTGTGTTCGTAGCCATTCAGCTACATAAGCTACAGTATATGTTTTATGCTTTGATTTTGGGTTTCAGGATTGCTTTATTAGCAGCTGGCTTCTTCCTCCTTCTTATCGCTCCGACTTCTTCAGATCCCCACCACCGGGTGAAGCCGTCCCATATGGAAACCTGGAGGCAGACAATGGGACACAAGACACGTGTGATTCCATATCAACCAGCCCTAAATACTGTGGAAATCCTATAGTGTTGCATAATAAAAATTCTCATATATCATAATATTTGAGCATGTGATATTTAAAGAGATGATCCAAAGTCCAGTTTATTTTTGGTCCTCTGAGCTGTAGAGCTGTTTGTCCAGCTTGAGCAGTTTAGTGTGCATTTCCTAGACTTGGAGAAACCGGCTGTAGACATTTCTGCGCTCTTTGGAACATTTATGAAAATTATTATTGCTATATTATAAAATTATTAAGCACACATCTTGCAGTACCAAAAATACGATAATGACTCTGTTACTCTCAAAGATAGCTGTGAAAAGTTCGGCATCCATCACCCAAAGAAGTATACACTAACCTGTGATAGCCAAGGTTATTACTGTTAACTAATACTAACTTGTTACGCCGCTCTGCAGCCCCTAATCTCTTTGCAGTGGCAGTGCAAGAGTGTTCAGCTGTTGCTAATTGACCACACTTAATCAGGTGTGGATAAAGGCATACTTGAGGCAGGCTTTCATGGAGGCGCACTAGTCTTTGTCTTAGTGGTACCAGCTATTTTAGCTAACTTATTATATAATTTAAAATAAAACCTCTTCTCTGTGAAATTCTGGTGTTGGTCTCCTTAATTATGTTGCAGCTTTTGAGCTGGCTCGTAACATAACTAAAATTAAAATATAGAGGAAATATTGTGGAGTTTTAGCATCTGTTAATCTCGTTTAACCTGTCAGCTGATGAGGTTTTGATAAACGTGTTTGAGACTTTGGATCTCTACCAGACAAAAACTTATAGTTTGATTGAAATATATGCTCTGCTTTTTCTAAATCTTACCTCTGACATACAATAAAAGGTTAAAACTAACACTGCACCGAATAAAAACTAAACTAAACATTTTCGAGCAGTAAAAACAAAAAAGTCAAAATAAAATAAAAACTTATTAGAGAATACAAAAAACTATAATAACTCTGGTGACAGGATTTAATCGTGGCGCCACCCTTCAGCTGAGCTACAGCAAGCTGCAAACCTCTTTTGGTTGGTATTTGTAATTCTCTCTGTTGTGAGCATGAAACCGCTCACAACAGTCTCATTGAGGCATACATCTCCAAAAATGGGAAAATCACACCAAAATAGTCCAGATGGGTGCACAGATGATGTCATCTAGACACAGGATGCACTCTGATGTCTTTAACGCTTAAAAACATGTGGAAGAATCAGAAACGTGATCTTGATTGTACAACATAAAAATCTTGATGTATGTAAAATAAGACACACTATAAGATACACTTCTAATGTTGTTCTTTTAACACACAGAAGCTCCAAATGCTGTTCACTGCTCTAGTCCACAGGATCACCAGGCAGCTCTGCTCAGTACACCAACACCTGCACCGTACACAGTGTTTCATGCTTTATGCTTTGGGTTGAACAGCTACGGCTATTGTCAATCTTGCAAAACTTTATTTTTTCATCCATGAGAGGGTAGGTTTGCTCATTCAGTCTTTAAAAATGGGAATAAAATTGTTTAAACCAGTAAGATTACATGTAAGGAGATATGGAGCCATCCAGTGGTTCATCAGCCGTGTGTTGACCGGGTATTTGTTAATTCTCAGCCATACAAACGGACACTTTACGGTTTCCTTATTTCCAAATCTGACAAAGACTTTCTCAAAATCTCTGTGAACTTACAGCCGGCCCATTATTCTCCATCTCACAGTGTTTATCCTACTGTTGTTGCTTTATGATGCCAGGACAATATGGCCCTCTCAGACTGATAGCTTGTCACTGAGATGCTTTGAACAGCAAGAGAAGAAGATGGATTACGGCTTTAAAGGCAGAAAAGCTAAAATGGCTCATTTCAGACAGCGGATGAACTGAAGGGTTGCGCTCATTAAAGAATAATGTTTAAGATATATGATTAAAAGTCCAAGTACTAAAATACCGAGCTGGAAATGAGCACGACTGTGTATTCAGCCAGAAAACAGAGTAAAAAACTTTACTGTATCTGTAAATTACACATAAAATCACCCTCAGTCTTTAAACAAGAACAAATGAACCGAAAGTTGCAGGCTAACTGACAGAATCACAACGAGAAACCATACCTGTGTTATTGAAACACTCTGCAGACTTCCAAAGAAAGACCACACACCTGTCTCTTCAACAAAATAGTCCAGAGCATCTAGAAAACACAATTGCACAAAAGACTGAGAGCGTGTTAAACAGAAGCTGTCGGCTGTTACAAATCACAGCAATCAGTCAGAAACAAGCTCAAAACATGTGTGATGTGGGTAAAGAGACAGTGCACCGGCACTCACCGTGCACTTCGTTAACTCCTGAGCTCACCAGTAGCACAACCAGCGCCGCCACTAAACAGCGGTTCATAGTGAATCCTTTCCACGGATTTGGAACATCGCTCAGATTTTCAATGCACAGAATCCGCTCATAAGACACTGCAGACAGACACGAGTCAGACGCATTTAGTCCATGCTCAGTTTCATCAGTCTGGTGGCTGTTTAGTGAAACGTGTGTCTTACTCGTGAAGCCTTCAGACAGGCCTGCTCCAGCTTCTTAAGGCTGAAAAATAACACTGCAAGATGAGGAAAGATTGGGTAGGACTGTGTGGTTGAGATTTAGTAAAGTAGCAGTACACAGAATACACAGAATGATCACACGTGGAGTTTGTTTAATCCCCGATATCTTATATCTGTGATCATATCCCACTTTCCCCTGTACTGCCTTTTGCACCCTCTTCCAAGCCATACACTAACATGTGACACTAACACAGCTTAAAATAGTCACATGCAGCTCTTTAGAAAGACAAATATGGAGCTGAGCGTTTAGTTTGATACAAACCTCTGGACAGAAAAATACGCTTACCTGATTTTCAGTTGTGCTTAACCACAGAGGTGAGGGATAGTAGAGCTGTGCTGTTGCGGTTGTGTACACGCTCTGCACCTCGCTTCTTTGCTTGATTGACAGGCACAGGTGTGAAAACAGGGAAAGGGACATTCAAACTTAATAGCAGTAAGCCAGTAGGTGAACGAGGATCAGCAACACCCGCCTTCCAGTGCCATGAACACTCCTACTCCTCCACAGAGGCAGATAAGCCAGCGTTACTAGGGTGGTTTAACAGTTTACTTAATTATGAGGAGGTCCTAAAACACAATACATTGTTAAAAGTTCATCACATAATAAATTAGTCCCAATTCAAATTAAAGCAGGCACCAACAGTGTCATCCCCAGAGCCATCCACACCCTCGACAAACCCTCAGTGATTCTGTATGTTACTAAATTCACATGACACTGATGTGTATGATGTATGATTGGGTACCTGCAGTCTCAGTGTATCGCTGTATATAGTGTCAATAAAGGCATCATCAGCAGTAATAACTTCTCCTTCTTCTTTCAATTCTTCTTTCGGCTGCTCCCTTCAGGGGTCACCACAGCACATCATCTGCATCCATTTCATCCTTTACCTACCATCGTCTTGTCACATCAACCCTCTGGAGGCCTTCCTCCGGCCTGGCAGCTCCATTTTTAACAGCCTTAGTCCAGTATATCCATATCTCTGCTCTGCACATGTCCAAACTTTGTCTGGAGCAGTCCAGTCTTTTTGATCAGCAATAATAACCTATTACTCAAATATGTACTCGGCTAAACCATGGTCCACAGTACCACTGCACATAGCTGAAATGACAATAAAGCCACTTGACTTGGCAAAAATTATATAACTCAATTTTAATTTTTGGCATAAATTATATATATATATACAACAAAAGACAAACTGAAAAAAAATTATGCTAATTCTACACAAAACAAGACCGTCTGTACCAGGGGTACGCAATAAGTAAGTAAAATTTATTTATATAGCGCTTTTCACAGATAAAAAATCACAAAGTGCTTTACAGTACAAGAAATGGAAATATAAAACAATGTAACCGTAAATAAAAAAGCAAGGTAAAACAATAAAGAAAACAATAAAAACAACGTAAGAACAAATTAATCAAAAGCTTTTCTATATAAAAATGTCTTCAGCTATTTTTAAAAGATGTGCCCAATCTAAATCCCAATCTAAAACCCAATGTATTACTACTTAGATACTCTTTTGTGATATAAATGTTTTTATACTGAACGCGTTGTTTTATTGTTGTATATTTTATTACACTGTGAAGTGACGGTGCTGAGTAACTGCGTAACATTTCGACGGTAAACGTCAGAGTGACAGAACACGCCTCCGCCAAATACCGCAGCTCCCCAGCTTAGCCCAGCATACGGTGACGAGGAGAGGACAGAGGGAGCAGCTGGTGTGGACGCTGGCCGGATGGTTAAATCTAACCTACAGACGATTTTGAATAGTCACTGTTTTGCTAGAGAAAAAGAGAGAAACTTACCCGAGATGCCTGTTATCGAGAAGTCCAGTAATAAACCTGAAAGTGAAAGGTATGTTGTGGTCCGTGTCAGCTGCTCTCAGTGAGCTAAGCTAAGCTACGTTTAAAAACCGCGGCCTAGCTTTCGCATAAGCTGTGAGGCGACTAGCACAGCCCTTCATGTTTACATAACAGCGGCACTTATTTCAGTTGACATTTATAGAGAACATTATTAATCACGGGCAGCTTATTTCAATTCCATAAAAGATGCATATTAATCACCTTAAAATACATCGCAGTAGCGGGATTATGAGCAAAATGTTAGAAGTGACAGCGCTGCTTTGGTAGCTTCACAGATGCTAACGCGTCAGCTAATATGTCAAACGCCACAGGTTCGCGTTATCGATGGCTGGGCTAACGTGTCGTAAGGTTTTCTATTAAGACCTGGTAGCGACTAGAATTGTAATTTATAACATTTAAAATACAAATTTTGCGTGTTAAAAAGCGGAATAAGAGTGTTTTATCGTCACTAGGTGTGTTTTCCCCTTAAATATTAGCGTAGACATTTAAGCTAAATGCTAGCTCTTTAGCATTAGGTAATCTGACGGATGCGTTCATATTTTCGTCCTTCGCAATGAAAATGATCAATGGCTTTGAAACCTCGTTACTATCTTTTGTGATGTGGCTTTTGTGATGTAGAACTAGTACAAAAATATTTGCGCGGGGTTATGTAAAGTTAGCTAACAATGTAGTTAAAATGATGGCTGTACGGATTAATTCGTGGTTTCAGGGATTTTCTTATTTATTTATATATTTTATTTTATTTTTCCCTTTTCGCCCTTCTGAACCCACGATAAGACATCCAGTAACTTCTGGAAAACACGGGTTCCAGGTGGGCGGACAGGGAATACGTTTAACCGACGCTGAGAAGAACCTTTGTAATGTTTTTAATCAGATTCGGTTTGATTTATGCGAGCTGCTGCGGGGCCATTCTTCAGCCAGCCGTGTGCCTGTTTGGGTGCAGTTAAAGTACCGATTGACTGGAAGTGAGGGGGCTGGTAGGGGTGGATACCTCGAAACTGGTTGTTAGCGATTAACCCAATCTGGTGGTCGGTGGTTGCACCACTCGTGCCCTGACAGGATTTGTTGACGCGCTCTGTTTGTGCTGATCTATAGTGTTTGAGGCACTCATAGGAAATTTCCACTGCGGGGGAGGAGGGACGGAGGGGGGCAGGGTGGGTGCTGTATGGGGAATTTTGGAAGTTATGGCCTACACACAAATCTCGATGTATTTTTGCTTATCGTGGCAAGTAGTTGGTTGGAACTTGGATATAGTTATGTAATTGTCATGGTTAGATATTTATATATGTATATTATATAATAGTGTTTCTCTTTAAGCCAAAGCTTGAAAAGATGCCTTTATAAATATGCTGACACATAGCTATCATGTGTGATAAGCCTGGTTTAACATCCACACCTTCCAATAATAAAGCATGCATTAAAATAACATCATC19TATTATAGCAGCTAATAGCCTCCCCCCTCCCAAGGTAAAAAGTCTCTGTTACCTTAAGAGAAGGCTAGGCCTTTTCTTTTCATTCTTTTCATTTTTGACCACCTGATAAAATCAAATGTATTTAAACTTGTGTAAATTTAACCCCACATTAAAGACTTACATTTTTAACCTGCTAATTTTTCTTTTGTTGAAAGCCAGAACGTGTTAGGTGAGCTAAAATAAAACCCACATTGCAAATGTCATACTACATTGAAGTGTCTAACAGTGCAGGCTAGAACATGGGATTAAAGF25ATGATTCTCTGCTAGATGGATGCATGAGATTTATTGCAGATGTCCTTTACAATGATGTTCATTTACAGCAGAGTCTCCTACAAACACTACAAACACTATTCACAGCAGCTGTTTTTATTAAGATGACCGTCGGGGCGTCATTGCGR18CAGCACTGCCTACTGTTCACATGTCAGTCTCAAAGAATGCTAAAGGCAGAAACTGACTGAGAACTTTGTGTTCTCTTTAAATCGCAGAAAGGAGGCGTTAAGGGGTTCTGCAAGGATGGCAAGTGGCTTCCTTTCCTAATCTGATTACCGGATTTAGGGAGTAACTGCATTTGGTTAAAATCCCCAATCCTCTGTGAGATCATAAGTTGGATATTACACACTGCGAATGACACTGGTTTCTACACCTCTGCTCAACATGTAGCGCATTGGTGGCATTTGCGTAAGAGAGCATCCCTGTGTGTGATGGTGTTTGCGGCGTTGTTATTTTTGTTCCTAATAGAATCCCAGTGAACAGGCCTTCGCTCAAAGCTGTAAATGTATGCAGTCACAGCAAGCTAAATCTGAGGAGTGCTGTGACAGCGCTGAATTAGAACGGCTGATGAGTATTGTTTGTCCCAGTCTCTTAAACTGCAGTGACGCGGTGAGTGAAGAGCTAAAGTAGGCTAGCGGCTAGAGGAAAAAAATAGCCTGTTCTTCTGGCAGGTTTCCTGTGAGAACTGTGGAACAAGCTTAAAGTGCCCGCAGCAGAAGTGTGCAGAAGCCGTAGCCTCGATGTGTCTTTGTGAGACAAAAAACAAACAATTTCTTATAGTTGGTGGTCAATATTTGACTGACAGCTGACATAAAATTGTTAACAGTTTTAGTGAGGCATCAGTAATGTGGTGGTGTGACATAAACCACTTCTCCTTTCCGTTACGTGTTCCTCATCCGTCGGCACGTCATGCACGCATCTGTGCTCAATGTAGGAACGGTTATTGATTGAACCGGTTCCCCCAGTCTTGTGCTTTTGTGTCTGAAGACTCACTCTGGGTTAGTAAATGTTTTACTGCAGTATCACAGATGAGTCACTCGGCAGTTTAATGTGTGCCGAACCTGCTGCTTATAAAGAGTTTGGTTTGTGTTTGGTAGCAGAATCTCGACTGAATACCCAGCTCATGATGGCTCCATCTGTGTGTGCGTTTGTTTCGCTTTCCAGTATCTCCAGTCCTCTGAGGTGCTCCCGCTGTTGCAGTAACCCGTGTCCGGGGCCTCTGTGGTGCTCCTGATGCCCCTCTCCCACCCCTGAAGATCCCAGGTGGGCGAGGGAATGACCAACGGGATCGCAATCTTTCAGCTAAGCTGTT
